# A single-cell rice atlas integrates multi-species data to reveal *cis*-regulatory evolution

**DOI:** 10.1038/s41477-025-02106-6

**Published:** 2025-09-17

**Authors:** Haidong Yan, John P. Mendieta, Xuan Zhang, Ziliang Luo, Alexandre P. Marand, Yan Liang, Mark A. A. Minow, Yun Zhong, Yarong Jin, Hosung Jang, Xiang Li, Xinxin Zhang, Thomas Roulé, Doris Wagner, Xiaoyu Tu, Yonghong Wang, Daiquan Jiang, Silin Zhong, Linkai Huang, Susan R. Wessler, Robert J. Schmitz

**Affiliations:** 1 Department of Genetics, University of Georgia, Athens, GA USA; 2 College of Grassland Science and Technology, Sichuan Agricultural University, Chengdu, China; 3 College of Life Sciences, Shandong Agricultural University, Taian, China; 4 Department of Biology, University of Pennsylvania, Philadelphia, PA USA; 5 Joint Center for Single Cell Biology, School of Agriculture and Biology, Shanghai Jiao Tong University, Shanghai, China; 6 Institute of Genetics and Developmental Biology, Chinese Academy of Sciences, Beijing, China; 7 State Key Laboratory of Agrobiotechnology, School of Life Sciences, The Chinese University of Hong Kong, Hong Kong, China; 8 Department of Botany and Plant Sciences, University of California, Riverside, CA USA; 9 Present Address: Department of Molecular, Cellular, and Developmental Biology, University of Michigan, Ann Arbor, MI USA

**Keywords:** Epigenomics, Plant evolution

## Abstract

*Cis*-regulatory elements (CREs) are essential for regulating gene expression, yet their evolutionary dynamics in plants remain elusive. Here we constructed a single-cell chromatin accessibility atlas for *Oryza sativa* from 103,911 nuclei representing 126 cell states across nine organs. Comparative genomics between *O. sativa* and 57,552 nuclei from four additional grass species (*Zea mays*, *Sorghum bicolor*, *Panicum miliaceum* and *Urochloa fusca*) revealed that chromatin accessibility conservation varies with cell-type specificity. Epidermal accessible chromatin regions in the leaf were less conserved compared to other cell types, indicating accelerated regulatory evolution in the L1-derived epidermal layer of *O. sativa* relative to other species. Conserved accessible chromatin regions overlapping the repressive histone modification H3K27me3 were identified as potentially silencer-like CREs, as deleting these regions led to up-regulation of gene expression. This study provides a comprehensive epigenomic resource for the rice community, demonstrating the utility of a comparative genomics approach that highlights the dynamics of plant cell-type-specific CRE evolution.

## Abstract (web summary)

This study maps chromatin accessibility at single-cell resolution in rice and related grasses, revealing how regulatory DNA elements evolve across cell types and species and identifying potential silencers that control gene expression.

## Main

*Cis*-regulatory elements (CREs) function as pivotal hubs, facilitating transcription factor (TF) binding and recruitment of chromatin-modifying enzymes, thereby fine-tuning gene expression in a spatiotemporal-specific manner^1^. CREs play important roles in developmental and environmental processes, and their functional divergence frequently drives evolutionary change^2,3^. Previous studies highlighted the dynamic nature of CREs throughout evolution and their involvement in regulating gene expression via distinct chromatin pathways^4–9^. Across diverse cell types, gene expression is intricately regulated by multiple distinct CREs, each exerting control within a specific cell, tissue type, particular developmental stage or environmental cue^10–12^. In plants, environmental sensing and adaptation relies heavily upon epidermal cells^13^. For example, grass epidermal bulliform cells change their turgor pressure to roll the leaf to slow water loss under stressful conditions, with the TF, ZINC FINGER HOMEODOMAIN 1 (ZHD1), modulating leaf rolling by influencing rice (*Oryza sativa*) bulliform cell development^14,15^.

There has been increasing use of single-cell assay for transposase-accessible chromatin sequencing (scATAC-seq) to identify cell-type-specific CREs within diverse plant species^16–24^. Our recent study highlights the dynamic and complex evolution of CREs in C_4_ photosynthesis, particularly in mesophyll and bundle sheath cell types^25^. However, despite these findings, our understanding of CREs exhibiting evolutionarily conserved or divergent cell-type-specific activities remains limited. Moreover, the specific TFs and the motifs that are associated with conserved and derived CREs remain unelucidated.

Much focus has been placed on enhancer CREs, yet silencers are equally important, as they repress gene expression until the proper developmental or environmental cues. Our previous research uncovered that some accessible chromatin regions (ACRs) flanking trimethylated Lys 27 of histone 3 (H3K27me3) are linked to the suppression of nearby genes across species^6,9,20^; however, whether these H3K27me3 ACRs include potential silencers remains undemonstrated. A notable limitation lies in the lack of single-cell resolution for H3K27me3 ACRs. Bulk analysis of H3K27me3 ACRs reflects only the average chromatin accessibility status, indicating potential silencer activity under most cell types or conditions, but obscures instances of cell-type-limited H3K27me3 removal. To date, cell-type-resolved ACRs near H3K27me3 have not been identified, leaving the role of these regions harbouring candidate silencers unresolved.

Rice serves as a staple food for half of the world’s population and a key model species for monocots and crop research^26^. However, a comprehensive single-cell chromatin accessibility atlas for *O. sativa* has yet to be established. Through scATAC-seq, we constructed an expansive single-cell reference atlas (103,911 nuclei) of ACRs within rice. This atlas associates agronomic quantitative trait nucleotides (QTNs) with their cellular contexts and provides novel insights into seed and xylem cell development in rice, serving as a valuable resource for exploring cell-type-specific processes. By integrating H3K27me3 data, this atlas finds a series of conserved ACRs and the candidate CREs within them that are potentially important for recruitment of Polycomb-mediated gene silencing. We then leveraged these data in tandem with four additional scATAC-seq leaf datasets from diverse grasses (*Zea mays* (16,060 nuclei), *Sorghum bicolor* (15,301 nuclei), *Panicum miliaceum* (7,081 nuclei) and *Urochloa fusca* (19,110 nuclei))^25^ allowing us to compare ACRs across species and cell types. We quantified the proportion of ACRs that were conserved in these monocots and found high rates of cell-type-specific ACR turnover, particularly in epidermal cells in the leaf. This indicates that the ACRs associated with specific cell types are rapidly evolving. Finally, we developed a database (RiceSCBase; http://ricescbase.com) to facilitate the evaluation of chromatin accessibility, associated genes and TF motifs in the rice atlas.

## Results

### Construction of an ACR atlas at single-cell resolution in rice

**Fig. 1:**
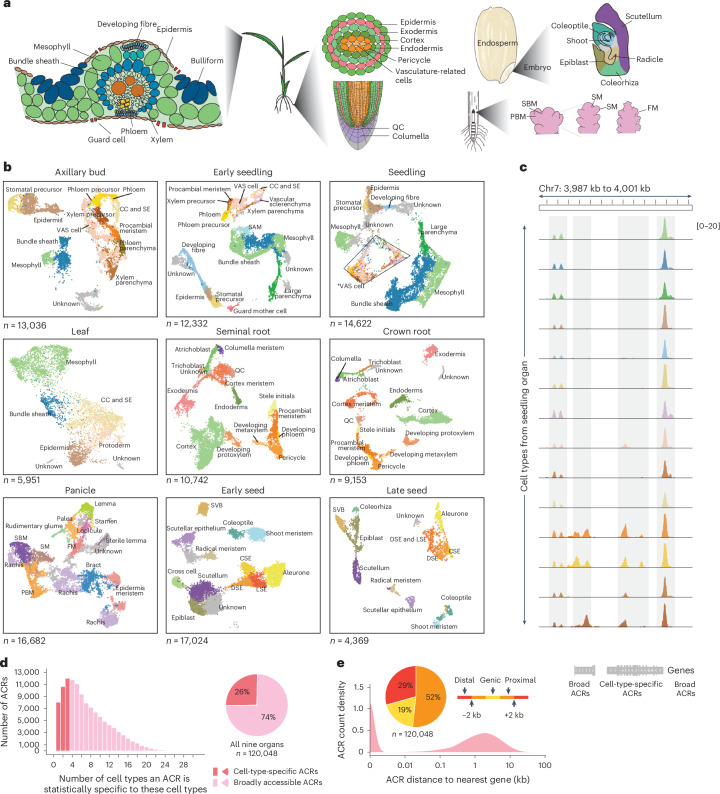
Identifying cell types and characterizing ACRs in rice using scATAC-seq data. **a**, Overview of cell types in leaf, root, seed and panicle organs. SBM, secondary branch meristem; PBM, primary branch meristem; SM, spikelet meristem; FM, floral meristem. **b**, UMAP projection of nuclei, distinguished by assigned cell-type labels in axillary bud, early seedling (7 days after sowing), seedling (14 days after sowing), leaf (V4 stage; four leaves with visible leaf collars), seminal root, crown root, panicle, early seed development (6 DAP) and late seed development (10 DAP). SAM, shoot apical meristem; VAS cells, vasculature-related cells; *VAS cells, vasculature-related cells that were further distinguished as procambial meristem, developing phloem/xylem, developing phloem/xylem precursor, vascular parenchyma/sclerenchyma, xylem parenchyma, and companion cell and sieve elements in Supplementary Fig. 6; CC and SE, companion cell and sieve elements; CSE, central starchy endosperm; DSE, dorsal starchy endosperm; LSE, lateral starchy endosperm; SVB, scutellar vascular bundle. **c**, A screenshot illustrates the examples of cell-type-specific and broad ACRs. **d**, Evaluation of proportions of ACRs that are cell-type specific versus broad. **e**, ACRs show a bimodal distribution of distance to the nearest gene. The ACRs were categorized into three major groups based on their locations to the nearest gene: genic ACRs (overlapping a gene), proximal ACRs (located within 2 kb of genes) and distal ACRs (situated more than 2 kb away from genes).

**Extended Data Fig. 1:**
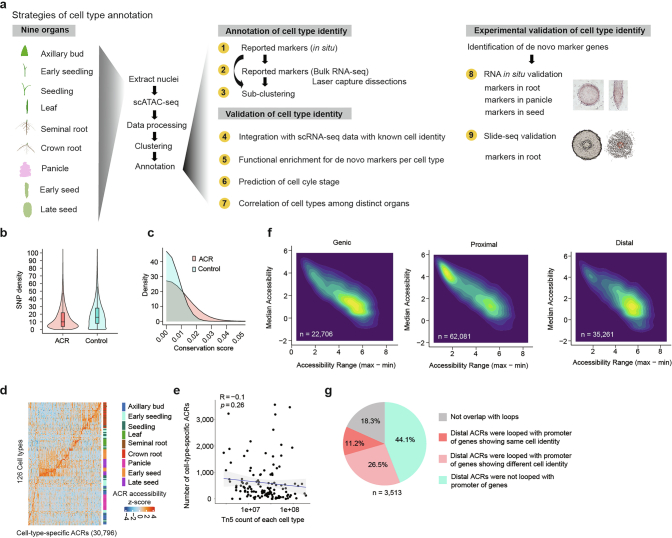
Pipeline of cell identity annotation and quality control of rice ACRs. **a**, A pipeline of cell identity annotation corresponding to nine strategies shown in Supplementary Fig. 1. **b** and **c**, A comparison between ACRs (n = 120,048) and control regions (n = 120,048) in terms of SNP density (**b**) and conservation scores (**c**). For panel **b**, the center line indicates the median; the box limits indicate the upper and lower quartiles; the whiskers indicate 1.5 times the IQR; the dots represent the outliers. The SNP data were downloaded from Rice SNP-Seek Database^149^. The SNP density is the number of SNPs per 1,000 bp within the ACRs or control regions. The ACRs exhibited lower SNP density and higher conservation scores compared to a control composed of non-ACR DNA. The control set was created to match each ACR by excluding exons, ACRs, and unmappable regions, and they possess a similar GC content (< 5% average difference) compared to the positive set. **d**, Row-normalized chromatin accessibility z-scores for ACRs across cell types. **e**, Two-tailed spearman correlation is shown between the total number of Tn5 insertions and number of cell-type-specific ACRs across all cell types. **f**, A two-dimensional density plot illustrates the median chromatin accessibility across 126 cell types for 120,048 ACRs, along with the range of chromatin accessibility (calculated as the difference between the maximum and minimum values). **g**, Categories of distal cell-type-specific leaf ACRs showing potential interaction with genes based on examining chromatin loops derived from rice leaf Hi-C data.

To create a cell-type-resolved ACR rice atlas, we conducted scATAC-seq across a spectrum of nine organs in duplicate (Fig. 1a,b). Data quality metrics, such as correlation between biological replicates, transcription start site enrichment, fraction of reads in peaks, fragment size distribution and organelle content, revealed excellent data quality (Supplementary Figs. 1 and 2). Following strict quality control filtering, we identified 103,911 high-quality nuclei, with an average of 41,701 unique Tn5 transposase integrations per nucleus. Based on a nine-step annotation strategy, which included RNA in situ and spatial-omic (slide-seq) validation of cell-type specificity, we identified a total of 126 cell states, encompassing 59 main cell types across various developmental stages from all the organs sampled (Fig. 1b, Extended Data Fig. 1a, Supplementary Section 1, Supplementary Figs. 3–14 and Supplementary Tables 1–7).

By analysing cell-type-aggregated chromatin accessibility profiles, we identified a total of 120,048 ACRs (Extended Data Fig. 1b,c). Among these ACRs, 30,796 were categorized as ‘cell-type-specific ACRs’, exhibiting cell-type-specific entropy signals of accessible chromatin in less than 5% (3/59) of the main cell types, whereas approximately 89,252 were classified as ‘broad ACRs’ with chromatin accessibility in more than 5% of the cell types (Fig. 1c,d and Extended Data Fig. 1d). The identification of cell-type-specific ACRs was independent of sequencing depth, as evidenced by the lack of correlation between Tn5 integrations and the number of cell-type-specific ACRs per cell type (Extended Data Fig. 1e). When analysing ACR proximity to genomic features in the rice genome, about half of the ACRs were gene proximal (52%; located within 2 kb of genes; Fig. 1e). These proximal ACRs had higher but less variable chromatin accessibility than genic and distal ACRs (Extended Data Fig. 1f). By contrast, about 19% of the ACRs overlapped genes, mostly in introns, and the remaining 29% were categorized as distal (Fig. 1e; situated more than 2 kb away from genes). The greater chromatin accessibility variance in non-proximal ACRs suggests these regions may act in select cellular contexts. To further investigate the association of distal ACRs with gene activity, we examined the interactions between distal cell-type-specific ACRs and genes using leaf bulk Hi-C data^27^. Among the 3,513 distal cell-type-specific ACRs in leaf tissue, most (81.7%) were embedded within chromatin loops. More than one-third (37.7%) of these ACRs interacted with promoters of cell-type-specific accessible genes, and 11.2% (392) had both the ACRs and their interacting genes associated with the same cell type (Extended Data Fig. 1g and Supplementary Table 8). As bulk Hi-C poorly measures rare cell types, we expect this number to be a conservative count of the number of cell-type-specific ACRs associated with cell-type-specific gene activity.

### The atlas uncovers key TFs, their motifs and ACRs during rice development

**Fig. 2:**
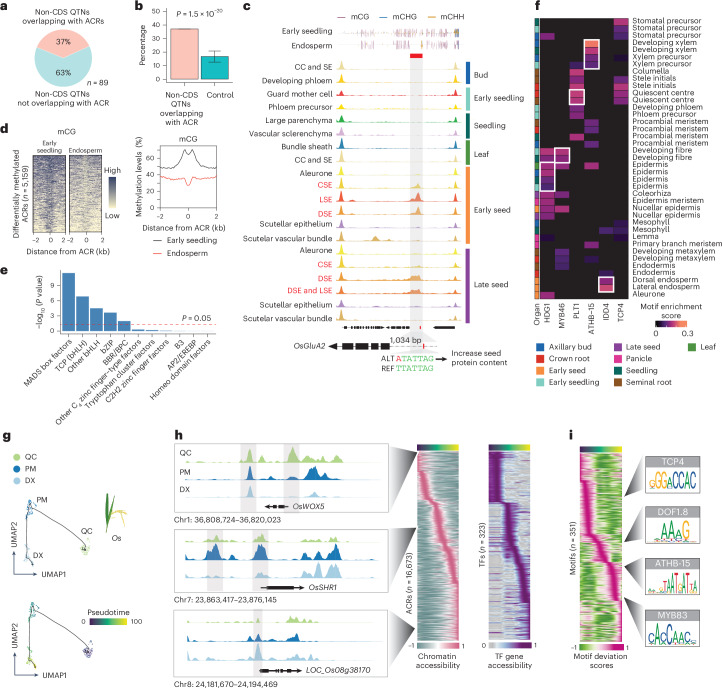
The rice ACR atlas contextualized agronomically relevant ACRs and key TF motifs during cell progression. **a**, The ratio of non-CDS QTNs overlapping with ACRs to all non-CDS QTNs. **b**, Percentage of non-CDS QTNs overlapping with ACRs. The significance test was done using one-tailed binomial test (alternative = ‘greater’; see ‘Construction of control sets for enrichment tests’ in the Methods). The error bars indicate the mean ± s.d. **c**, Analysis of cell-type-aggregate chromatin accessibility across 12 seed cell types and eight non-seed-related cell types, showing signatures of a QTN within an endosperm-specific ACR situated at the promoter region of *OsGluA2*. An endosperm-specific reduction of cytosine methylation was identified over the endosperm-specific ACR. mCG, mCHG and mCHH refer to DNA methylation at cytosines in the CG, CHG and CHH sequence contexts, respectively, where H denotes A, C or T. **d**, A total of 5,159 differentially methylated ACRs were identified, showing hypermethylation in early seedling tissue but hypomethylation within endosperm tissue. **e**, Enrichment of TF families based on their motifs within the 5,159 ACRs.The *P* value was computed using a one-tailed hypergeometric test (alternative = ‘greater’). bZIP, Basic Leucine Zipper; BBR/BPC, BARLEY B RECOMBINANT/BASIC PENTACYSTEINE; MADS, MCM1-AGAMOUS-DEFICIENS-SRF; EREBP, ETHYLENE-RESPONSIVE ELEMENT-BINDING PROTEIN. **f**, Deviations of motifs displaying enrichment in specific cell types (white frames) where their cognate TFs are known to be accumulated. MYB46, MYELOBLASTOSIS 46; PLT1, PLETHORA1; IDD4, INDETERMINATE DOMAIN 4. **g**, UMAP visualizations depict the cell progression of RDX in *O. sativa*. PM, procambial meristem; DX, developing xylem; *Os*, *O. sativa*. **h**, Relative chromatin accessibility of ACRs and TF genes associated with pseudotime (*x* axis). The left side displays three marker genes neighbouring the enriched ACRs along the trajectory gradient. *OsWOX5*, *WUSCHEL-RELATED HOMEOBOX*. **i**, Relative motif deviations for 351 TF motifs (left). Four motifs enriched along the trajectory gradient are shown on the right.

**Extended Data Fig. 2:**
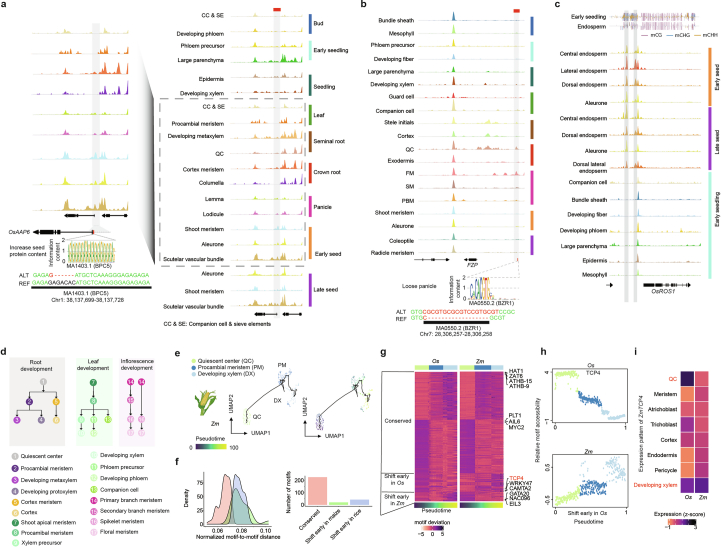
Characterization of QTNs located within cell-type-specific ACRs and pseudotime trajectories. **a**, The ratio of non-CDS QTNs overlapping with ACRs to all non-CDS QTNs. **b**, A bar plot reveals the non-CDS QTNs significantly overlapped ACRs. **c**, Analysis of cell-type-aggregate chromatin accessibility of ACRs surrounding *OsROS1* across endosperm-related cell types and cell types in early seedling tissue. The gray bar highlights endosperm-specific cytosine methylation depletion over endosperm-specific ACRs. **d**, Overview of pseudotime developmental trajectory analysis. **e**, UMAP visualizations depict the cell progression of root developing xylem in *Z. mays*. **f**, Distribution of motif-to-motif distances (left panel) and number of motifs (right panel) from k-mean groups. **g**, The heatmap displays motif deviation scores ordered by pseudotime (x-axis). ‘Conserved’ indicates motifs that showed consistent patterns of motif deviation score changes along the pseudotime between *O. sativa* and *Z. mays*. ‘Shift early’ indicates higher motif deviation scores observed at the beginning of pseudotime. **h**, Comparison of relative motif accessibility of TCP4 along the pseudotime between *O. sativa* and *Z. mays*. **i**, Expression profiles of the *TCP4* gene using an scRNA-seq datasets from *O. sativa* and *Z. mays*.

To demonstrate the utility of this new resource, we associated the atlas ACRs with a set of noncoding QTNs (Fig. 2a). We observed an enrichment of agriculturally relevant QTNs^28^, within ACRs (Fig. 2b), some of which were cell-type-specific ACRs (Extended Data Fig. 2a,b and Supplementary Table 9). For instance, a QTN was within an endosperm-specific ACR located at ~1 kb upstream of *GLUTELIN TYPE-A2 PRECURSOR* (*OsGluA2*) (Fig. 2c), which is associated with increased seed protein content^29^. Exploring the endosperm epigenome more, we observed an endosperm-specific reduction in cytosine methylation at endosperm-specific ACRs, including an ACR linked to the DNA demethylase *OsROS1* (Extended Data Fig. 2c)^30^. We found that 5,159 ACRs had lower DNA methylation in the endosperm compared to early seedling (Fig. 2d), and these ACRs were enriched for several TF motif families such as MADS box factors, TEOSINTE BRANCHED1/CYCLOIDEA/PROLIFERATING CELL FACTOR (TCP), Basic Leucine Zipper and BARLEY B RECOMBINANT/BASIC PENTACYSTEINE, compared to constitutively unmethylated ACRs (Fig. 2e). Therefore, established endosperm DNA demethylation^31^ coincides with endosperm-specific ACRs.

We further investigate the TF motifs enriched within cell-type-specific ACRs (Supplementary Table 10). We observed consistency between known TF activity and cognate motif enrichment (Fig. 2f). For example, ARABIDOPSIS THALIANA HOMEOBOX PROTEIN 15 (ATHB-15) is associated with xylem differentiation^32^, and we found a significant enrichment of the ATHB-15 motif in seedling developing xylem or xylem precursor cells. Other TFs, such as HOMEODOMAIN GLABROUS 1 (HDG1)^33^, MYELOBLASTOSIS 46 (ref. ^34^), PLETHORA1 (ref. ^35^) and INDETERMINATE DOMAIN 4 (ref. ^36^) are known to accumulate in the epidermis, developing fibre, quiescent centre (QC) and endosperm, respectively, and their motifs were enriched in these cell types (Fig. 2f). Furthermore, the motif analysis revealed potential novel roles for certain cell types. For instance, AtTCP4 regulates lignin and cellulose deposition and binds the promoter of *VASCULAR-RELATED NAC DOMAIN 7*, a pivotal xylem development gene^37^. However, in *O. sativa*, the TCP4 motif was QC enriched more so than in the developing root xylem, alluding to unknown QC roles. In sum, our TF motif enrichment sheds light on both known and novel regulatory mechanisms underlying cell differentiation and function.

To examine ACR dynamics during cell fate progression, we organized nuclei along pseudotime trajectories representing 14 developmental continuums (Fig. 2g, Extended Data Fig. 2d, Supplementary Figs. 15 and 16 and Supplementary Tables 11 and 12). Focusing on root-developing xylem (RDX), we identified 16,673 ACRs, 323 of 2,409 TFs, and 351 of 540 TF motifs showing differential chromatin accessibility along the xylem trajectory (Fig. 2h,i and Supplementary Table 13). Among the top differentially accessible genes during RDX development, several known marker genes involved were identified, including *WUSCHEL-RELATED HOMEOBOX*^38^, *O. sativa SHORTROOT 2* (ref. ^39^) and *LOC_Os08g38170* (ref. ^40^) (Fig. 2h). Early in the xylem trajectory, the TEOSINTE BRANCHED1/CYCLOIDEA/PROLIFERATING CELL FACTOR 4 (TCP4) motif was notably enriched (Fig. 2i). To determine whether TCP4 enrichment is also present in *Z. mays* RDX, we aligned the RDX motifs of both species using a dynamic time-warping algorithm (Extended Data Fig. 2e,f), which identified 62 motifs with species-differential *cis*-regulatory dynamics during RDX development, including TCP motifs (Extended Data Fig. 2g and Supplementary Table 14). It is worth noting that *O. sativa* TCP4 (OsTCP4) decreased in motif accessibility during xylem development, whereas *Z. mays* TCP4 (ZmTCP4) increased along the RDX trajectory (Extended Data Fig. 2h). This mirrors the single-cell RNA sequencing (scRNA-seq) expression patterns of *TCP4* during RDX development^40,41^ (Extended Data Fig. 2i). This revealing of opposing developmental TCP4 motif accessibility gradients in *O. sativa* and *Z. mays* exemplifies how our atlas can merge with existing and future data to drive discovery surrounding conserved and divergent monocot development. In sum, the *O. sativa* atlas provides a comprehensive ACR resource, capturing known agronomic QTNs and bringing novel insights to seed and xylem development. Beyond the discoveries outlined here, this atlas represents a potent reference for the rice research community to answer diverse questions about cell-type-specific processes.

### The atlas identifies broad ACRs enriched with candidate silencer CREs

**Fig. 3:**
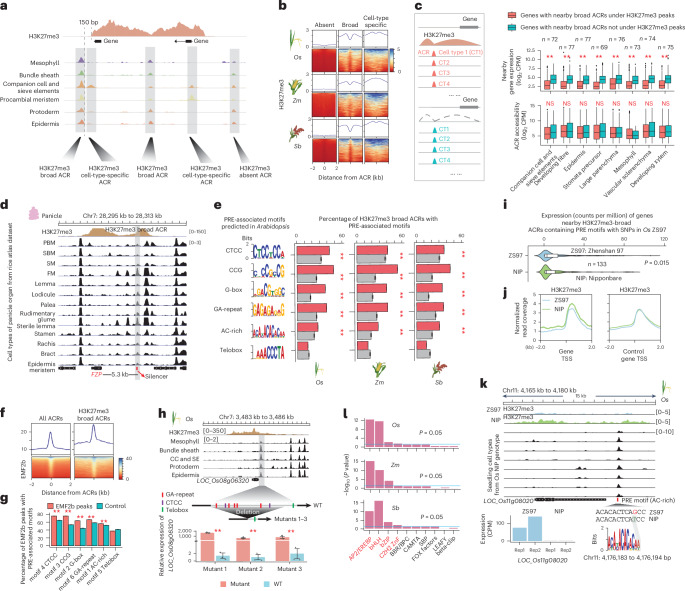
Discovery of candidate silencer CREs using rice ACR atlas. **a**, The classification of ACRs based on their proximity to H3K27me3 peaks. We first subdivided ACRs into two groups: H3K27me3-associated ACRs (found within or surrounding H3K27me3 peaks) and H3K27me3-absent ACRs. The H3K27me3-associated ACRs were further divided into broad ACRs, characterized by chromatin accessibility in at least five cell types, and cell-type-specific ACRs, accessible in less than three out of six examined cell types across all the species. **b**, Leaf H3K27me3 ChIP-seq reads near summits of distinct ACR groups. *Zm*, *Z. mays*; *Sb*, *S. bicolor*. **c**, A comparative analysis of expression levels and chromatin accessibility of genes surrounding broad ACRs under and outside of H3K27me3 peaks. **P < 0.01 (ranging from 2.8 × 10^−34^ to 7.4 × 10^−19^), which was performed using one-tailed Wilcoxon signed rank test (alternative = ‘greater’). The broad ACRs where the H3K27me3 region overlapped >50% of the gene body were positioned within 500 to 5,000 bp upstream of the transcriptional start site of their nearest gene. ‘*n*’ represents the number of genes analysed. The centre line indicates the median; the box limits indicate the upper and lower quartiles; the whiskers indicate 1.5 times the interquartile range (IQR); the dots represent the outliers. NS indicates not significant. **d**, A screenshot illustrating an H3K27me3-broad ACR harbouring a reported silencer within an H3K27me3 peak in the panicle organ, located approximately 5.3 kb upstream of *FZP*. **e**, Percentage of H3K27me3-broad ACRs in *O. sativa*, *Z. mays* and *S. bicolor* capturing six known motifs enriched in PREs in *A. thaliana*. **P < 0.01 (ranging from 1.2 × 10^−12^ to 5.2 × 10^−5^), which was performed using one-tailed binomial test (alternative = ‘greater’). Grey bars represent the control which was set by simulating sequences with the same length as ACRs 100 times (see ‘Construction of control sets for enrichment tests’ in the Methods). The error bars indicate the mean ± s.d. **f**, Alignment of H3K27me3 and EMF2b ChIP-seq reads near summits of distinct ACR groups in *O. sativa*. **g**, Percentage of EMF2b ChIP-seq peaks in *O. sativa* capturing six known motifs enriched in PREs in *A. thaliana*. **P < 0.01 (ranging from 7.1 × 10^−17^ to 1.5 × 10^−3^), which was performed using one-tailed binomial test (alternative = ‘greater’). The error bars indicate the mean ± s.d. **h**, Deletion of a rice H3K27me3-broad ACR significantly increased the expression of the nearby gene *LOC_Os08g06320*. **P < 0.01. Significance testing was performed using one-tailed *t*-test (alternative = ‘greater’). WT, wild-type plant. **i**, Comparison of gene expression linked to H3K27me3-broad ACRs that contain PRE motifs with SNPs in the ‘Zhenshan 97’ (ZS97) genotype using ‘Nipponbare’ (NIP) as the reference. Significance tests were performed using one-tailed Wilcoxon signed rank test (alternative = ‘greater’). The error bars indicate the mean ± s.d. Both the mutants and WT samples include three biological replicates. **j**, Alignment of leaf H3K27me3 Chip-seq reads at summits of TSS of genes derived from **i**. The control genes include genes overlapping with H3K27me3, which are shared between both genotypes. **k**, A screenshot illustrating an H3K27me3-broad ACR containing a PRE-associated motif with an SNP situated at 1.2 kb upstream of *LOC_Os11g08020* gene, which associates with lower H3K27me3 signal and higher expression of the *LOC_Os11g08020* in the ‘Zhenshan 97’ genotype. **l**, Four TF families, highlighted in red, were significantly enriched in H3K27me3-broad ACRs. The motif data were collected from 568 TFs from *A. thaliana* belonging to 24 families within the JASPAR database (ref. ^125^). The *P* value was computed using a hypergeometric test (alternative = ‘greater’). ZnF, C2H2 zinc-finger; SBP, SQUAMOSA PROMOTER BINDING PROTEIN.

**Extended Data Fig. 3:**
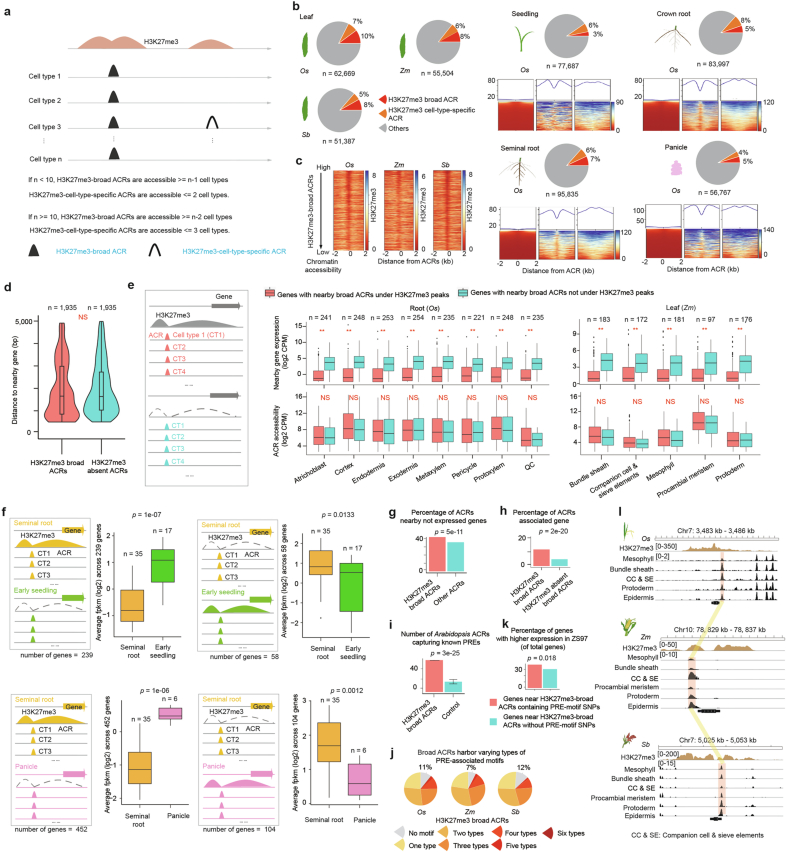
Assessment of transcriptional state of genes in proximity to H3K27me3-broad ACRs. **a**, A summary plot displaying criteria to define H3K27me3-broad and H3K27me3-cell-type-specific ACRs. For the analysis of cell types in leaf tissue across rice, sorghum, and maize, where the number of cell types analyzed is fewer than 10, we set criteria such that H3K27me3-broad ACRs must be accessible in at least n-1 cell types, whereas cell-type-specific ACRs should be accessible in fewer than 3 out of the n examined cell types. For seedling and seminal root tissues in rice, where more than 10 cell types are examined, we adjusted the criteria. Here, H3K27me3-broad ACRs are required to be accessible in at least n-2 cell types, and cell-type-specific ACRs should be accessible in fewer than 4 out of the n examined cell types. **b**, Pie charts showcasing the composition of different categories of ACRs, and bar plots displaying the number of ACRs within the H3K27me3-broad-ACR group. **c**, Leaf H3K27me3 ChIP-seq signal near the summits of H3K27me3-broad ACRs. ACRs are ranked by chromatin accessibility from highest to lowest, where the accessibility is defined as the average CPM (Counts Per Million) across all cell types. **d**, No significant difference in the distance to nearby genes was identified between H3K27me3-broad ACRs to H3K27me3-absent ACRs as shown in Fig. 3c. ‘n’ represents the number of ACRs analyzed. The center line indicates the median; the box limits indicate the upper and lower quartiles; the whiskers indicate 1.5 times the IQR; the dots represent the outliers. NS indicates not significant. **e**, Comparative analysis of expression levels and chromatin accessibility of genes surrounding broad ACRs under and outside of H3K27me3 peaks. ** indicate *p* value < 0.01 (ranging from 3.9e-44 to 4.2e-16), which was performed by the one-tailed Wilcoxon signed rank test (alternative = ‘greater’). The center line indicates the median; the box limits indicate the upper and lower quartiles; the whiskers indicate 1.5 times the IQR; the dots represent the outliers. NS indicates not significant. **f**, Four categories of gene sets overlapped with H3K27me3 peaks and comparisons of their expression between bulk RNA-seq data libraries from two different organs. The ‘n’ above the box indicates the number of libraries used in this comparison. Significance testing was performed using the one-tailed Wilcoxon signed rank test (alternative = ‘greater’). The center line indicates the median; the box limits indicate the upper and lower quartiles; the whiskers indicate 1.5 times the IQR; the dots represent the outliers. **g**, Percentage of H3K27me3-broad ACRs nearby genes exhibiting no expression across any cell type was significantly higher than the other ACRs associated with the not expressed genes. Significance testing was performed using one-tailed Fisher’s exact test (alternative = ‘greater’). **h**, Percentage of H3K27me3-broad ACRs nearby genes exhibiting cell-type-specific expression was significantly higher than the other genes not associating with H3K27me3-broad ACRs. Significance testing was performed using one-tailed Fisher’s exact test (alternative = ‘greater’). **i**, The number of H3K27me3-broad-ACR capturing known 53 PREs is determined based on *A. thaliana* scATAC-seq data. The significance test was done by using the one-tailed Binomial test (alternative = ‘greater’). The error bars indicate the mean ± s.d. **j**, Pie charts illustrate the different types of PRE-associated motifs within H3K27me3-broad ACRs. **k**, Percentage of H3K27me3-broad ACRs nearby genes with higher expression in ZS97 than in NIP. Significance testing was performed using one-tailed Fisher’s exact test (alternative = ‘greater’). **l**, A screenshot illustrating a cross-species syntenic ACR likely containing silencer-like CREs shown in Fig. 3h.

To further use the atlas, we investigated the accessibility contexts of ACRs associated with H3K27me3 (ref. ^6^). H3K27me3 is a histone modification associated with facultative heterochromatin established by the POLYCOMB REPRESSIVE COMPLEX 2 (PRC2)^42–44^. Genes silenced by PRC2 and H3K27me3 are important regulators that are only expressed in narrow developmental stages or under specific environmental stimuli, where they often initiate important transcriptional changes^43,45^. This importance makes the identification of key CREs controlling H3K27me3 silencing especially interesting. We examined ACRs near or within H3K27me3 regions and classified them into two groups: H3K27me3-broad, representing H3K27me3-associated ACRs with chromatin accessibility in many cell types, and H3K27me3-cell-type specific, those H3K27me3-associated ACRs with chromatin accessibility in few cell types (Fig. 3a, Extended Data Fig. 3a and Supplementary Table 15). The proportion of H3K27me3-broad and H3K27me3-cell-type-specific ACRs was consistent across organs, based on H3K27me3 profiles collected from the same organs used for scATAC-seq (Extended Data Fig. 3b). H3K27me3-broad ACRs exhibited a depletion of H3K27me3 over the ACR (Fig. 3b), consistent with nucleosome absence in ACRs^46^. By contrast, H3K27me3 depletion was not observed in H3K27me3-cell-type-specific ACRs, with most cells in the bulk chromatin immunoprecipitation followed by sequencing (ChIP-seq) likely containing H3K27me3-modified nucleosomes (Fig. 3b). This is consistent with the H3K27me3-cell-type-specific ACRs potentially acting after the removal of facultative heterochromatin in a specific cell type(s). However, the chromatin accessibility of the H3K27me3-broad ACRs appears to be concurrent with H3K27me3 (Extended Data Fig. 3c), suggesting these ACRs may regulate H3K27me3 maintenance and removal across most cellular contexts.

To assess the transcriptional state of genes near H3K27me3-broad ACRs, we evaluated single-nucleus RNA sequencing (snRNA-seq)/scRNA-seq from *O. sativa* seedling (Supplementary Fig. 7) and root^40^. The results revealed significantly lower expression (*P* = 1.5 × 10^−34^ to 2 × 10^−6^; Wilcoxon signed rank test) for H3K27me3-broad ACRs associated genes across most cell types (Fig. 3c, Extended Data Fig. 3d,e and Supplementary Table 16). Moreover, 58 bulk RNA-seq libraries from *O. sativa* organs^47^ showed the lower (*P* = 1 × 10^−7^ to 0.0133; Wilcoxon signed rank test) expression of genes near H3K27me3-broad ACRs than genes near H3K27me3-absent broad ACRs (Extended Data Fig. 3f). To dissect the roles of H3K27me3-broad ACRs, we identified 2,164 H3K27me3-broad ACRs and measured neighbouring gene expression in *O. sativa* cells (Supplementary Table 17). About 926 (~42.8%) of the H3K27me3-broad ACRs were associated with 838 genes that exhibited no expression across any sampled cell type, which was marginally, but significantly (*P* = 5 × 10^−11^; Fisher’s exact test), higher than the other ACRs associated with the unexpressed genes (Extended Data Fig. 3g and Supplementary Table 17), consistent with these H3K27me3 proximal genes only being expressed under specific conditions. The 1,108 expressed genes associated with H3K27me3-broad ACRs were enriched (*P* < 2 × 10^−16^; Fisher’s exact test) for cell-type specificity compared to genes without H3K27me3 (Extended Data Fig. 3h). In summary, single-cell expression analysis revealed that the genes linked to H3K27me3-broad ACRs exhibited the hallmarks of facultative gene silencing.

We hypothesized that the H3K27me3-broad ACRs would be enriched for PRC2 silencer elements, as their consistent chromatin accessibility provides an avenue to recruit PRC2 to maintain H3K27me3 throughout development. Supporting the presence of silencer CREs with these ACRs, a known silencer CRE ~5.3 kb upstream of *FRIZZY PANICLE* was within a H3K27me3-broad ACR^48^ (Fig. 3d). To exploit the known Polycomb *Arabidopsis thaliana* targets, we used scATAC-seq^20^ and H3K27me3^49^ data from *A. thaliana* roots and annotated H3K27me3-broad ACRs. The *A. thaliana* H3K27me3-broad ACRs significantly (*P* < 2 × 10^−16^; binomial test) captured 53 of the 170 known Polycomb responsive elements compared to a control class of ACRs, supporting their putative silencer function^43^ (Extended Data Fig. 3i). Furthermore, we implemented a de novo motif analysis on the 170 *A. thaliana* elements and identified all reported Polycomb response element (PRE) motifs (CTCC, CCG, G-box, GA-repeat, AC-rich and Telobox)^43^ (Fig. 3e). Using these motifs and our chromatin accessibility data, we predicted putative binding sites in *O. sativa* and observed that five motifs were significantly (*P* = 1 × 10^−178^ to 5 × 10^−6^; binomial test) enriched in the H3K27me3-broad ACRs compared to a genomic control (Fig. 3e). Between 88.0% and 92.7% of H3K27me3-broad ACRs contained at least one PRE motif, with few (0.1–0.2%) ACRs having all six PRE motif types (Extended Data Fig. 3j). We next analysed rice ChIP-seq data of EMBRYONIC FLOWER 2b (EMF2b), a crucial PRC2 component^43,50,51^, revealing a significant overlap between EMF2b peaks and the PRE motifs (Fig. 3f,g). We randomly selected a broad H3K27me3-enriched ACR located upstream of the *LOC_Os08g06320* gene and deleted the GA-repeat and CTCC PRE motifs within this region. These modifications resulted in a significant upregulation of *LOC_Os08g06320* expression (Fig. 3h and Supplementary Fig. 17). In addition, we identified 236 genes with adjacent H3K27me3-broad ACRs that contained PRE motifs and SNPs or indels between ‘Zhenshan 97’ and ‘Nipponbare’ genotypes^52,53^. A total of 133 of these genes had transcriptional changes between the two genotypes (Supplementary Table 18). In ‘Zhenshan 97’, we observed a significant (*P* = 0.015; Wilcoxon signed rank test) increase in gene expression, accompanied by a decrease in H3K27me3 signal (Fig. 3i–k and Extended Data Fig. 3k). The functional validation and genotype comparisons suggest that mutations in PRE motifs might preclude PRC2 targeting, potentially associating with higher expression levels of nearby genes. Beyond PRE motifs, we observed significant enrichment (*P* = 5 × 10^−17^ to 0.0188; hypergeometric test) of motifs from four TF families in H3K27me3-broad ACRs: APETALA2-like (AP2), basic helix–loop–helix, Basic Leucine Zipper and C2H2 zinc-finger (Fig. 3l and Supplementary Table 19). AP2 and C2H2 are known to recruit PRC2^43^, and our motif enrichment supports all these TF families potentially regulating H3K27me3 deposition and facultative heterochromatin formation.

To further investigate whether the H3K27me3-broad ACRs enriched for PRC2 silencer elements are also present in the epigenomic landscapes of other grass species, we analysed H3K27me3-broad ACRs associated with H3K27me3 ChIP-seq data from *Z. mays* and *S. bicolor*^6,20,25^. Our findings revealed a consistent depletion of H3K27me3 at these ACRs and significantly lower gene expression levels for H3K27me3-broad ACR-associated genes across most cell types (Fig. 3b and Extended Data Fig. 3b,e). In addition, the five PRE motifs were also significantly enriched in H3K27me3-broad ACRs compared to a genomic control, with most of these ACRs containing at least one PRE motif (Fig. 3e and Extended Data Fig. 3j). Moreover, a validated H3K27me3-broad ACR in *O. sativa* (Fig. 3h) was conserved with H3K27me3-broad ACRs of *Z. mays* and *S. bicolor* (Extended Data Fig. 3l). These results, consistent with observations from *O. sativa*, suggest that H3K27me3-broad ACRs enriched for PRC2 silencer elements may be a conserved feature across other grass genomes. In summary, these findings suggest that ACRs associated with H3K27me3 and that are broadly accessible across most cell types are possibly enriched for CREs that function as silencers.

To further assess whether DNA methylation exhibits regulatory features similar to those of H3K27me3, we identified broad DNA methylation region (BMR)-associated ACRs with chromatin accessibility in many cell types and compared their motif enrichment profiles. These regions showed highly similar motif enrichment patterns to H3K27me3-broad ACRs yet exhibited minimal genomic overlap with them (Supplementary Figs. 18 and 19), suggesting that the two repressive marks may act through recognition of similar motifs while operating largely in distinct genomic contexts. The limited overlap between BMRs and H3K27me3-marked regions likely reflects mechanistic antagonism, as DNA hypermethylation can hinder PRC2 recruitment, consistent with observations in *Arabidopsis* RdDM mutants where DOMAINS REARRANGED METHYLTRANSFERASE 2 (DRM2)-mediated methylation excludes H3K27me3 deposition^54,55^.

### The landscape of cell-type-specific ACRs across grass species

**Fig. 4:**
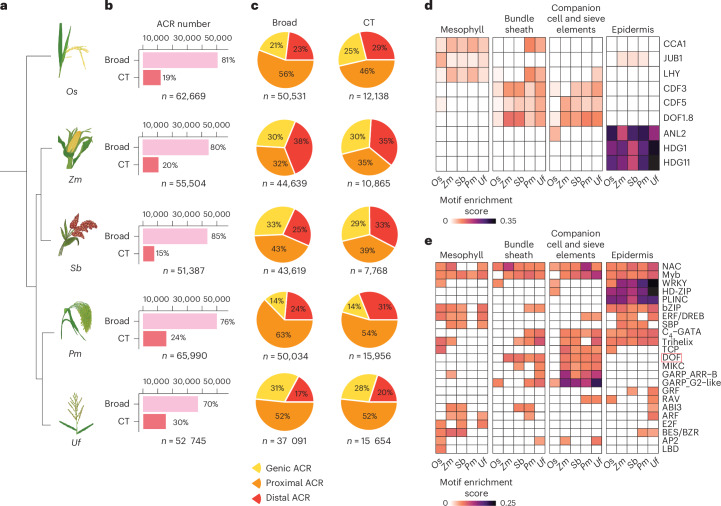
Position and motif enrichment of cell-type-specific ACRs across species. **a**, A phylogenetic tree illustrates five species under examination. *Pm*, *P. miliaceum*; *Uf*, *U. fusca*. **b**, The count of broad and cell-type-specific ACRs. Broad, broadly accessible ACR; CT, cell-type-specific ACR. **c**, Broad and cell-type-specific ACRs were classified into three main groups based on their proximity to the nearest gene: genic ACRs (overlapping a gene), proximal ACRs (located within 2 kb of genes) and distal ACRs (situated more than 2 kb away from genes). *O. sativa*, *P. miliaceum* and *U. fusca* showed a higher percentage of proximal ACRs but a lower percentage of distal ACRs compared to *Z. mays* and *S. bicolor*, likely reflecting differences in intergenic space and overall genome sizes. **d**, A heat map illustrates nine TF motif enrichments, consistent with the known TF dynamics among cell types (CIRCADIAN CLOCK ASSOCIATED 1 (CCA1), LATE ELONGATED HYPOCOTYL (LHY) and JUNGBRUNNEN1 (JUB1): mesophyll^144^; CYCLING DOF FACTOR 3 (CDF3) and CDF5: companion cells^145^; DOF1.8: vascular-related cells^146^; ANTHOCYANINLESS2 (ANL2), HDG1 and HDG11: epidermis^33,147,148^). **e**, A heat map illustrates collapsed TF motif enrichment patterns into super motif families across various species for each cell type. The motif enrichment score cut-off was set to 0.05. The score for each super TF motif family was calculated by averaging the enrichment scores of all the TF motif members within that super family. The DOF TF motif family is highlighted by a red frame. To mitigate the impact of substantial variations in cell numbers across species or cell types, we standardized (down-sampled) the cell counts by randomly selecting 412 cells per cell type per species. This count represents the lowest observed cell count for a given cell type across all species (see ‘Linear-model based motif enrichment analysis’ in the Methods). HD-ZIP, HOMEODOMAIN-LEUCINE ZIPPER; PLINC, PLANT ZINC FINGER.

**Extended Data Fig. 4:**
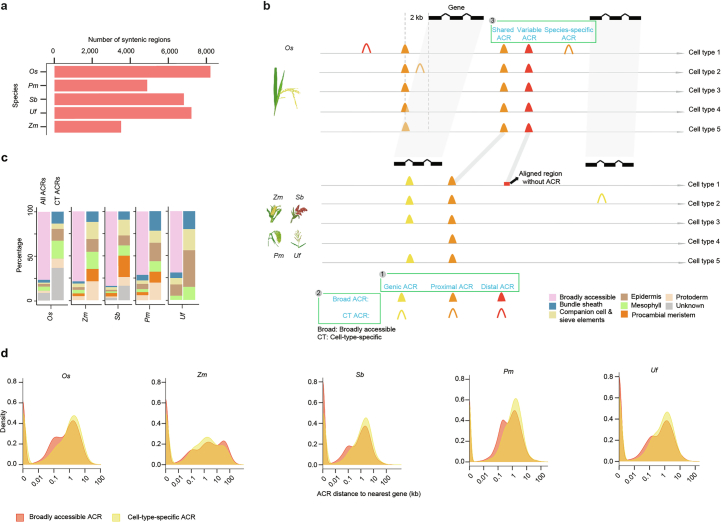
Characterization of ACRs across five grass species. **a**, Number of syntenic regions identified in all five species. Note that *Os* was used as the reference due to the inclusion of the atlas, biasing results to this segment of the tree. **b**, A summary plot displaying the different categories of ACRs: (1) Based on their proximity to the nearest gene, ACRs are divided into three groups: genic ACRs (overlapping a gene), proximal ACRs (within 2 kb of genes), and distal ACRs (more than 2 kb away from genes). (2) According to the statistical approach described in the methods section (Identification of cell-type-specific ACRs), ACRs are classified as either broad ACRs, which are accessible in most cell types (*p* value >= 0.001), or cell-type-specific ACRs, which are accessible in a limited number of cell types (*p* value < 0.001). (3) Based on their syntenic status, ACRs are grouped into shared ACRs (matching sequences accessible in both species), variable ACRs (matching sequences accessible in only one species), and species-specific ACRs (sequences exclusive to a single species). **c**, The distribution of cell-type-specific ACRs across different cell types was characterized by a similar percentage representation. **d**, The distribution of distance of ACRs to their nearest genes in broadly accessible and cell-type-specific ACRs across five species.

This *O. sativa* atlas is a valuable resource for cross-species ACR comparisons, offering an opportunity to explore the evolutionary dynamics of grass ACRs. The *O*. *sativa* ACRs were overlapped with syntenic regions defined by their relationship to four different grass species—*Z. mays*, *S. bicolor*, *P. miliaceum* and *U. fusca*—that have scATAC-seq using combinatorial indexing data from leaves^25^. The analysis revealed that 34% (40,477) of the *O. sativa* ACRs were within 8,199 syntenic regions (~86 Mb of the *O. sativa* genome) shared with at least one of the four examined grass species (Extended Data Fig. 4a and Supplementary Fig. 20). To determine to what degree ACR number, genomic position and cell-type specificity differs among grasses, we compared the composition and distribution of leaf ACRs across the five species (Fig. 4a). The leaf ACRs from *O. sativa* and the other four species were obtained from our previous study^25^. Data quality metrics were thoroughly evaluated in that study, demonstrating that the ACRs from the five species can be reliably compared with minimal influence from technical issues. We calculated the proportion of both broad and cell-type-specific ACRs across all species. We categorized cell-type-specific ACRs as those only accessible in one or two leaf cell types (Extended Data Fig. 4b and Supplementary Table 20). An average of ~53,000 ACRs were identified across the five species, with 15–35% of the ACRs classified as cell-type specific (Fig. 4b, Extended Data Fig. 4c and Supplementary Figs. 21 and 22). Broad and cell-type-specific ACRs were equivalent in their distributions around promoters and distal and genic regions (Fig. 4c and Extended Data Fig. 4d).

**Extended Data Fig. 5:**
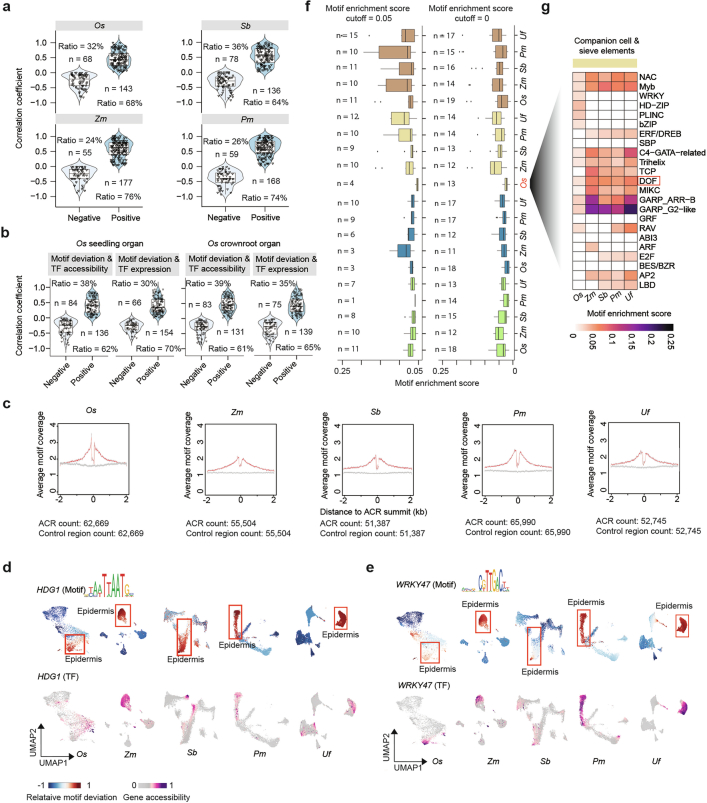
Examination of the chromatin accessibility of motifs enriched in specific cell types across species. **a**, Spearman correlation between chromatin accessibility of TF genes and motif deviation. Note that for *U. fusca* species, the analysis was omitted due to the limited number of cell types (four) available for spearman correlation, making it statistically unfeasible. ‘n’ indicates the number of TF gene–motif pairs analyzed. The center line indicates the median; the box limits indicate the upper and lower quartiles; the whiskers indicate 1.5 times the IQR; the dots represent the outliers. **b**, Spearman correlation of motif deviation to chromatin accessibility and expression of TF genes in *O. sativa* seedling organ. ‘n’ indicates the number of TF gene–motif pairs analyzed. The center line indicates the median; the box limits indicate the upper and lower quartiles; the whiskers indicate 1.5 times the IQR; the dots represent the outliers. **c**, Mean motif coverage centered on ACRs (red line) and control regions (gray line) for each species, with 95% confidence intervals shown as a shaded polygon. **d**, The UMAP panels highlight one TF motif (HDG1) enriched with ACRs in epidermis, where their cognate TFs are known to accumulate. **e**, The UMAP panels highlight one representative TF motif underlying the WRKY family enriched with ACRs in epidermis cells, where their cognate TFs are known to accumulate. **f-g**, Presenting box (**f**) and heatmap (**g**) plots illustrating the collapsed motif enrichment patterns into TF motif families across various species for each cell type. Each dot within the box represents a TF motif family. The center line indicates the median; the box limits indicate the upper and lower quartiles; the whiskers indicate 1.5 times the IQR; the dots represent the outliers. ‘n’ indicates the number of TF motif families. The score for each super TF motif family was calculated by averaging the enrichment scores of all the TF motif members within that super family. The DOF TF motif family was highlighted by a red frame in the heatmap. The reduced motif enrichment cutoff setting reveals additional motif families enriched in companion cells and sieve elements, compared to the higher beta cutoff setting as shown in Fig. 4e. It is worth noting that we still observed lower enrichment scores in *O. sativa* compared to other species.

Previous hypotheses suggested that large-scale regulatory rewiring could play a key role in cell-type environmental adaptation^8,56^. To explore instances where divergent TF activity occurred in the same cell types, we associated TF gene body chromatin accessibility with their cognate TF motifs across different species and cell types. Approximately 64% to 76% of the TFs (211 to 232) examined exhibited a positive correlation between the local chromatin accessibility of their gene body and global enrichment of their cognate binding motifs within ACRs (motif deviation) across all leaf cell types and all species (Extended Data Fig. 5a). The use of TF gene-body chromatin accessibility was supported by an analysis of TF expression and motif deviation in both seedling (Supplementary Fig. 7) and root data^40^, which uncovered a similar positive relationship across cells (Extended Data Fig. 5b). These results suggest a positive relationship between TF gene-body chromatin accessibility/expression and TF activity in the same cell type.

Moreover, the genomic sequences from all ACRs discovered in all species and cell types exhibited enrichment of TF motifs compared to a control set of sequences (Extended Data Fig. 5c). Furthermore, TF motif enrichment analysis revealed known TF-cell-type specificities (Fig. 4d). For example, the HDG1 TF is critical for epidermis and cuticle development^33^, and its motif was enriched in epidermis cells in all five species (Extended Data Fig. 5d). We also observed motif enrichments of WRKYGQK (WRKY), HOMEODOMAIN-LEUCINE ZIPPER and PLANT ZINC FINGER in epidermal cells across all five species examined (Fig. 4e, Extended Data Fig. 5e, Supplementary Fig. 23 and Supplementary Table 21). This result indicates that these TFs may play a conserved role in the development of the grass epidermis. Phloem companion, sieve element cell and bundle sheath cell TFs exhibited similar enrichments across the species (Fig. 4e). However, species-specific motif patterns were also observed, with *O. sativa* being the most different. For example, the DNA-BINDING ONE ZINC FINGER (DOF) TF family motif exhibited higher enrichment scores in the bundle sheath cells of four C_4_ photosynthesizing species (*Z. mays*, *S. bicolor*, *P. miliaceum* and *U. fusca*), as opposed to C_3_ photosynthesizing *O. sativa* (Fig. 4e and Extended Data Fig. 5f,g). The DOF TF family is involved in *A. thaliana* vasculature development^57^ and is important in the transition from C_3_ to C_4_ photosynthesis^23,58–61^. The lack of enrichment of DOF motifs in *O. sativa* bundle sheath cells is therefore an expected biological signal, showing that cell-type-specific motif enrichment in cross-species context can elucidate critical changes in gene regulatory networks likely critical in significant phenological changes.

Taken together, our findings show the power of scATAC-seq data in a comparative framework to explore regulatory evolution, based on both the relationship of ACRs to TF motifs and the relationship between TFs and their corresponding motifs.

### Species-specific evolution of cell-type-specific ACRs

**Fig. 5:**
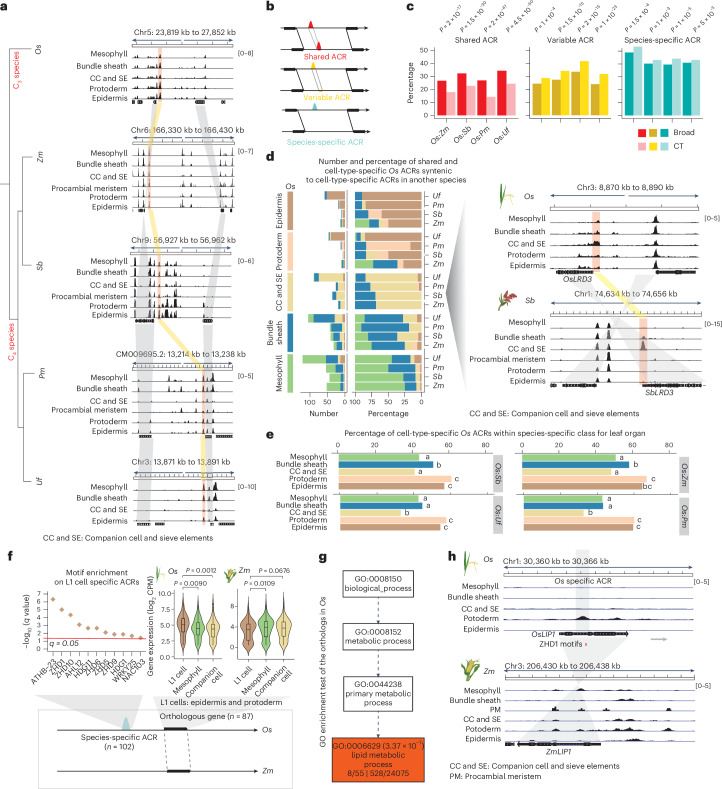
Cell-type-specific ACRs are frequently species-specific. **a**, A screenshot illustrating syntenic regions capturing shared ACRs across five species. The red bars denote syntenic ACRs within regions flanked by corresponding syntenic gene pairs, while the grey colour highlights these syntenic gene pairs. **b**, Three classes depicting variations in ACR conservation between two species. ‘Shared ACRs’, ACRs with matching sequences that are accessible in both species; ‘Variable ACRs’, ACRs with matching sequences but are only accessible in one species; ‘Species-specific ACRs’, ACRs where the sequence is exclusive to a single species. **c**, The percentage of broad and cell-type-specific ACRs underlying three classes shown in **b**. The significance test was done using one-tailed Fisher’s exact test (alternative = ‘greater’). **d**, Left, the number and percentage of *O. sativa* shared ACRs that retain or change cell-type specificity among the other four species. Right, a screenshot of an *O. sativa* phloem-specific ACR that retains phloem specificity in *S. bicolor*. This ACR is situated at the promoter region of *LATERAL ROOT DEVELOPMENT 3* (*LRD3*) which is specifically expressed in companion cell and phloem sieve elements (Supplementary Table 2). The grey shaded region highlights the syntenic gene pair. **e**, The percentage of cell-type-specific ACRs identified across all cell types within species-specific class. The analysis demonstrates an enrichment of cell-type-specific ACRs based on Fisher’s exact test. This test assesses whether cell-type-specific ACRs are more likely to be situated in the ‘species-specific’ class, compared to other cell types. Statistically significant differences (*P* < 0.05 ranging from 0.023 to 0.005) in all pairwise comparisons are denoted by distinct letters, determined using one-tailed Fisher’s exact test with the alternative set to ‘greater’. Bars sharing the same letter indicate that they are not significantly (*P* > 0.05) different from each other. **f**, TF motif enrichment tests were performed in species-specific ACRs neighbouring *O. sativa* orthologue exhibiting higher expression levels in epidermis based on one-tailed binomial test (see ‘Binomial test-based motif enrichment analysis’ in the Methods). Significance testing in violin plot was performed using the *t*-test (alternative = ‘greater’). A total of 87 orthologous genes between *Z. mays* and *O. sativa* were used for the box plot analysis. **g**, GO enrichment test was performed in *O. sativa* orthologous genes based on agriGO^141^. **h**, A screenshot of *LIP1* accessibility in *O. sativa* and *Z. mays* L1 cells which contains an *O. sativa* epidermal specific and species-specific ACR with two ZHD1 motif sites. No corresponding ZHD1 motifs were found in *ZmLIP1*.

**Extended Data Fig. 6:**
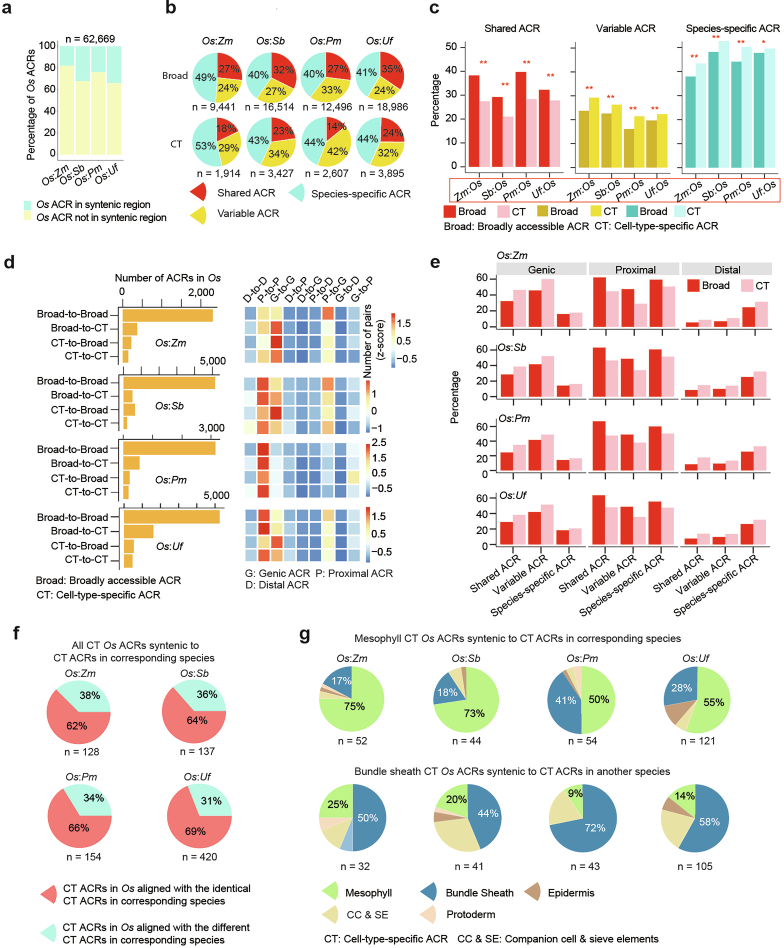
Characterization of ACRs within syntenic regions. **a**, The bar plot illustrates the percentage of *O. sativa* ACRs within syntenic and non-syntenic regions when compared to another species. **b**, The pie charts depict percentages of broadly accessible (Broad) and cell-type-specific (CT) ACRs within three classes of ACR conservation in Fig. 5b. The total number of ACR in *O. sativa* is below the pie charts. **c**, The percentage of broad and cell-type-specific ACRs using *Z. mays*, *S. bicolor*, *P. miliaceum*, or *U. fusca* as the baseline underlying three classes shown in Fig. 5b. The x-axis labels, highlighted by a red frame, are the inverse to x-axis labels in Fig. 5c by using *O. sativa* as a baseline. The significance test was done by using the one-tailed Fisher’s exact test (alternative = ‘greater’). ‘**’ denotes a *p* value < 0.01, and ‘*’ denotes a *p* value < 0.05. The *p* values ranged from 3.2e-43 to 0.025. **d**, Characterization of ACRs under ‘shared ACR’ class and their proximity categorizations. Left, the number of ACRs under the ‘shared ACR’ class, categorized into four combinations based on broadly accessible and cell-type-specific ACRs. Right, a heatmap of ACR categories based on their proximity to genes: genic ACRs overlapping genes, proximal ACRs (within 2 kb of genes), and distal ACRs (more than 2 kb away from the TSS). **e**, The percentage of shared, variable, and species-specific ACR classes in genic, proximal, and distal manners for each species pair. The percentage for each class within genic, proximal, and distal groups collectively sum to 100%. E.g. Genic, proximal and distal broad shared ACRs sum to 100%. **f**, The pie charts illustrate percentage of cell-type-specific *O. sativa* ACRs syntenic to cell-type-specific ACRs in corresponding species. **g**, The pie charts illustrate mesophyll and bundle sheath specific *O. sativa* ACRs syntenic to cell-type-specific ACRs in corresponding species.

To understand how cell-type-specific and broad ACRs changed over evolution, we examined ACRs within syntenic regions among the studied species. To compare ACRs, we devised a synteny-based BLASTN pipeline that allowed us to compare sequences directly (see ‘Identification of syntenic regions’ in the Methods; Fig. 5a, Extended Data Fig. 6a and Supplementary Table 22). Using *O. sativa* ACRs as a reference, we identified three classes of cross-species ACR conservation: (1) ACRs with matching sequences that are accessible in both species (shared ACRs), (2) ACRs with matching sequences but are only accessible in one species (variable ACRs) and (3) ACRs where the sequence is exclusive to a single species (species-specific ACRs; Fig. 5b and Extended Data Fig. 4b). The shared ACR BLASTN hits were often small syntenic sequences, highlighting the large divergence of grass ACRs sequences. However, the majority (92–94%) of these shared BLASTN sequences encoded known TF motifs (Supplementary Fig. 24), indicating that shared ACRs are conserved regulatory regions. By contrast, variable ACRs represent a blend of conserved and divergent regulatory elements, and species-specific ACRs likely indicate novel regulatory loci. We found that, on average, shared ACRs were enriched (*P* = 4.5 × 10^−50^ to 2 × 10^−17^; Fisher’s exact test) for broad ACRs, whereas the variable (*P* = 1 × 10^−23^ to 1 × 10^−4^; Fisher’s exact test) and species-specific (*P* = 1 × 10^−6^ to 5 × 10^−3^; Fisher’s exact test) classes were enriched for cell-type specificity (Fig. 5c and Extended Data Fig. 6b,c). Moreover, we observed that the genomic distribution of shared ACRs was biased towards proximal ACRs (Extended Data Fig. 6d). The cell-type-specific ACRs within the species-specific class were more likely to reside in distal genomic regions compared to the ACRs within the shared and variable classes (Extended Data Fig. 6e).

**Extended Data Fig. 7:**
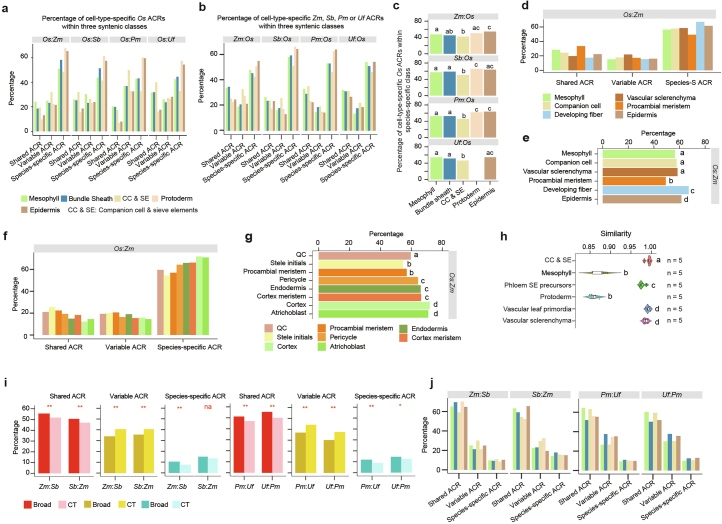
Epidermal cell specific ACRs are less conserved in sequence than other examined cell types. **a**, The proportion of cell-type-specific ACRs identified collectively across all cell types within each species pair under three syntenic classes. The percentage for each cell type within the three classes collectively sum to 100%. **b**, This panel demonstrates a similar meaning to panel **a**, but focuses on using species other than *O. sativa* as the baseline. **c**, The analysis demonstrates an enrichment of cell-type-specific ACRs based on Fisher’s exact test. This test assesses whether cell-type-specific ACRs are more likely to be situated in the ‘species-specific’ class, compared to other cell types. Statistically significant differences (*p* value < 0.05 ranging from 0.009 to 0.012) in all pairwise comparisons are denoted by distinct letters, determined using one-tailed Fisher’s exact test with the alternative set to ‘greater’. Bars sharing the same letter indicate that they are not significantly (*p* value > 0.05) different from each other. *U. fusca*-to-*O. sativa* did not include a bar for protoderm as this cell type has not been identified in *U. fusca* dataset. **d**, The proportion of cell-type-specific ACRs identified collectively across seedling cell types within *O. sativa*-to-*Z. mays* species pair under three syntenic classes (share, variable, and species-specific classes). The percentage for each cell type within the three classes collectively sum to 100%. **e**, This test assesses whether cell-type-specific ACRs are more likely to be situated in the ‘species-specific’ class, compared to other cell types. Statistically significant differences (*p* value < 0.05 ranging from 0.002 to 0.026) in all pairwise comparisons are denoted by distinct letters, determined using a one-tailed Fisher’s exact test with the alternative set to ‘greater’. Bars sharing the same letter indicate that they are not significantly (*p* value > 0.05) different from each other. **f**, The proportion of cell-type-specific ACRs identified collectively across crown root cell types within *O. sativa*-to-*Z. mays* species pair under three syntenic classes (share, variable, and species-specific classes). The percentage for each cell type within the three classes collectively sum to 100%. **g**, This panel demonstrates a similar meaning to panel **e**, but focuses on crown root organ. The *p* values ranged from 0.004 to 0.016. **h**, The MetaNeighbor analysis quantifies transcriptome similarity among cell types in *O. sativa* compared to *Z. mays*. ‘n=5’ denotes five replicates, which were created by randomly dividing the cells from each cell type into five groups, with each group serving as input for the MetaNeighbor analysis. Statistically significant differences (*p* value < 0.05 ranging from 0.008 to 0.021) in all pairwise comparisons are denoted by distinct letters, determined using one-tailed Wilcoxon signed rank test with the alternative set to ‘greater’. The groups sharing the same letter indicate that they are not significantly (*p* value > 0.05) different from each other. The center line indicates the median; the box limits indicate the upper and lower quartiles; the whiskers indicate 1.5 times the IQR; the dots represent the outliers. **i**, The percentage of broad and cell-type-specific ACRs using either *Z. mays* (*Zm*) or *S. bicolor* (*Sb*) as the baseline and using either *P. miliaceum* (*Pm*) or *U. fusca* (*Uf*) as the baseline underlying three classes shown in Fig. 5b. The significance test was done by using the one-tailed Fisher’s exact test (alternative = ‘greater’). ‘**’ denotes a *p* value < 0.01, and ‘*’ denotes a *p* value < 0.05. The *p* values ranged from 1.9e-33 to 0.012. **j**, The percentage of cell-type-specific ACRs identified across all cell types within each species pair split into three classes shown in Fig. 5b. The percentage for each cell type within the three classes collectively sum to 100%.

We further investigated whether the cell-type-specific ACRs were conserved in their cell-type specificity by evolution. By focusing exclusively on ACRs within syntenic regions, pairwise species comparisons revealed that 0.7% (137/19,941; *O. sativa* versus *S. bicolor*), 1.0% (154/15,103; *O. sativa* versus *P. miliaceum*), 1.1% (128/11,355; *O. sativa* versus *Z. mays*) and 1.8% (420/22,881; *O. sativa* versus *U. fusca*) of the syntenic ACRs were shared, retaining the same cell-type specificity in both species (Extended Data Fig. 6b,f), while 19.0% to 24.5% of the syntenic ACRs remained in a broadly accessible manner in these species pairs (Supplementary Table 17). Of these few shared cell-type-specific ACRs, the majority (62–69%) were accessible in the identical cell type in both *O. sativa* and the corresponding species (Fig. 5d and Extended Data Fig. 6f). For example, the promoter ACR associated with *LATERAL ROOT DEVELOPMENT 3*, a gene critical in companion cell and sieve element development^48^, showed sequence conservation between *O. sativa* and *S. bicolor* (Fig. 5d). Interestingly, ACRs which were mesophyll specific in *O. sativa* changed their cell-type specificity to bundle sheath 17–41% of the time, while bundle sheath ACRs changed to mesophyll 9–25% of the time (Fig. 5d and Extended Data Fig. 6g). This result is likely due to the functional divergence associated with the shift from C_3_ (*O. sativa*) to C_4_ (all other species sampled) photosynthesis^23,25^. Of all the classes of cross-species ACR conservation, species-specific ACRs were the most predominant in every cell type (Extended Data Fig. 7a,b). These findings suggest a dynamic and rapid evolution of cell-type-specific ACRs within the examined species. ACRs in L1-derived layer (epidermis and protoderm) exhibited the highest proportion of species-specific ACRs (Fig. 5e and Extended Data Fig. 7c). The high divergence of ACRs in L1-derived cells was also observed in the pairwise comparison between *O. sativa* and *Z. mays* seedling (Extended Data Fig. 7d,e), as well as root cells (Extended Data Fig. 7f,g), suggesting that grass ACRs in L1-derived cells are more divergent compared to internal cell types when comparing *O. sativa* to the other four C_4_ species. To follow up on this observation, we further examined *O. sativa* (Supplementary Fig. 7 and Supplementary Table 23) and *Z. mays* snRNA-seq data^20^ to investigate whether the L1-derived cell types exhibited the most divergent transcriptomes. We found that, among the six examined leaf cell types, protoderm showed the lowest similarity in the gene expression levels between the two species (Extended Data Fig. 7h), which suggests ACR divergence in L1-derived cells likely drives transcriptional change.

Considering that some of these species have diverged from rice 50–70 Ma^62^, we were interested to see whether patterns of rapidly changing L1 regulatory divergence were consistent even within more closely related species pairs such as *Z. mays* and *S. bicolor* derived around 20 Myr^63^. Expectedly, closely related species shared more ACRs across all cell types and broadly specific ACRs (Extended Data Fig. 7i). However, we did not observe more L1-derived species-specific ACRs compared to other cell types in the *Z. mays* and *S. bicolor* comparison, perhaps suggesting it may take more evolutionary time for epidermal CRE differences to accumulate (Extended Data Fig. 7j). Taken together, we observed that ACRs in L1-derived cells are the most divergent compared to other cell types when comparing *O. sativa* to the other four examined C_4_ species.

**Extended Data Fig. 8:**
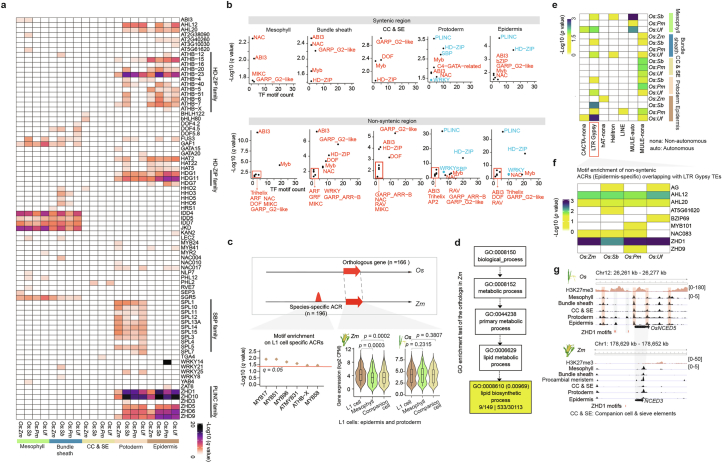
Identification of TF motifs and TEs associating with epidermal cell divergence. **a**, TF motif enrichment tests were performed in species-specific ACRs per species pair using *O. sativa* as the baseline (See Methods: Binomial test-based motif enrichment analysis). The TF motif with a *q* value more than 0.05 was indicated by filling them with a white color. **b**, This panel utilized the same method as panel **a** to conduct TF motif enrichment tests in syntenic and non-syntenic ACRs in the *O. sativa*-to-*Z. mays* species pair (The results from other species pairs were shown in Supplementary Table 24). The ‘-Log10 (*q* value)’ of each TF family was calculated by averaging ‘-Log10 (*q* value)’ across all individual TF motifs within this family. TF family names were marked besides each dark dot, while the PLINC, HD-ZIP, SBP, and WRKY family were highlighted using blue color. The x-axis indicates the number of TF motifs detected to be enriched (*q* value < 0.05) in the relative TF family. **c**, TF motif enrichment tests were performed in species-specific ACRs neighboring *Z. mays* ortholog exhibiting higher expression levels in L1 cell based on Binomial test (See Methods: Binomial test-based motif enrichment analysis). Significance testing in violin plot was performed using the one-tailed t-test (alternative = ‘greater’). The center line indicates the median; the box limits indicate the upper and lower quartiles; the whiskers indicate 1.5 times the IQR; the dots represent the outliers. A total of 166 orthologous genes between *Z. mays* and *O. sativa* were used for the box plot analysis. **d**, GO enrichment test was performed in *Z. mays* orthologous genes. **e**, Enrichment of TE family on ACRs within non-syntenic regions relative to those in syntenic regions. Significance testing was performed using Fisher’s exact test (alternative = ‘greater’). The TE with a *p* value more than 0.05 was indicated by filling them with a white color. **f**, TF motif enrichment tests were performed on the epidermis specific ACRs overlapping with LTR Gypsy TEs based on Binomial test (See Methods: Binomial test-based motif enrichment analysis). The TE with a *p* value more than 0.05 was indicated by filling them with a white color. **g**, A screenshot of *OsNCED5* accessibility in *O. sativa* and *NCED3* accessibility in *Z. mays* L1-derived cells which contains four H3K27me3-broad ACRs and two *O. sativa* epidermal specific and species-specific ACRs with three ZHD1 motif sites.

Returning to using *O. sativa* as the reference, we investigated the TF families underpinning the species-specific ACRs in L1-derived cells. Within all species-specific syntenic ACRs, we observed a predominance of TF motifs for the HOMEODOMAIN-LEUCINE ZIPPER, SQUAMOSA PROMOTER BINDING PROTEIN and PLANT ZINC FINGER families (Extended Data Fig. 8a). Many of these, such as HDG1, ZHD1, ATHB-20, SPL3, SPL4 and SPL5, function in epidermal cell development^15,64–67^. The predominance of these motifs in the species-specific ACR class suggests that although the L1 TF motifs have well-conserved epidermal or protodermal functions (Fig. 4d,e), the ACRs containing these motifs are rapidly evolving and not conserved across grass genomes. Comparing TF-motif enrichment between syntenic and non-syntenic ACRs, we observed the presence of these epidermal motif families in both groups (Extended Data Fig. 8b and Supplementary Table 24), indicating their essential roles in both conserved epidermal cell development and rapid gene-regulatory co-option in species-specific sequences. Some TF-motif families, such as WRKY, were more enriched in non-syntenic ACRs in epidermal cells (Extended Data Fig. 8b). WRKY TF TRANSPARENT TESTA GLABRA2 is a key factor in the regulatory pathways governing leaf epidermal cell differentiation^68^. These results further support that the divergent WRKY TF family targeting may be associated with evolutionary innovation in the epidermal layer.

To look for derived species-specific ACRs associated with the altered expression of surrounding gene orthologues in epidermal cells, we integrated snRNA-seq data from *O. sativa* (Supplementary Fig. 7) with snRNA-seq data from *Z. mays*^20^. We identified 87 orthologous genes, irrespective of synteny, which exhibited higher L1 *O. sativa* expression compared to *Z. mays* (Fig. 5f and Supplementary Table 25). A gene ontology enrichment test for these 87 genes revealed eight genes involved in lipid metabolic process (Fig. 5g), possibly related to cuticle metabolism. Among the eight genes, one was orthologous to *A. thaliana GDSL LIPASE GENE* (*LIP1*), which is epidermis specific^69^. We further identified 102 L1 cell-type-specific ACRs from *O. sativa* that were the closest to the 87 orthologous genes and observed 11 TF motifs enriched (*q* = 3 × 10^−10^ to 5 × 10^−4^; binomial test) in these ACRs (Fig. 5f). These included TF family motifs known for their roles in epidermal cell development such as ZHD1 (ref. ^15^), HDG11 (ref. ^70^), ZHD5 (ref. ^71^), HDG1 (ref. ^33^) and WRKY25 (ref. ^72^). For example, within the *OsLIP1* intron, we identified two ZHD1 motifs within a species-specific ACR that was specifically accessible in L1-derived cells (Fig. 5h). We also flipped this comparison by identifying 166 orthologues with elevated *Z. mays* epidermal expression compared to *O. sativa* (Supplementary Table 25), which associated with 196 L1 cell-type-specific ACRs in *Z. mays*. Within these ACRs, the most enriched (*q* = 0.0129 to 0.0392; binomial test) TF motif was MYELOBLASTOSIS 17 (MYB17; Extended Data Fig. 8c,d). This R2R3 MYB family TF is associated with epidermal cell development, specifically in the regulation of epidermal projections^73^. Furthermore, we hypothesized that some of these novel motifs could be related to *O. sativa* transposable element expansion. We found long terminal repeat retrotransposon-associated ACRs from the *Gypsy* family were enriched (*P* = 0.0006 to 0.0208; Fisher’s exact test) in *O. sativa* epidermal cell-type-specific ACRs (Extended Data Fig. 8e). The ZHD1 motif was enriched within these *Gypsy*-associated ACRs (*P* = 0.0006 to 0.0026; binomial test) (Extended Data Fig. 8f). By linking snRNA-seq to scATAC-seq data, we tied gene-proximal ACR changes to elevated epidermal expression of a small number of conserved orthologues over 50 Myr derived^62^. These ACR changes are associated with variance in species-specific L1-derived layer development, potentially contributing to species differences in environmental adaptation.

As we identified L1-derived cells as being enriched in species-specific ACRs, we sought to examine the changes in H3K27me3 regulation within this tissue. We examined our previously identified 87 *O. sativa*-to-*Z. mays* orthologues to see whether these genes contained H3K27me3. We observed 18 of these 87 genes were close to H3K27me3-broad ACRs (Supplementary Table 26). For example, we identified four H3K27me3-broad ACRs and three ZHD1 motifs linking bulliform cell development^14,15^ within two species-specific ACRs surrounding *O*. *sativa* 9-CIS-EPOXYCAROTENOID DIOXYGENASE 5 (OsNCED5) that were specifically accessible in L1-derived cells (Extended Data Fig. 8g). OsNCED5 TF is known to regulate tolerance to water stress and regulate leaf senescence in *O. sativa*^74^. These results highlight that H3K27me3-mediated silencing may play a critical role in divergent regulation in L1-derived cells.

**Extended Data Fig. 9:**
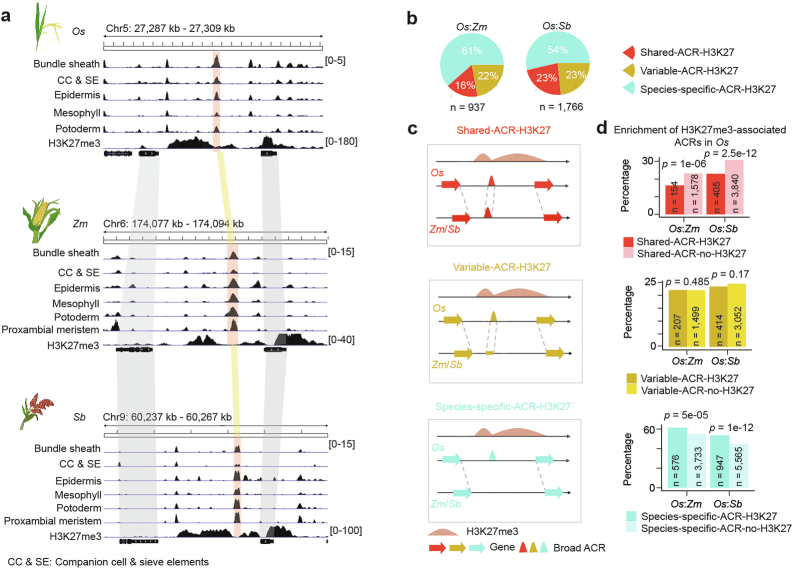
Characterization of H3K27me3-broad ACRs potentially enriched for silencer CREs. **a**, An example of a syntenic region containing H3K27me3-broad ACRs that were conserved across *O. sativa*, *Z. mays*, and *S. bicolor*. **b**, The pie charts depict percentages of H3K27me3-broad ACRs within three classes of ACR conservation in Fig. 5b. **c**, Three categories of *O. sativa* H3K27me3-broad ACRs that are syntenic to regions from another species. **d**, Comparison of ACR percentage underlying three classes corresponding to panel **c** associated with H3K27me3 or not. The *p* values displayed in the bottom bar plot were computed using one-tailed Fisher’s exact test (alternative = ‘greater’).

To investigate the relationship between the H3K27me3-broad ACRs and species divergence, we mirrored our previous syntenic ACR analysis by classifying *O. sativa* H3K27me3-broad ACRs into shared, variable or species-specific groups (Fig. 5b, Extended Data Fig. 9a,b and Supplementary Table 15). Between 54% and 61% of the H3K27me3-broad ACRs were present in the species-specific class, with the H3K27me3-broad ACRs enriched for species specificity compared to H3K27me3-absent-broad ACRs (Extended Data Fig. 9b–d). As these H3K27me3-broad ACRs exhibit hallmarks of PRC2 recruitment, we suspect that altered silencer CREs use context to drive species-specific developmental and environmental responses.

### Conserved non-coding sequences (CNS) are enriched in cell-type-specific ACRs

**Fig. 6:**
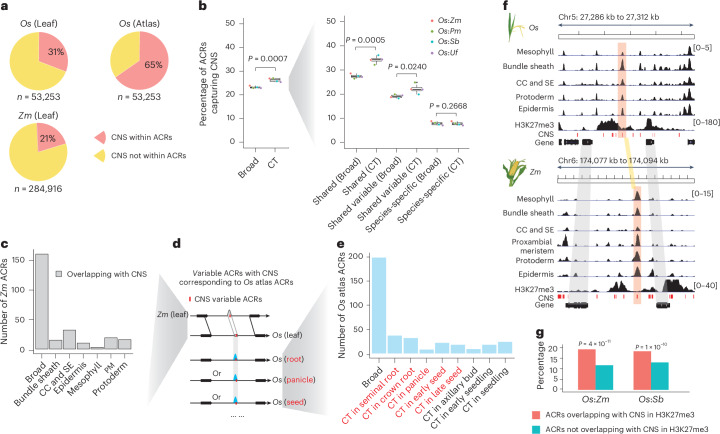
Cell-type-specific ACRs exhibit an enrichment of CNS. **a**, The percentage of CNS overlapping with ACRs. ‘*n*’ indicates the number of CNS. ‘Atlas’ means the ACRs were from the *O. sativa* atlas in Fig. 1b. **b**, Left: the percentage of broad and cell-type-specific ACRs within syntenic regions overlapping with the CNS. Right: this panel presents similar meaning as the left panel but focuses on three classes within syntenic regions shown in Fig. 5b. Significance testing was performed using one-tailed *t*-test (alternative = ‘greater’). The centre line indicates the median; the box limits indicate the upper and lower quartiles; the whiskers indicate 1.5 times the IQR; the dots represent the outliers. Comparisons across four species were treated as four biological replicates for statistical testing. **c**, The count of *Z. mays* variable ACRs accessible in leaf cell types. **d**, A sketch illustrating whether variable ACRs containing CNS in *Z. mays* capture ACRs derived from the *O. sativa* atlas. **e**, The count of *O. sativa* atlas ACRs accessible in non-leaf cell types. **f**, An example of a syntenic block containing *O. sativa*-to-*Z. mays* conserved ACRs within a H3K27me3 region. CNS are highlighted using red colour. **g**, The percentage of ACRs capturing CNS in and outside of H3K27me3 regions. The percentage for each group within H3K27me3 and not within H3K27me3 regions collectively sum to 100%. Significance testing was performed using one-tailed Fisher’s exact test (alternative = ‘greater’).

**Extended Data Fig. 10:**
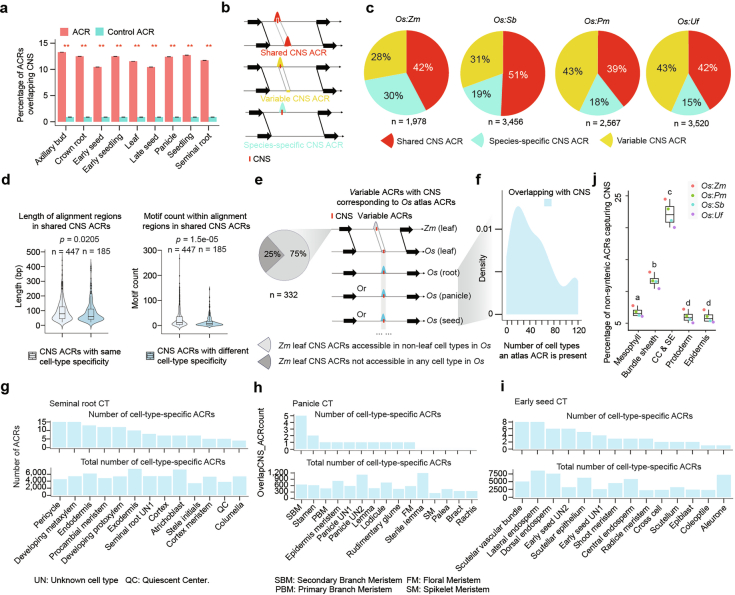
Examination of *O. sativa* atlas ACRs corresponding to variable *Z. mays* ACRs. **a**, Percentage of ACRs overlapping CNS. The significance test was done by using the one-tailed Binomial test (** indicate *p* value < 0.01 ranging from 3e-78 to 2e-56; alternative = ‘greater’). The error bars indicate the mean ± s.d. We generated control sets by simulating sequences with the same length as ACRs 100 times, yielding a mean proportion for the control sets. The binomial test *p* value was calculated by comparing the mean ratio to the observed overlapping ratio of ACRs capturing the CNS. **b**, Three classes depicting variations in ACR conservation between two species. ‘Shared CNS ACRs’: CNS ACRs with matching sequences that are accessible in both species; ‘Variable CNS ACRs’: CNS ACRs with matching sequences, but are only accessible in one species; ‘Species-specific CNS ACRs’: CNS ACRs where the sequence is exclusive to a single species. **c**, The pie charts depict percentages of CNS ACRs within three classes of ACR conservation in panel **b**. The total number of ACR in *O. sativa* is below the pie charts. **d**, Comparison of length of alignment regions and motif count within shared CNS ACRs between with same and different cell-type specificity. Significance tests were performed by the one-tailed Wilcoxon signed rank test (alternative = ‘greater’). ‘n’ indicates the number of CNS ACRs analyzed. The center line indicates the median; the box limits indicate the upper and lower quartiles; the whiskers indicate 1.5 times the IQR; the dots represent the outliers. **e**, A sketch illustrating whether variable ACRs containing CNS in *Z. mays* capture ACRs derived from the *O. sativa* atlas. The left pie chart panel represents the percentage of *Z. mays* leaf CNS ACRs that were accessible in non-leaf cell types in the *O. sativa* atlas. **f**, A density plot illustrating the number of non-leaf *O. sativa* cell types in which an *O. sativa* ACR syntenic to *Z. mays* variable ACRs are accessible. **g-i**, The bar plots represent the count of *O. sativa* atlas cell-type-specific ACRs accessible in non-leaf cell states from seminal root, panicle, and early seed organs. These ACRs were derived from cell-type-specific ACRs from Fig. 6e. **j**, The percentage of non-syntenic ACRs capturing CNS to all non-syntenic ACRs. All pairwise comparisons are statistically significant (*p* value < 0.05 ranging from 6e-10 to 0.034) as indicated by different letters based on one-tailed t-test (alternative = ‘greater’). If two bars have the same letter, then they are equivalent (*p* value > 0.05). The center line indicates the median; the box limits indicate the upper and lower quartiles; the whiskers indicate 1.5 times the IQR; the dots represent the outliers. Comparisons across four species were treated as four biological replicates for statistical testing.

To augment our syntenic ACR BLASTN approach, we intersected our ACRs with published CNS^6,75–77^. Outside of untranslated regions, CNS typically encompass transcriptional regulatory sequences undergoing purifying selection, too critical to be lost during evolution^78^. Using the conservatory database (https://conservatorycns.com/dist/pages/conservatory/about.php)^79^, we extracted 53,253 and 284,916 CNS in *O. sativa* and *Z. mays*, respectively, for analysis. Excluding CNS overlapping with untranslated regions, 30.8% and 21.3% of CNS overlapped with the leaf-derived ACRs in *O. sativa* and *Z. mays*, respectively (Fig. 6a and Extended Data Fig. 10a). Expanding this to include all ACRs in the *O. sativa* atlas, this ratio increased to 65.0% (Fig. 6a), indicating that a significant portion of these CNS likely function in specific cell types and tissues. We further assessed conservation of cell-type specificity associated with CNS. We observed 39% to 51% of total CNS ACRs within the ‘shared CNS ACR’ class (Extended Data Fig. 10b,d), suggesting these ACRs have conserved cellular contexts between *O. sativa* and other species. We then focused on the shared ACRs between *O. sativa* and *Z. mays*, which had overlapping CNS. We found that ACRs with identical cell-type specificity and CNS had significantly (*P* = 0.02057; Wilcoxon signed rank test) longer alignments and more (*P* = 1.5 × 10^−5^; Wilcoxon signed rank test) TF motifs than ACRs with differing cell-type specificity and CNS (Extended Data Fig. 10d). This suggests that these regulatory sequences are likely critical in proper gene expression over deep evolutionary time. Remarkably, within syntenic regions, ACRs containing CNS are more cell-type specific than ACRs without CNS (Fig. 6b). The enrichment of CNS in cell-type-specific ACRs stresses the importance of these sequences as they are likely critical for proper cell-type function over deep evolutionary time.

Although many of the CNS ACRs appeared to maintain their cell-type specificity across our sampled species, we wanted to narrow our analysis to instances where this was not the case. Specifically, we examined the CNS found in *Z. mays* leaf ACRs that had matching sequences in *O. sativa* but were not overlapping a leaf ACR in *O. sativa*. We defined this class as *Z. mays* leaf variable CNS ACRs. Leveraging the *O. sativa* atlas, we examined these CNSs with divergent chromatin accessibility, and 249 (75%) of the *Z. mays* leaf variable CNS ACRs were accessible in non-leaf cell states in *O. sativa* (Fig. 6c–e and Extended Data Fig. 10e), highlighting instances where the leaf availability of these CNS has shifted. Investigating the *O. sativa* CNS ACRs that lost leaf cell-type accessibility and leveraging the atlas, we observed that these ACRs were accessible in many non-leaf cell types, uniformly distributed among the atlas cell annotations (Extended Data Fig. 10f–i). Consistent with our findings that epidermal-specific ACRs tend to have the most species-specific ACRs in syntenic regions (Fig. 5e and Extended Data Fig. 7a–g), L1-derived cells showed a significantly lower ratio of non-syntenic CNS ACRs to non-syntenic CNS-less ACRs compared to other cell types (Extended Data Fig. 10j). This lower ratio shows the frequent loss of epidermal CNS accessibility, further supporting the rapid evolution of epidermal transcriptional regulation.

We noticed a pattern where some CNS within ACRs also overlapped domains of H3K27me3 (ref. ^6^) (Fig. 6f). Upon closer examination, H3K27me3-associated ACRs were significantly enriched for CNS (Fig. 6g) compared to H3K27me3-absent ACRs. This enrichment supports that some of these CNS underpin conserved and critical components of H3K27me3 silencing.

## Discussion

**Fig. 7:**
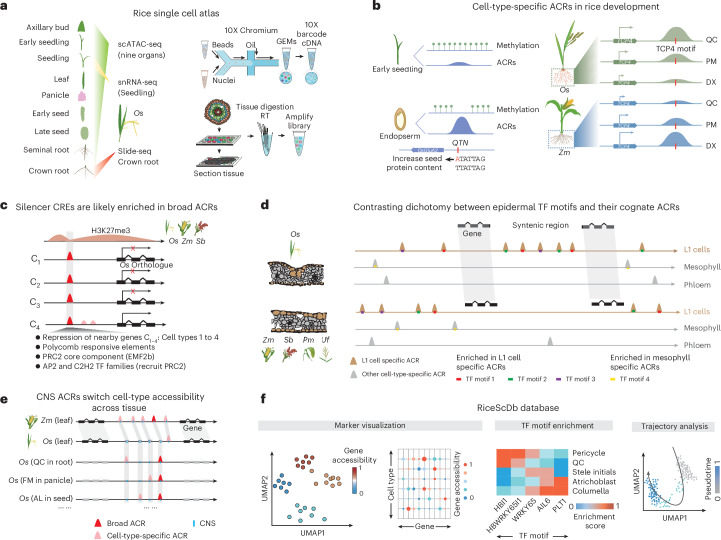
A single-cell rice atlas combines multi-species data to uncover the evolution of CREs. **a**, Overview of the nine organs analysed using scATAC-seq, the seedling organs examined via snRNA-seq and the crown root studied through Slide-seq in rice. **b**, A QTN was identified within an endosperm-specific ACR approximately 1 kb upstream of the *OsGluA2* gene, which is associated with increased seed protein content. TCP4 exhibited reduced motif accessibility during xylem development in *O. sativa*, while TCP4 displayed increased accessibility along the RDX trajectory in *Z. mays*, paralleling scRNA-seq expression patterns during RDX development. **c**, Despite being within facultative heterochromatin, H3K27me3-broad ACRs are accessible in many cell types, providing a physical entry point for PRC2 to bind. Several lines of evidence support that the H3K27me3-broad ACRs contain silencer CREs. Specifically, these ACRs are linked to transcriptionally silent genes, enriched for PRE motifs, enriched for TF family motifs (AP2 and C2H2) reported to recruit PRC2, and enriched for PRC2 subunit (EMF2b) ChIP-seq peaks. **d**, The analysis of leaf cell types across these species revealed an enrichment of cell-type-specific ACRs in species-specific regions. These species-specific ACRs were enriched within L1-derived cells compared to all others examined. **e**, We found an enrichment of CNS in cell-type-specific ACRs. Although some CNS ACRs retained the same cell-type specificity between *O. sativa* and *Z. mays*, these CNS ACRs often switched tissue or cell-type accessibility between grass species. AL, aleurone. **f**, A database, RiceSCBase, was developed to provide access to the rice atlas data, featuring tools for marker visualization, TF motif enrichment and trajectory analysis.

The development of a comprehensive *cis-*regulatory atlas is essential for identifying gene regulatory networks and the loci they act on. Here, we developed a rice *cis*-regulatory atlas including nine major organs at single-cell resolution (Fig. 7a). We used this atlas in multiple ways (Fig. 7b–e), demonstrating its ability to identify candidate causative loci associated with critical agronomic phenotypes, to highlight developmental differences across species, and to identify a set of conserved ACRs, along with candidate CREs that may be critical in Polycomb-mediated gene silencing. This *O. sativa* cell-type ACR atlas allowed the identification of ACRs within H3K27me3 regions that were accessible in most cell types. Several lines of evidence support these H3K27me3-broad ACRs as silencers of linked, transcriptionally repressed genes. Specifically, these putative silencers were enriched for CNS, PREs and related TF motifs, and PRC2 in vivo occupancy^51^ (Fig. 7c). Although additional genome editing and comparison of two genotypes in *O. sativa* reveal the PRE motif mutations within H3K27me3-broad ACRs may preclude PRC2 targeting (Fig. 3h–k), more future genome editings of these putative silencers will reveal more about their role in PRC2 recruitment and gene silencing. We expanded our analysis to integrate scATAC-seq from four grass species, investigating the evolution of cell-type-specific CREs. Similar H3K27me3-broad ACR putative silencers were also present in the epigenomic landscape of *O. sativa*, *Z. mays* and *S. bicolor*, suggesting this is a conserved feature of grass genomes. H3K27me3 silencing is deeply conserved in eukaryotes^80^, and a recent study found that many H3K27me3-marked regions might function as silencer-like regulatory elements in *O. sativa*^45^. We hypothesize that other single-cell comparative genomic investigations will find this pattern of broadly accessible silencers in other angiosperm species with H3K27me3.

Our comparison of *O. sativa* with four other grasses revealed patterns in the evolutionary dynamics of *O. sativa* ACRs, within syntenic and non-syntenic regions, and discovered that the grass L1-derived cells in the leaf exhibit elevated rates of transcriptional regulatory divergence, as well as changes in *cis*-regulatory architecture compared to other cell types over large evolutionary distances (Fig. 7d). Previous studies in cereals such as *Z. mays*, *S. bicolor* and *Setaria viridis* have shown that cell-type-specific marker genes are largely conserved^81^. This contrast suggests that ACRs are undergoing evolutionary changes, but these modifications may have limited impact on the expression of core marker genes, allowing cellular identities to remain stable despite underlying regulatory turnover. In addition, we uncovered a dichotomy in the L1-derived cells: the epidermal TF motifs were the most cell-type specific of all those studied, yet their cognate ACRs exhibited the strongest target divergence among the measured species. This duality highlights tandem conservation of core epidermal motifs and the rapid co-option of novel regulatory regions into these existing regulatory frameworks. This rapid regulatory evolution might relate to the dynamic environmental pressures the leaf epidermis has evolved to withstand and may relate to the higher mutation rate in L1-derived tissues compared to other somatic cell types^13,82^. Although to a lesser extent than the epidermis, this interesting contrast, where the cell-type-restricted TF motifs are conserved and the cell-type-specific chromatin accessibility of cognate ACRs are not, extends to other cell types. This supports a larger pattern of novel ACR evolution that co-opts established cell-type-specific TF networks.

Highlighting the rapid rate of regulatory evolution, ACRs and the CREs within them underpin phenotypic variation within eukaryotes^10,20,83,84^. Despite the link between CREs and phenotypic variation, how these transcriptional regulatory circuits have changed during species divergence is challenging to address. This is partly due to rapid CRE changes occluding pairwise comparison; even closely related plant species share but a fraction of their ACR/CRE complements^6,85^. We used a comparative single-cell epigenomics approach to characterize the evolution of cell-type-specific ACRs and CREs in grasses. We demonstrate that grass cell-type-specific ACRs have changed significantly over 50 Myr^62^, with relatively few (0.7% to 1.8%) cell-type-specific ACRs remaining conserved across the examined plant species (Extended Data Fig. 6b,f). The low ratio highlights the rapid rate at which plant CRE evolution takes place, which has been supported previously^75,85–89^. The repeated whole genome duplications in plant lineages^90,91^, and the functional redundancy they provide, may be the fuel for rapid CRE divergence driving plants’ adaptation to diverse environments^92^.

Integration of the *O. sativa* atlas with CNS revealed ~65% were accessible in at least one cell type (Fig. 6a). We expect that most CNS not captured by an ACR in our study are likely accessible in an unsampled cell type, environmental or developmental condition. This stresses the need for expanded accessible chromatin atlases using more tissues, segments of development and environmental conditions. Most ACRs containing CNS had variable cell-type specificity between species (Fig. 7e), highlighting that deeply conserved grass ACRs readily evolve new spatiotemporal usage. Although less frequent than grass ACRs, around one third of conserved mouse deoxyribonuclease I–hypersensitive sites are altered in human tissue contexts after ~90 Myr of evolution^93^. Thus, although eukaryotic CNS exhibit sequence conservation, their functional context is often altered^94^, with novel spatiotemporal CNS usage appearing prevalent in grass lineages. However, it remains possible that the main CNS function conserved between *O. sativa* and *Z. mays* occurs in non-leaf tissues. Nonetheless, the switching of cell-type accessibility highlights the importance of merging chromatin accessibility data with CNS datasets, as the assumption of conserved CNS sequence equalling conserved CRE function can be insubstantial.

Our rice atlas of cell-type-specific ACRs and this cross-species analysis provides a useful resource to enhance our understanding of regulatory evolution more broadly. Highlighting the rapid rate of regulatory evolution, we believe combining these data with those from more closely related grasses in the future will reveal more nuanced evolutionary dynamics of CRE evolution under different levels of evolutionary time^95^. To further maximize the usability of this atlas, we created a user-friendly database (RiceSCBase; http://ricescbase.com; Fig. 7f). This database offers several features to assist researchers in efficiently exploring chromatin accessibility data for genes and ACRs, analysing cell-type-specific TF motif enrichment and identifying genes, TFs, their motifs and ACRs enriched along cell developmental trajectories. By providing streamlined access to the rice atlas datasets, this database expands opportunities for advancing *cis*-regulatory research within the rice community. This resource, combined with the database, and these observations, will fuel research into identifying key CREs controlling specific genes by demarcating high-confidence targets for genome editing.

## Methods

### Preparation of plant materials

Early seedlings, specifically seedling tissues above ground, were collected 7 and 14 days after sowing. Flag leaf tissue was collected at the V4 stage, characterized by collar formation on leaf 4 of the main stem. Axillary buds were obtained from rice plants grown in the greenhouse at approximately the V8 stage. Rice seminal and crown root tips (bottom 2 cm) were gathered at the same stage as seedling tissues, 14 days after sowing. Panicle tissue was acquired from rice plants grown in the greenhouse. Inflorescence primordia (2–5 mm) were extracted from shoots collected at the R1 growth stage, where panicle branches had formed. Early seeds were collected at approximately 6 days after pollination (DAP), and late seeds at approximately 10 DAP. All tissues were collected between 8 and 9 a.m., and all fresh materials were promptly used for library construction starting at 10 a.m.

### Single-cell ATAC-seq library preparation

In brief, the tissue was finely chopped on ice for approximately 2 min using 600 μl of pre-chilled nuclei isolation buffer (NIB, 10 mM MES–KOH at pH 5.4, 10 mM NaCl, 250 mM sucrose, 0.1 mM spermine, 0.5 mM spermidine, 1 mM DTT, 1% BSA and 0.5% TritonX-100)^96^. After chopping, the entire mixture was passed through a 40 μm cell strainer and then subjected to centrifugation at 500 × *g* for 5 min at 4 °C. The supernatant was carefully decanted, and the pellet was reconstituted in 500 μl of NIB wash buffer, which consisted of 10 mM MES–KOH at pH 5.4, 10 mM NaCl, 250 mM sucrose, 0.1 mM spermine, 0.5 mM spermidine, 1 mM DTT and 1% BSA. The sample was filtered again, this time through a 10 μm cell strainer, and then gently layered onto the surface of 1 ml of a 35% Percoll buffer, prepared by mixing 35% Percoll with 65% NIB wash buffer, in a 1.5 ml centrifuge tube. The nuclei were subjected to centrifugation at 500 × *g* for 10 min at 4 °C. Following centrifugation, the supernatant was carefully removed, and the pellets were resuspended in 10 μl of diluted nuclei buffer (DNB, 10x Genomics catalogue number 2000207). About 5 μl of nuclei were diluted 10 times and stained with DAPI (Sigma catalogue number D9542), and then the nuclei quality and density were evaluated with a haemocytometer under an epifluorescence microscope. The original nuclei were diluted with a DNB buffer to a final concentration of 3,200 nuclei per μl. Finally, 5 μl of nuclei (16,000 nuclei in total) was used as input for scATAC-seq library preparation. scATAC-seq libraries were prepared using the Chromium scATAC v1.1 (Next GEM) kit from 10x Genomics (catalogue number 1000175), following the manufacturer’s instructions. (10x Genomics, CG000209_Chromium_NextGEM_SingleCell_ATAC_ReagentKits_v1.1_UserGuide_RevE). Libraries were sequenced with Illumina NovaSeq 6000 in dual-index mode with 8 and 16 cycles for i7 and i5 indices, respectively.

### Single-nuclei RNA-seq library preparation and data analysis

To minimize RNA degradation and leakage, the tissue was finely chopped on ice for approximately 1 min using 600 μl of pre-chilled NIB containing 0.4 U μl^−1^ RNase inhibitor (Roche, Protector RNase Inhibitor, catalogue RNAINH-RO) and a comparatively low detergent concentration of 0.1% NP-40. Following chopping, the entire mixture was passed through a 40 μm cell strainer and then subjected to centrifugation at 500 × *g* for 5 min at 4 °C. The supernatant was carefully decanted, and the pellet was reconstituted in 500 μl of NIB wash buffer, comprising 10 mM MES–KOH at pH 5.4, 10 mM NaCl, 250 mM sucrose, 0.5% BSA and 0.2 U μl^−1^ RNase inhibitor. The sample was filtered again, this time through a 10 μm cell strainer, and gently layered onto the surface of 1 ml of a 35% Percoll buffer. The Percoll buffer was prepared by mixing 35% Percoll with 65% NIB wash buffer in a 1.5 ml centrifuge tube. The nuclei were then subjected to centrifugation at 500 × *g* for 10 min at 4 °C. After centrifugation, the supernatant was carefully removed, and the pellets were resuspended in 50 μl of NIB wash buffer. Approximately 5 μl of nuclei were diluted tenfold and stained with DAPI (Sigma catalogue number D9542). Subsequently, the nuclei’s quality and density were evaluated with a haemocytometer under a microscope. The original nuclei were further diluted with DNB buffer to achieve a final concentration of 1,000 nuclei per μl. Ultimately, a total of 16,000 nuclei were used as input for snRNA-seq library preparation. For snRNA-seq library preparation, we used the Chromium Next GEM Single Cell 3′GEM Kit v3.1 from 10x Genomics (catalogue number PN-1000123), following the manufacturer’s instructions (10x Genomics, CG000315_ChromiumNextGEMSingleCell3-_GeneExpression_v3.1_DualIndex_RevB). The libraries were subsequently sequenced using the Illumina NovaSeq 6000 in dual-index mode with 10 cycles for the i7 and i5 indices, respectively.

The raw BCL files obtained after sequencing were demultiplexed and converted into FASTQ format using the default settings of the 10x Genomics tool cellranger mkfastq^97^ (v7.0.0). The raw reads were processed with cellranger count^97^ (v7.0.0) using the Japonica rice reference genome^98^ (v7.0). Genes were kept if they were expressed in more than three cells with each cell having a gene expression level of at least 1,000 but no more than 10,000 expressed genes. Cells with over 5% mitochondria or chloroplast counts were filtered out. The expression matrix was normalized to mitigate batch effects based on global-scaling normalization and multicanonical correlation analysis in Seurat^99^ (v4.0). The Scrublet tool^100^ (v0.2) was used to predict doublet cells in this dataset. SCTransform in Seurat (v4.0) was used to normalize the data and identify variable genes. The nearest neighbours were computed using FindNeighbors using 30 PCA dimensions. The clusters were identified using FindClusters with a resolution of 1. The cell types were annotated based on the marker gene list (Supplementary Table 2). To identify genes exhibiting higher expression in a particular cell type than in the others, we used the ‘cpm’ function from edgeR^101^ (v3.38.1), for normalizing the expression matrix. Genes within a specific cell type that displayed more than 1.5-fold change in log_2_ counts per million (CPM) values compared to the average log_2_ CPM across all cell types were determined as specifically expressed genes in that particular cell type.

Transcriptome similarity between cell types of *O. sativa* and *Z. mays* was assessed using the MetaNeighbor package^102^ (v1.0). To statistically compare similarities across different cell types, we randomly divided the cells of each type into five groups. Each group was then used as input for the MetaNeighbor analysis. The area under the receiver operating characteristic curve (auROC) score obtained from MetaNeighbor was used as the similarity score in our analysis.

### Slide-seq library preparation and data analysis

Root tissues from rice seedlings 14 days after sowing were used for the Slide-seq V2 spatial transcriptomics. The tissues were embedded in optimal cutting temperature compound, snap-frozen in a cold 2-methylbutane bath and cryosectioned into 10-µm-thick slices. The spatial transcriptome library was constructed following a published method^103,104^. In brief, the tissue slices were placed on the Slide-seq V2 puck and underwent RNA hybridization and reverse transcription process. After tissue clearing and spatial bead collections, complementary DNA was synthesized and amplified for a total of 14 cycles. The library was constructed using Nextera XT Library Prep Kit (Illumina) following the manufacturer’s instructions.

The reads alignment and quantification were conducted following the Slide-seq pipeline (https://github.com/MacoskoLab/slideseq-tools). The data processing is similar to the procedures applied in the analysis of snRNA-seq but with the resolution set at 0.7 for the FindClusters function in Seurat^99^ (v4.0). The cell types were annotated based on the histology of cross-sectioned roots. The marker genes of each cluster were identified using the Wilcoxon signed rank test in FindAllMarkers.

### RNA in situ hybridization

The rice samples were put into the vacuum tissue processor (HistoCore PEARL, Leica) to fix, dehydrate, clear and embed and were subsequently embedded in paraffin (Paraplast Plus, Leica). The samples were sliced into 8 μm sections with a microtome (Leica RM2265). The cDNAs of the genes were amplified with their specific primer pairs in situ_F/in situ_R and subcloned into the pGEM-T vector (Supplementary Table 27). The pGEM-gene vectors were used as the template to generate sense and antisense RNA probes. Digoxigenin-labelled RNA probes were prepared using a DIG Northern Starter Kit (Roche) according to the manufacturer’s instructions. Slides were observed under bright fields through a microscope (ZEISS) and photographed with an Axiocam 512 colour charge-coupled device camera.

### Raw reads processing of scATAC-seq

Data processing was executed independently for each tissue and/or replicate. Initially, raw BCL files were demultiplexed and converted into FASTQ format, using the default settings of the 10x Genomics tool cellranger-atac make-fastq^105^ (v1.2.0). Using the Japonica rice reference genome^98^ (v7.0), the raw reads underwent processing using cellranger-atac count^105^ (v1.2.0). Subsequent to the initial processing, reads that were uniquely mapped with mapping quality >10 and correctly paired were subjected to further refinement through SAMtools view (v1.7; -f 3 -q 10; ref. ^106^). The Picard tool MarkDuplicates^107^ (v2.16.0) was applied on a per-nucleus basis. A blacklist of regions was devised to exclude potentially spurious reads. The methodology involved the exclusion of regions displaying bias in Tn5 integration from Tn5-treated genomic DNA. Specifically, regions characterized by 1 kb windows with coverage exceeding four times the genome-wide median were eliminated. We further leveraged ChIP-seq input data^6^ to filter out collapsed sequences in the reference using the same criteria. This blacklist also incorporated sequences of low complexity and homopolymeric sequences through RepeatMasker^108^ (v4.1.2). Moreover, nuclear sequences exhibiting homology surpassing 80% to mitochondrial and chloroplast genomes^109^ (BLAST+; v2.11.0) were also included within the blacklist. Furthermore, BAM alignments were converted into BED format, wherein the coordinates of reads mapping to positive and negative strands were subjected to a shift by +4 and −5, respectively. The unique Tn5 integration sites per barcode were finally retained for subsequent analyses.

### Identifying high-quality nuclei

To ensure the acquisition of high-quality nuclei, we harnessed the capabilities of the Socrates package (v4.0) for streamlined processing^20^. To gauge the fraction of reads within peaks, we used MACS2 (ref. ^110^) (v2.2.7.1) with specific parameters (genomesize = 3 × 10^8^, shift = −75, extsize = 150, fdr = 0.05) on the bulk Tn5 integration sites, where genomesize defines the effective genome size used for background estimation, shift and extsize adjust read alignment to better approximate actual fragment centers, and fdr controls the false discovery rate for peak calling. Subsequently, we quantified the number of integration sites per barcode using the callACRs function. Next, we estimated the proximity of Tn5 integration sites to genes, focusing on a 2 kb window surrounding the transcription start site (TSS). This estimation was achieved through the buildMetaData function, which culminated in the creation of a meta file. For further refinement of cell selection, we harnessed the findCells function, implementing several criteria: (1) A minimum read depth of 1,000 Tn5 integration sites was required; (2) the total number of cells was capped at 16,000; (3) the proportion of reads mapping to TSS sites was above 0.2, accompanied by a *z*-score threshold of 3; (4) barcode FRiP scores were required to surpass 0.1, alongside a *z*-score threshold of 2; (5) we filtered out barcodes exhibiting a proportion of reads mapping to mitochondrial and chloroplast genomes that exceeded two standard deviations from the library mean; and (6) we finally used the detectDoublets function to estimate doublet likelihood. These multiple steps ensured the meticulous identification and selection of individual cells, facilitating a robust foundation for subsequent analyses.

### Nuclei clustering

For the nuclei clustering, we leveraged all functions from the Socrates package^20^. We binned the entire genome into consecutive windows, each spanning 500 bp. We then tabulated the count of windows featuring Tn5 insertions per cell. Barcodes falling below one standard deviation from the mean feature counts (with a *z*-score less than 1) were excluded. Moreover, barcodes with fewer than 1,000 features were eliminated. We pruned windows that exhibited accessibility in less than 0.5% or more than 99.5% of all nuclei. To standardize the cleaned matrix, we applied a term frequency-inverse document frequency normalization function. The dimensionality of the normalized matrix underwent reduction through the use of non-negative matrix factorization, facilitated by the R package RcppML^111^ (v0.3.7). We retained 50 column vectors from an uncentred matrix. Subsequently, we selected the top 30,000 windows that displayed the highest residual variance across all cells. To further reduce the dimensionality of the nuclei embedding, we used the uniform manifold approximation and projection (UMAP) technique using umap-learn (*k* = 50, min_dist = 0.1, metrix = ‘euclidean’) in R^112^ (v0.2.8.0). Furthermore, we clustered nuclei using the callClusters function within the Socrates framework^20^. Louvain clustering was applied, with a setting of *k* = 50 nearest neighbours at a resolution of 0.7. This process underwent 100 iterations with 100 random starts. Clusters with an aggregated read depth of less than 1 million and 50 cells were subsequently eliminated. To filter outlying cells in the UMAP embedding, we estimated the mean distance for each nucleus using its 50 nearest neighbours. Nuclei that exceeded three standard deviations from the mean distance were deemed outliers and removed from consideration.

### Estimation of gene accessibility scores

To estimate the gene accessibility, we used a strategy wherein the Tn5 insertion was counted across both the gene body region and a 500 bp extension upstream. Subsequently, we used the SCTransform algorithm from the Seurat package^99^ (v4.0) to normalize the count matrix that was then transformed into a normalized accessibility score, with all positive values scaled to 1.

### Identification of de novo marker genes

For each cell type, we used edgeR^101^ to identify cell-type-specific genes that are differentially accessible. To determine whether genes are accessible in a specific cell type, we compared the genes in the target cell type to those in all other cell types, which served as the reference. We identified genes with a false discovery rate (FDR) < 0.05 and log_2_(fold change) > 0 as candidate genes that are specifically accessible in a particular cell type.

### Cell cycle prediction

The prediction of cell cycle stages per nucleus was executed similarly to annotating cell identities based on the aforementioned enriched scores. In brief, we collected a set of 55 cell-cycle marker genes from a previous study^113^. For every cell-cycle stage, the cumulative gene accessibility score for each nucleus was computed. These resultant scores were subsequently normalized using the mean and standard deviation derived from 1,000 permutations of the 55 random cell-cycle stage genes, with exclusion of the focal stage. *Z*-scores corresponding to each cell-cycle stage were transformed into probabilities using the ‘pnorm’ function in R. Furthermore, the cell-cycle stage displaying the highest probability was designated as the most probable cell stage.

### ACR identification

Upon segregating the comprehensive single-base resolution Tn5 insertion sites BED dataset into distinct subsets aligned with annotated cell types, we executed the MACS2 tool^110^ (v2.2.7.1) for precise peak identification per cell type. We used non-default parameters, specifically the following: --extsize 150, --shift −75 and -nomodel -keep-dup all. To mitigate potential false positives, a permutation strategy was applied, generating an equal number of peaks based on regions that were mappable and non-exonic. This approach encompassed the assessment of Tn5 insertion sites and density within both the original and permuted peak groups. By scrutinizing the permutation outcomes, we devised empirically derived FDR thresholds specific to each cell type. This entailed determining the minimum Tn5 density score within the permutation cohort where the FDR remained <0.05. To further eliminate peaks that exhibited significant overlap with nucleosomes, we applied the NucleoATAC tool^114^ (v0.2.1) to identify potential nucleosome placements. Peaks that featured over 50% alignment with predicted nucleosomes were systematically removed. The average fragment size of reads overlapping with the peaks were calculated, and the peaks with the average fragment size >150 bp were filtered out. Ultimately, the pool of peaks for each cell type was amalgamated and fine-tuned, yielding 500 bp windows that were centred on the summit of ACR coverage.

### Identification of cell-type-specific ACRs

To identify cell-type-specific ACRs across the *O. sativa* atlas, we used a modified entropy metric in our previous study^25^. Briefly, this method calculates the accessibility of ACRs, normalizes these values using CPM and then calculates both the entropy and specificity scores for each ACR in each cell type. We combined this method with a bootstrapping approach^115^. For each cell type and species, a sample of 250 cells was taken 5,000 times with replacement, and the specificity metric was calculated as described above. This specificity metric was then compared against a series of 5,000 null distributions, each consisting of a random shuffle of 250 cells from mixed cell populations. A nonparametric test was then used to compare the median real bootstrap specificity score to the null distributions. ACRs were labelled as cell-type specific if they had a *P* < 0.001. Cell-type-specific ACRs were those with a significant *P* value in a given cell type and found to be specific in one or two cell types for leaf ACRs across five examined species, and in one, two or three cell types for ACRs in the *O. sativa* atlas. This method was used for all species in this study. Due to the number of ACRs and cell types in the *O. sativa* atlas, each tissue was analysed independently, and the results were merged downstream.

### Interactions between distal cell-type-specific ACRs and cell-type-specific genes

Raw Hi-C data from *O. sativa* were collected including five replicates^27^. Low-quality reads were filtered out using Trimmomatic^116^ (v0.40). The clean reads from each replicate were mapped to the Japonica rice reference genome^98^. These mapped reads were then used to obtain normalized contact maps through a two-step approach in the HiC-Pro software^117^ (v3.1.0) in parallel mode. The analysis was run using the default configuration file, with modifications to specify a minimal mapping quality of 10 and enzyme recognition sites of MboI. The valid pairs files obtained from all replicates of the same species were combined using the ‘merge_persample’ step in HiC-Pro for further analysis. Fit-HiC^118^ (v.2.0.7) was used to identify intra-chromatin loops. The input contact maps for Fit-HiC were generated from valid pairs files using the ‘validPairs2FitHiC-fixedSize’ script at a 5 kb resolution. Fragments and bias files were generated using ‘creatFitHiCFragments-fixedsize’ and ‘HiCKRy’ scripts separately. Significant intra-chromosomal interactions were identified by running FitHiC at a 5 kb resolution. These significant interactions were further merged and filtered using ‘merge-filter.sh’ script at a 5 kb resolution and with a FDR of 0.05. Each bin of the significant intra-chromatin loops was intersected with distal cell-type-specific ACRs using the ‘intersect’ function in bedtools^119^ (v2.18). The bins associated with those intersecting the distal cell-type-specific ACRs were further examined to determine whether any intersected with promoter regions of cell-type-specific genes, defined as regions 2 kb upstream of the genes.

### Aligning pseudotime trajectories between rice and maize

Motif deviation scores were computed using ChromVAR^120^ (v1.18.0) for both rice and maize cells originated from the trajectories of RDX and cortex development. To refine these scores, a diffusion approach based on the MAGIC algorithm^121^ was used. We further used cellAlign tool^122^ (v0.1.0), a technique that standardized the imputed deviation scores across a predefined set of points (*n* = 200) distributed evenly along the trajectories of rice and maize. This normalization strategy aimed to mitigate technical biases inherent in the data. Specifically, this strategy calculates Euclidean distances between aligned pseudotime trajectories, and then normalizes the alignment-based distance by dividing it by the square root of the number of genes used. For each motif pair shared between rice and maize, a comprehensive global alignment procedure ensured to align the imputed deviation scores across pseudotime for both *O. sativa* and *Z. mays*. Subsequently, we calculated the normalized distance between the two species using the cellAlign tool. Motif clustering based on these distance scores, using *k*-means, yielded two distinct groups: group 1, characterized by relatively higher distances, and group 2. A linear regression framework was subsequently introduced, using distance scores and pseudotime as predictive variables for motifs within these two groups. In instances where motif pairs within either group exhibited positive or negative coefficients, we classified them as ‘shiftEarlyrice’ or ‘shiftEarlymaize’. Motif pairs in Group 1 were designated as ‘conserved’ if their coefficients bore identical positive or negative attributes. To enhance the robustness of our findings, *P* values acquired from the linear regression analysis underwent adjustment using the Benjamini–Hochberg procedure, effectively addressing multiple comparisons. If the corrected *P* value exceeded 0.05, the motif pairs were categorized as ‘unknown’.

### Correlation between chromatin accessibility of TF genes and motif deviation

We sourced rice and *A. thaliana* TFs from PlantTFDB^123^ (v4.0) database. To identify rice orthologues of *A. thaliana* TFs, we used BLAST^109^ (BLAST+; v2.11.0) by using protein fasta alignments with an *e*-value threshold of 1 × 10^−5^ used for significance. Alignments were restricted to fasta sequences categorized as TFs from either species. To further refine the putative orthologues, we applied filters based on functional similarity to *A. thaliana* TFs. Alignments with less than 15% identity were excluded, along with rice TFs associated with distinct families. From the remaining candidates, we selected the orthologues showing the highest Pearson correlation coefficient concerning the motif deviation scores. Motif deviation scores of specific TF motifs within nuclei were computed via chromVAR^120^ (v1.18.0).

### Linear-model-based motif enrichment analysis

We used the FIMO tool from the MEME suite^124^ (v5.1.1) with a significance threshold of *P* < 10^−5^ to predict motif locations. The motif frequency matrix used was sourced from the JASPAR plants motif database^125^ (v9). Subsequently, we constructed a binarized peak-by-motif matrix and a motif-by-cell count matrix. This involved multiplying the peak-by-cell matrix with the peak-by-motif matrix. To address potential overrepresentation and computational efficiency, down-sampling was implemented. Specifically, we standardized the cell count by randomly selecting 412 cells per cell type per species. This count represents the lowest observed cell count for a given cell type across all species. For each cell type annotation, total motif counts were predicted through negative binomial regression. This involved two input variables: an indicator column for the annotation, serving as the primary variable of interest, and a covariate representing the logarithm of the total number of nonzero entries in the input peak matrix for each cell. The regression provided coefficients for the annotation indicator column and an intercept. These coefficients facilitated the estimation of fold changes in motif counts for the annotation of interest in relation to cells from all other annotations. This iterative process was conducted for all motifs across all cell types. The obtained *P* values were adjusted using the Benjamini–Hochberg procedure to account for multiple comparisons. Finally, enriched motifs were identified by applying a dual filter criterion: corrected *P* < 0.01, fold-change of the top enriched TF motif in cell-type-specific peaks for all cell types should be over 1, and beta (motif enrichment score) >0.05 or beta > 0.

### Binomial-test-based motif enrichment analysis

To assess the enrichment of motifs in a target set of ACRs, we performed analysis for each specific motif. We randomly selected an equivalent number of ACRs as found in the target set, repeating this process 100 times. The randomly selected ACR set did not overlap with the actual target set of ACRs. Following this, we computed the average ratio of ACRs capturing the motif within the null distribution.

Subsequently, we executed an exact binomial test^126^, wherein we set this ratio as the hypothesized probability of success. The number of ACRs overlapping the motif in the target set was considered the number of successes, while the total number of ACRs in the target set represented the number of trials. The alternative hypothesis was specified as ‘greater’. This meticulous approach allowed us to robustly evaluate and identify significant motif enrichments within the target set of ACRs.

### Construction of control sets for enrichment tests

To check whether non-coding sequences (non-CDS) QTNs could be significantly captured by ACRs, we generated control sets by simulating sequences with the same length as non-CDS QTNs 100 times, yielding a mean proportion for the control sets. The binomial test *P* value was calculated by comparing the mean ratio to the observed overlapping ratio of non-CDS QTNs captured by ACRs.

To check whether ACRs could significantly capture CNS, we generated control sets by simulating sequences with the same length as ACRs 100 times, yielding a mean proportion for the control sets. The binomial test *P* value was calculated by comparing the mean ratio to the observed overlapping ratio of ACRs capturing the CNS.

To perform comparative analysis of expression levels and chromatin accessibility of genes surrounding broad ACRs under and outside of H3K27me3 peaks, we sampled the same number of ACRs per cell type regarding the broad ACRs not under H3K27me3 peaks. This step is to make sure that their nearby gene chromatin accessibility exhibited similar values compared to the broad ACRs under the H3K27me3 peaks.

To check whether the H3K27me3-broad-ACRs could significantly capture the known PREs and capture the EMF2b ChIP-seq peaks, we generated control sets by randomly selecting not-H3K27me3-broad-ACR instances 100 times, yielding a mean number value for the control sets. The binomial test *P* value was calculated by comparing the mean ratio to the observed number of H3K27me3-broad ACRs overlapped with the PREs.

To test whether H3K27me3-broad ACRs in *O. sativa*, *Z. mays* and *S. bicolor* significantly capture six known motifs, we generated control sets by simulating sequences with the same length as ACRs 100 times, yielding a mean proportion for the control sets. The binomial test *P* value was calculated by comparing the mean ratio to the observed overlapping ratio of H3K27me3-broad ACRs capturing the motifs. The same process was conducted to examine whether the EMF2b peaks significantly capture the six motifs.

### Identification of H3K27me3-broad ACRs

We first implemented a series of cut-offs to determine whether the peak is accessible for a specific cell type. For each cell type, we first normalized the read coverage depth obtained from the MACS2 tool divided by total count of reads and ensured that the maximum of normalized coverage within the peak exceeded a predefined threshold set at 2. In addition, we calculated Tn5 integration sites per peak, filtering out peaks with fewer than 20 integration sites. Subsequently, we constructed a peak-by-cell-type matrix with Tn5 integration site counts. This matrix underwent normalization using the ‘cpm’ function wrapped in edgeR^101^ (v3.38.1) and ‘normalize.quantiles’ function wrapped within preprocessCore^127^ (v1.57.1) in the R programming environment. To further refine our selection, a threshold of 2 was set for the counts per million value per peak per cell type. Peaks that satisfied these distinct cut-off criteria were deemed accessible in the designated cell types. For analysing cell types with fewer than 10 samples (*n* < 10), we established criteria where H3K27me3-broad ACRs must be accessible in at least *n* − 1 cell types, while cell-type-specific ACRs should be accessible in fewer than 3 of the examined cell types. For analyses involving more than 10 cell types, we adjusted the criteria: H3K27me3-broad ACRs must be accessible in at least *n* − 2 cell types, and cell-type-specific ACRs should be accessible in fewer than 4 of the examined cell types.

### De novo motif analysis

To identify position weight matrix of six known motifs within 170 *A. thaliana* PREs^43^, we used the streme function with default settings from the MEME suite^124^ (v5.1.1). The control sequences were built up to match each PRE sequence by excluding exons, PREs and unmappable regions, and they possess a similar GC content (<5% average difference) and same sequence length compared to the positive set.

### Identification of syntenic regions

Identification of syntenic gene blocks was done using the GENESPACE^128^ (v1.4). In brief, to establish orthologous relationships between ACR sequences, ACRs in the *O. sativa* genome were extended to incorporate the two closest gene models for a ‘query block’ as GENESPACE only draws relationships between protein coding sequences. Then the GENESPACE function ‘query_hits’ was used with the argument ‘synOnly = TRUE’ to retrieve syntenic blocks. The resulting syntenic hits were further filtered to allow only a one-to-one relationship between *O. sativa* and the corresponding species. The corresponding syntenic blocks were then named and numbered, and both the genes and genomic coordinates were recorded.

To further identify corresponding ACRs within these blocks, we set up a BLASTN pipeline^129^ (v2.13.0). For each comparison of species, using *O. sativa* as the reference, the underlying nucleotide sequences of the syntenic regions were extracted using Seqkit and used as the blast reference database^130^ (v2.5.1). The sequences underlying the ACRs within the same syntenic region in a different species were then used as the query. The blast was done using the following parameters to allow for alignment of shorter sequences ‘-task blastn-short -evalue 1e-3 -max_target_seqs 4-word_size 7 -gapopen 5 -gapextend 2 -penalty -1 -reward 1 -outfmt 6’. This procedure was run for each syntenic region separately for all species comparisons. The resulting BLASTN files were combined and then filtered using a custom script. Alignments were only considered valid if the *e* value passed a stringent threshold of 1 × 10^−3^, and the alignment was greater than 20 nucleotides with the majority of the shared ACRs (92% to 94%) containing the alignment regions including TF motif binding sites (Supplementary Fig. 24). The resulting filtered BLAST files and the BED files generated from these BLAST files allowed us to draw our relationships between ACRs in the corresponding syntenic space. For all analyses, ACRs were considered to have conserved cell-type specificity if these ACRs would be aligned by BLAST and had the same cell type as assigned by the above method.

### Estimation of conservation scores

Conservation scores were predicted using PhyloP^131^ (v1.0), where values are scaled between 0 and 1, with 1 being highly conserved and 0 being non-conserved. Phylogenies to train PhyloP were generated using PhyloFit^132^ (v1.0), and neutral and conserved sequences were identified using the whole genome aligner progressive cactus.

### ChIP-seq analysis

The clean reads of EMF2b were downloaded from a previous study^51^. The reads were mapped to the rice reference genome^98^ (v7.0) using bowtie2^133^ (v2.5.2) with the following parameters: ‘--very-sensitive --end-to-end’. Reads with mapping quality (MAPQ) > 5 were used for the subsequent analysis. Aligned reads were sorted, and duplicated reads were removed using SAMtools^106^ (v1.7). Peak calling was performed using epic2^134^ with the following parameters: ‘-fdr 0.01 --bin-size 150 --gaps-allowed 1’. The peak ‘BED’ and ‘BIGWIG’ files of H3K27me3 ChIP-seq data for leaf, root, and panicle rice organs were downloaded from RiceENCODE^135^ (http://glab.hzau.edu.cn/RiceENCODE/).

### DNA methylation data analysis and broad methylation region identification

Whole-genome bisulfite sequencing datasets of seedling leaves from rice and maize were retrieved from the National Center for Biotechnology Information (NCBI) Sequence Read Archive (accession numbers: SRR107636641 (ref. ^52^), SRR124639722 (ref. ^136^)). Raw reads were preprocessed to remove low-quality sequences and adapter contamination using Trimmomatic v0.363 (ref. ^116^), applying the following settings: ‘TruSeq3-PE.fa:2:30:10 LEADING:20 TRAILING:20 SLIDINGWINDOW:4:20 MINLEN:50’. After quality filtering, genome indexing was performed using the bismark_genome_preparation utility included in Bismark v0.22.34 (ref. ^137^), based on the corresponding reference genome. Subsequently, the bisulfite-treated reads were aligned to the indexed reference using Bismark v0.22.3 in combination with Bowtie2 v2.3.25 (ref. ^133^), with alignment parameters set to ‘–N 1 –L 20’. PCR duplicates were eliminated using deduplicate_bismark with default options, ensuring that only uniquely mapped reads were preserved for downstream methylation analysis. Detection of methylated cytosines was carried out with the bismark_methylation_extractor, using the flags ‘-no_overlap -CX_context’. To analyse methylation profiles across the genome, a sliding window approach was implemented using the make windows function from bedtools^119^, with a bin size of 500 bp. BMRs were defined as regions where the methylation level exceeds the average methylation level in at least three consecutive windows. Further analyses, including methylation pattern profiling around ACRs and motif enrichment analysis, were performed in parallel using the same methods as applied to H3K27me3 profiling.

### Genetic variants calling in ZS97 genotype in *O. sativa*

We obtained raw sequencing data for the ZS97 genotype of *O. sativa* from a published study^82^. After quality filtering the raw reads using fastp^138^ (v0.23.4), we aligned them to the Japonica *O. sativa* reference genome^98^ using the BWA-MEM algorithm^139^ (v0.7.8). We then used Picard tool MarkDuplicates^107^ (v2.16.0) to remove PCR duplicates. The final genetic variants file was generated using the HaplotypeCaller function in GATK^140^ (GATK 4.2.3.0).

### Gene ontology (GO) enrichment test

The GO enrichment tests were performed based on the AgriGO^141^ (v2) by setting the chi-square statistical test and multi-test adjustment method is Hochberg (FDR).

### Transgenic analysis

The generation of transgenic plants was carried out by transforming vectors into *Agrobacterium tumefaciens* EHA105, as previously reported^142^. EHA105 strains containing the vectors were used to transform rice plants of *O. sativa* spp. japonica background. Deletion plants were generated by using clustered regularly interspaced short palindromic repeats and CRISPR-associated protein 9 (CRISPR–Cas9). Rice plants were grown in a greenhouse at Sichuan Agricultural University (Chengdu, Sichuan, China) under a 25 °C/22 °C (day/night) temperature regime. PCR was used to amplify the Cas9-edited target sequences (Supplementary Table 28) to confirm the deletions, and quantitative real-time PCR (qRT-PCR) was used to analyse gene expression in the deletion plants.

### qRT-PCR analysis

Fresh leaf samples from rice mutant and wild-type plants were collected and immediately preserved at −80 °C. Total RNA was extracted using a high-purity RNA extraction kit (Qinshi Biotechnology). The synthesis of cDNA and qRT-PCR were performed using a one-step cDNA synthesis premix (Tiangen Biotechnology) and a qPCR-specific premix (Novogene Biotechnology), respectively. Actin was used as the internal reference gene, and quantitative data were generated using a Bio-Rad CFX96 Touch system (Bio-Rad). Relative gene expression levels were calculated using the 2-ΔΔCT method. All experiments were conducted with three technical replicates. The primer sequence information used in this study is detailed in Supplementary Table 28.

### Additional resources

Cell-type resolved data can be viewed through our public Plant Epigenome JBrowse Genome Browser^143^ at http://epigenome.genetics.uga.edu/PlantEpigenome/index.html.

### Reporting summary

Further information on research design is available in the Nature Portfolio Reporting Summary linked to this article.

## Supplementary information

Supplementary Information Supplementary Figs. 1–24 and Section 1.

Reporting Summary

Supplementary Tables Supplementary Tables 1–28.

## Data Availability

scATAC-seq data encompassing 18 libraries from nine organs are available via NCBI (PRJNA1007577/GSE252040; https://dataview.ncbi.nlm.nih.gov/object/PRJNA1007577?reviewer=kgarq48dii11vomg44kgr1jq66; PRJNA1052039; https://dataview.ncbi.nlm.nih.gov/object/PRJNA1052039?reviewer=flhu9sl84o5m999r1ph8tlmmbg).

## Code availability

The code used for the analyses throughout the manuscript is available via GitHub at https://github.com/yanhaidong1/scATAC-seq_cross_species.
