# Supplementary material for: A single-cell rice atlas integrates multi-species data to reveal cis-regulatory evolution
Source: Nat Plants. 2025 Sep 17;11(10):2050–71. doi: 10.1038/s41477-025-02106-6 (PMC12537502; doi:10.1038/s41477-025-02106-6)
Supplement: Supplementary file 1 — Supplementary Figs. 1–24 and Section 1. [file 41477_2025_2106_MOESM1_ESM.pdf]

nature plants

Supplementary information https://doi.org/10.1038/s41477-025-02106-6

A single-cell rice atlas integrates multi-species data to reveal *cis*-regulatory evolution

In the format provided by the authors and unedited

1. Cell-type annotation and validation

Upon completing the initial annotation process, which was based on a curated list of marker genes, we further expanded our marker repertoire by incorporating markers collected from published bulk RNA-seq data encompassing a diverse array of cell types, which were acquired *via* laser capture dissection, as well as a published scRNA-seq data encompassing panicle related cell types[1]. In brief, we collected the markers from several studies, including three distinct cell types within rice leaves[2] and ten cell types across rice seed organs[3,4]. From these sources, we selected the top 100 variably expressed markers for each cell type and employed them to compute cell identity enrichment scores. We undertook a comprehensive assessment of markers linked to the different cell types. Subsequently, we randomly drew 100 markers from this pool and repeated this procedure 1,000 times to construct a null distribution based on marker chromatin accessibility scores. For each target cell type marker, we compared their accessibility scores to this null distribution. This facilitated the derivation of an enrichment score per cell, delineating the marker's significance for each representative cell type. We next employed a MAGIC algorithm[5] to refine these enrichment scores. These scores were then mapped onto a UMAP plot, enhancing the cell identity annotation.

Furthermore, we undertook validation of our single-cell chromatin accessibility atlas through integration with published scRNA-seq from rice root tissue. This validation was achieved through two distinct approaches (Supplementary Fig. 9). In the first approach, we leveraged the marked enrichment technique, adapting the above mentioned methodology with the incorporation of the top 20 markers derived from marker identification using the 'FindMarkers' function in Seurat[6] (v4.0). Following the acquisition of a smoothed score for each cell type, individual cells were annotated to specific cell types based on the largest enrichment score within that cell type. A threshold was further set, requiring the maximum score to exceed 0.5 for confident labeling; otherwise, the cell was labeled as 'Unknown'. The second approach entailed employing a k-nearest neighbor (knn) strategy. This strategy commenced with the normalization of scRNA-seq datasets, mirroring the process applied to scATAC-seq datasets. The top 3,000 most variable genes within the scATAC-seq dataset were then identified using the Seurat function 'FindVariableFeatures', subsequently filtering to include only genes common to both datasets. By treating the scRNA-seq cells as a reference, a dimension reduction process was

conducted to generate a loading matrix, which was then utilized to project the scATAC-seq cells onto the scRNA-seq cell embedding. The integration of these two datasets was achieved through the Harmony algorithm[7] (v0.1.0). Within the dual embeddings, the 20 nearest neighbors of each scATAC-seq cell in the scRNA-seq dataset were computed. The most frequent label among these RNA neighbors (> 10 cells) was subsequently assigned as the label for each scATAC-seq cell or designated as NA if no label meeting this threshold was identified.

The annotation of cell types in scATAC-seq datasets encompasses various approaches. 1) We evaluated differential chromatin accessibility among clusters within each organ, using a curated list of 315 marker genes (Supplementary Table 2; Supplementary Fig. 3 and 4). The annotation of cell types within leaf tissue was collected from our previous published study[8]. 2) In an effort to enhance the annotation, we incorporated markers from published scRNA-seq and bulk RNA-seq data of diverse cell types obtained through laser capture dissections (Supplementary Fig. 5). 3) For the clusters, particularly those associated with vasculature-related cell types, which are difficult to assign using the aforementioned approaches, we used sub-clustering techniques to refine their cell identities (Supplementary Fig. 6). While sub-clustering enhances the clarity of most cell identities, certain cell types remain challenging to annotate. For instance, within early seedling organ, subcluster 14_2 comprises cells categorized as 'Unknown' due to the notable chromatin accessibility of markers associated with phloem parenchyma, companion cell, xylem parenchyma, and vascular sclerenchyma. Similarly, subcluster 2_1 is designated as 'Unknown' because no marker exhibited notable gene chromatin within this cluster. Furthermore, subcluster 2_2 is also labeled as 'Unknown', as evidenced by the relative high chromatin accessibility of *xcp1*, a marker for developing xylem, within this subcluster, along with notable chromatin accessibility of *OsSWEET11*, indicative of phloem parenchyma (Supplementary Fig. 6). 4) To further validate our single-cell chromatin accessibility atlas against a recent scRNA-seq dataset from rice root tissue (Supplementary Fig. 7 and 8; Supplementary Table 3), we used two approaches: a K-nearest neighbor (KNN)-based strategy and marker enrichment labeling for label transferring (Supplementary Fig. 9). The results showed a high degree of concordance between the assigned cell types in both approaches. 5) Furthermore, we conducted novel marker gene set enrichment associated with Gene Ontology (GO) terms, which further confirmed the anticipated functions of recognized cell types. For instance, it highlighted processes like lipid biosynthetic activity within epidermis cells and photosynthesis within mesophyll cells

(Supplementary Table 4). 6) In addition, we predicted the cell cycle stage for each cell and observed that meristem-related cells were predominantly in the G2/M and S phases, whereas mature cells were primarily in the G1 phase (Supplementary Fig. 10), which corresponds to the meristem cells undergoing mitotic cell division to produce more cells[9,10]. 7) Moreover, we examined the correlations of cell types across different organs using the top 500 variant genes across clusters and found consistent patterns among related cell states across different organs (Supplementary Fig. 11). 8-9) We conducted RNA *in situ* analyses and slide-seq spatial transcriptomics for a subset of differentially accessible genes (Supplementary Table 5), that lacked prior indications of cell-type specificity. In each case examined, the *in situ* and spatial expression patterns aligned with the anticipated localization derived from the gene accessibility predictions (Supplementary Fig. 12-14; Supplementary Table 6 and 7). Overall, these analyses would significantly increase the confidence in the cell-type annotation of the cis-regulatory atlas.

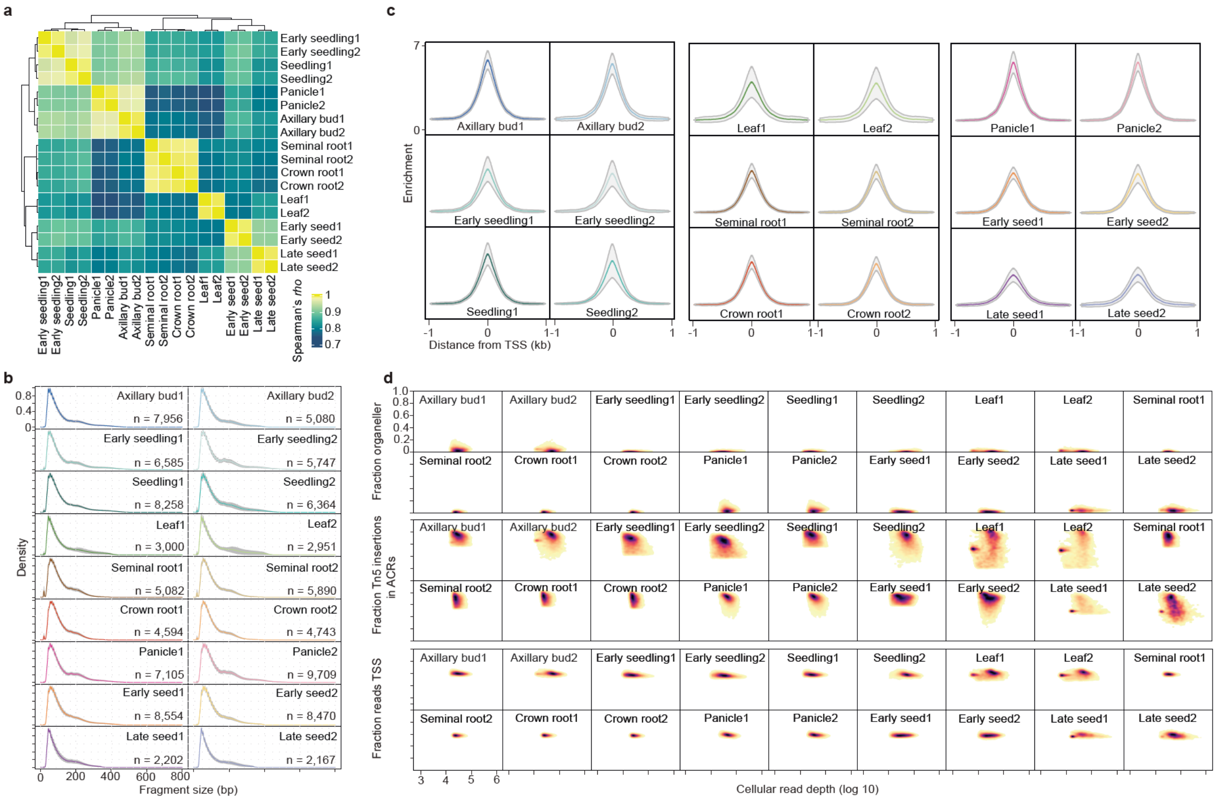

Supplementary Fig. 1. Assessment and quality control of rice scATAC-seq data. a, Comparison of scATAC-seq libraries across 18 samples from nine distinct organs based on the spearman rho matrix. **b,** Fragment length distributions for each library. The solid lines represent the average distribution of fragment lengths across cells within the sample, whereas the shaded areas indicate the standard deviation across cells within the library. The number of cells per library corresponds to panel C, D, and E. **c,** Enrichment profiles focus on 2 kb windows encompassing TSSs (transcriptional start sites) for barcodes per organ. The shaded regions illustrate the standard deviation of enrichment across cells per library. **d,** Density scatter plots depict log10-transformed barcode read depths on the x-axis against the proportions of organellar DNA, the fraction of Tn5 integration sites within ACRs denoted as FRiP, and the fraction of Tn5 integration sites aligning within 2 kb of TSSs per library.

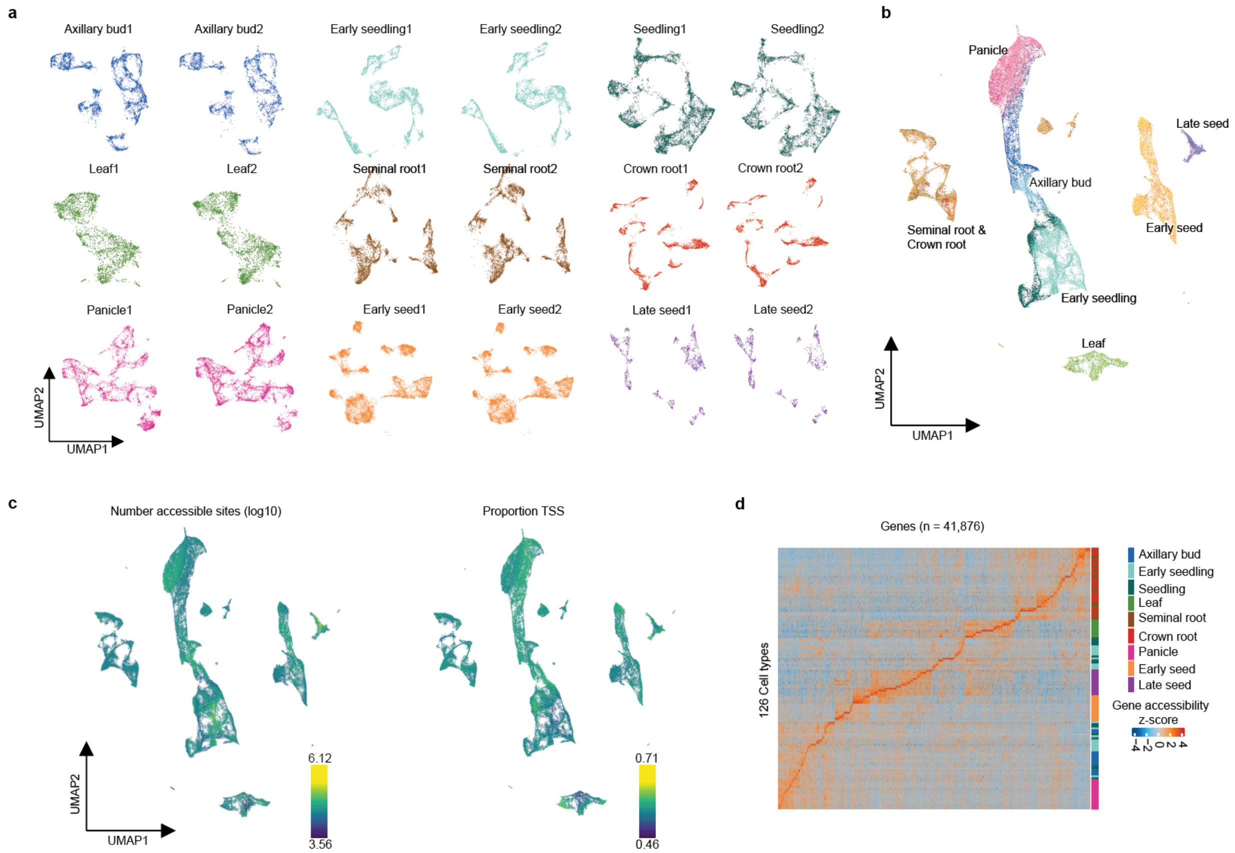

Supplementary Fig. 2. Assessment and quality control of rice scATAC-seq data for nuclei profiling in UMAP embeddings. a, UMAP embedding of nuclei colored by each library. **b,** UMAP embedding of all nuclei colored by organs. **c,** UMAP visualization of nuclei, color-coded based on the accessible sites per cell (log10) and the ratio of Tn5 integrations within 2 kb of gene TSSs per individual cell. **d,** Z-score representation of gene accessibility, spanning both the gene body and a 500 bp region upstream of TSSs, illustrated across distinct cell types.

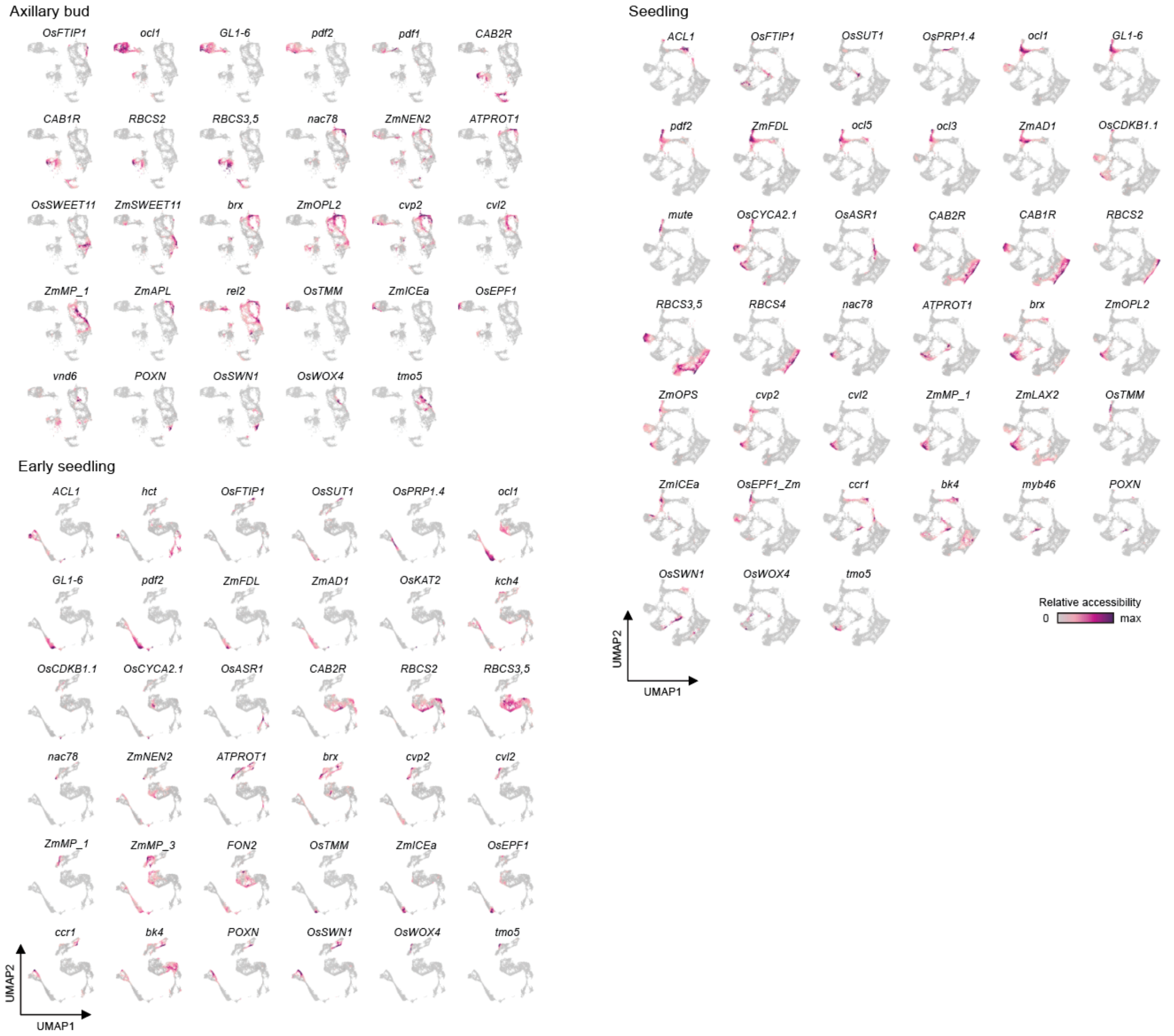

Supplementary Fig. 3. UMAP embeddings of nuclei barcodes colored by low (gray) to high (dark purple) gene chromatin accessibility scores (gene bodies plus 500 bp upstream TSSs) of cell-type-specific marker genes in axillary bud, early seedling, and seedling organs. Only a subset of the 315 markers that perform well in scATAC-seq annotation being shown in this figure. The total of 315 markers were stored in Supplementary Table 2.

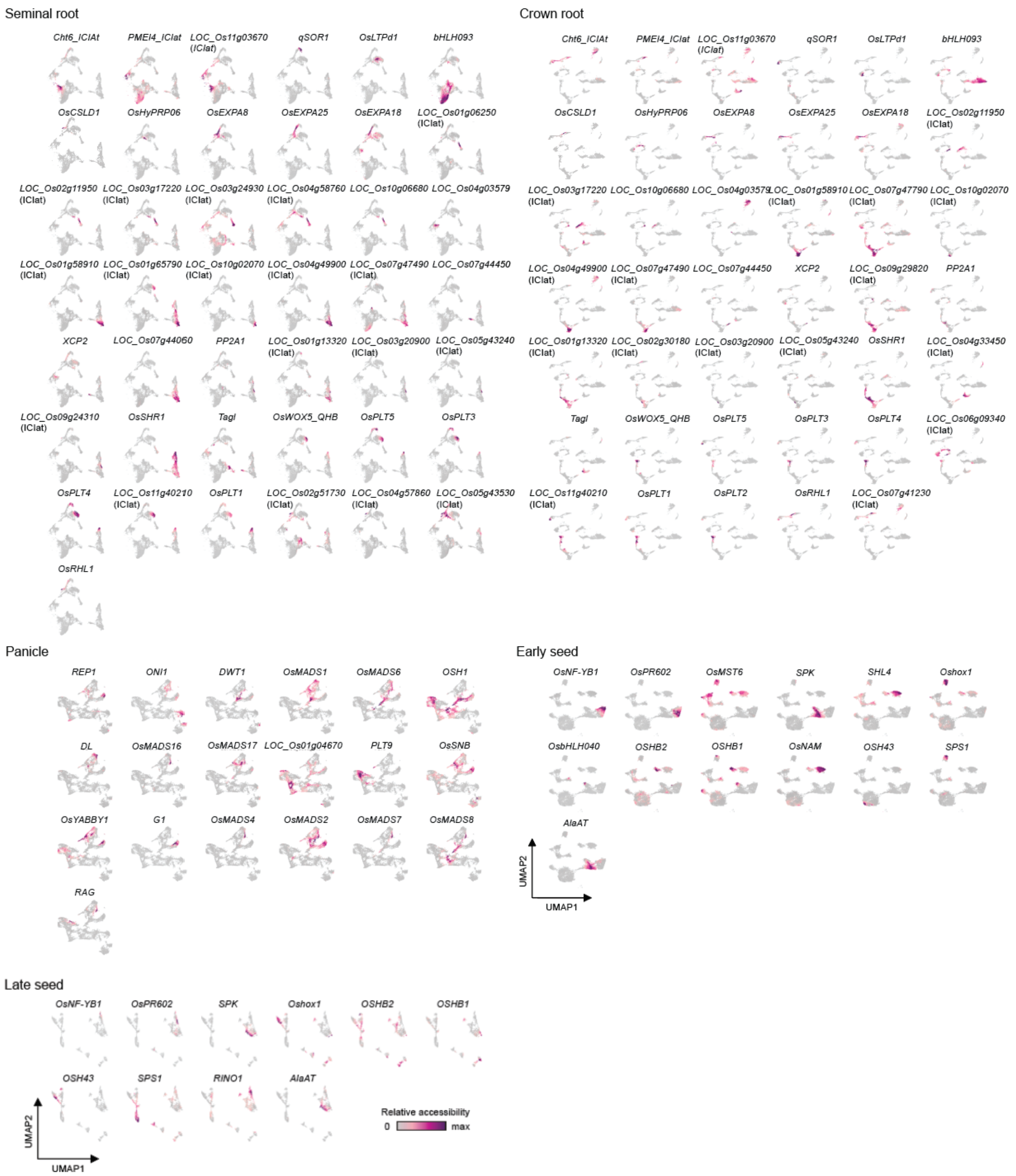

Supplementary Fig. 4. UMAP embeddings of nuclei barcodes colored by low (gray) to high (dark purple) gene chromatin accessibility scores (gene bodies plus 500 bp upstream TSSs) of cell type specific marker genes in seminal root, crown root, panicle, early seed and late seed organs. Only a subset of the 315 markers that perform well in scATAC-seq annotion being shown in this figure. The total of 315 markers were stored in Supplementary Table 2.

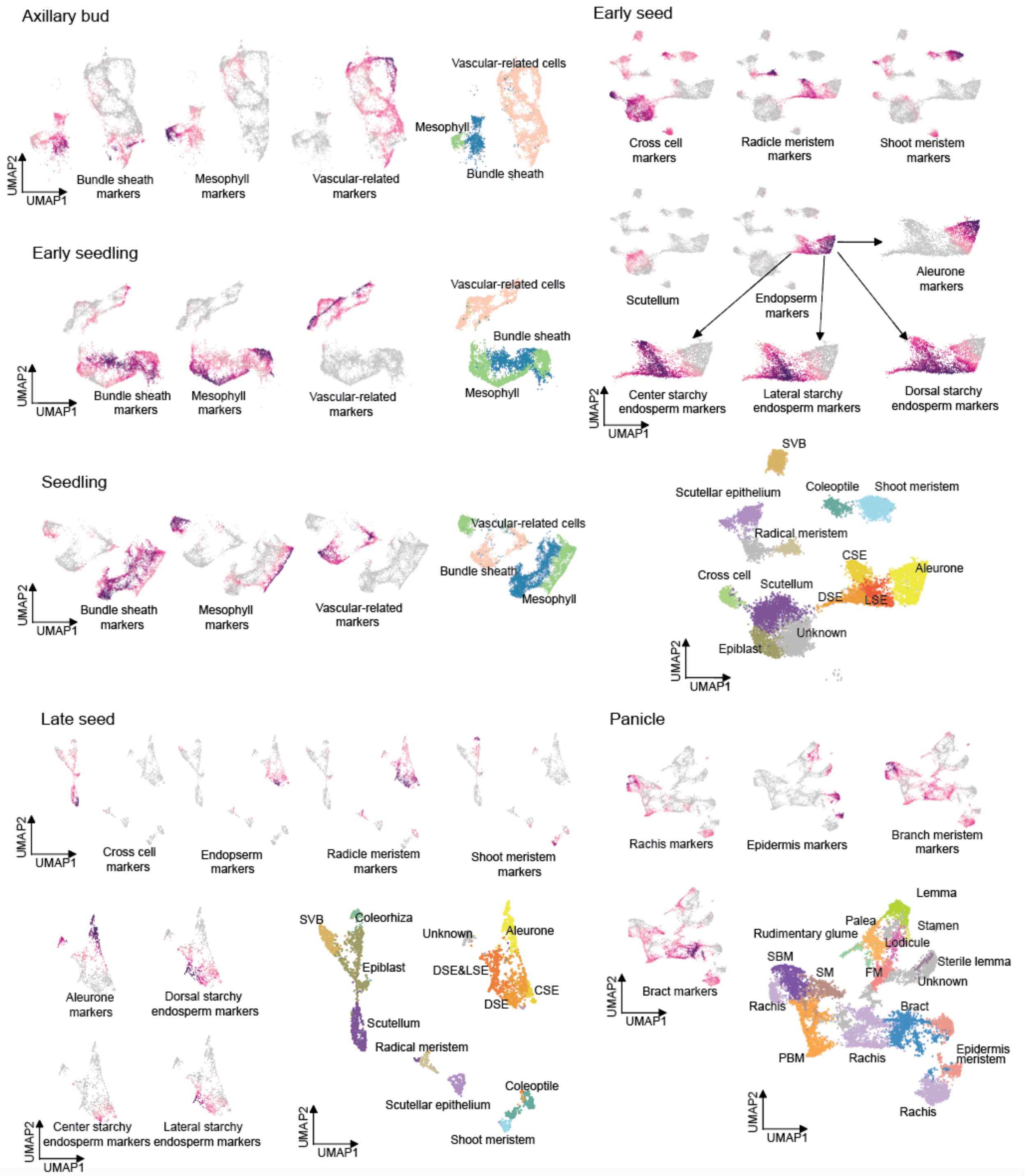

Supplementary Fig. 5. Aggregation of chromatin accessibility of markers from bulk RNA-seq and scRNA-seq data of diverse cell types.

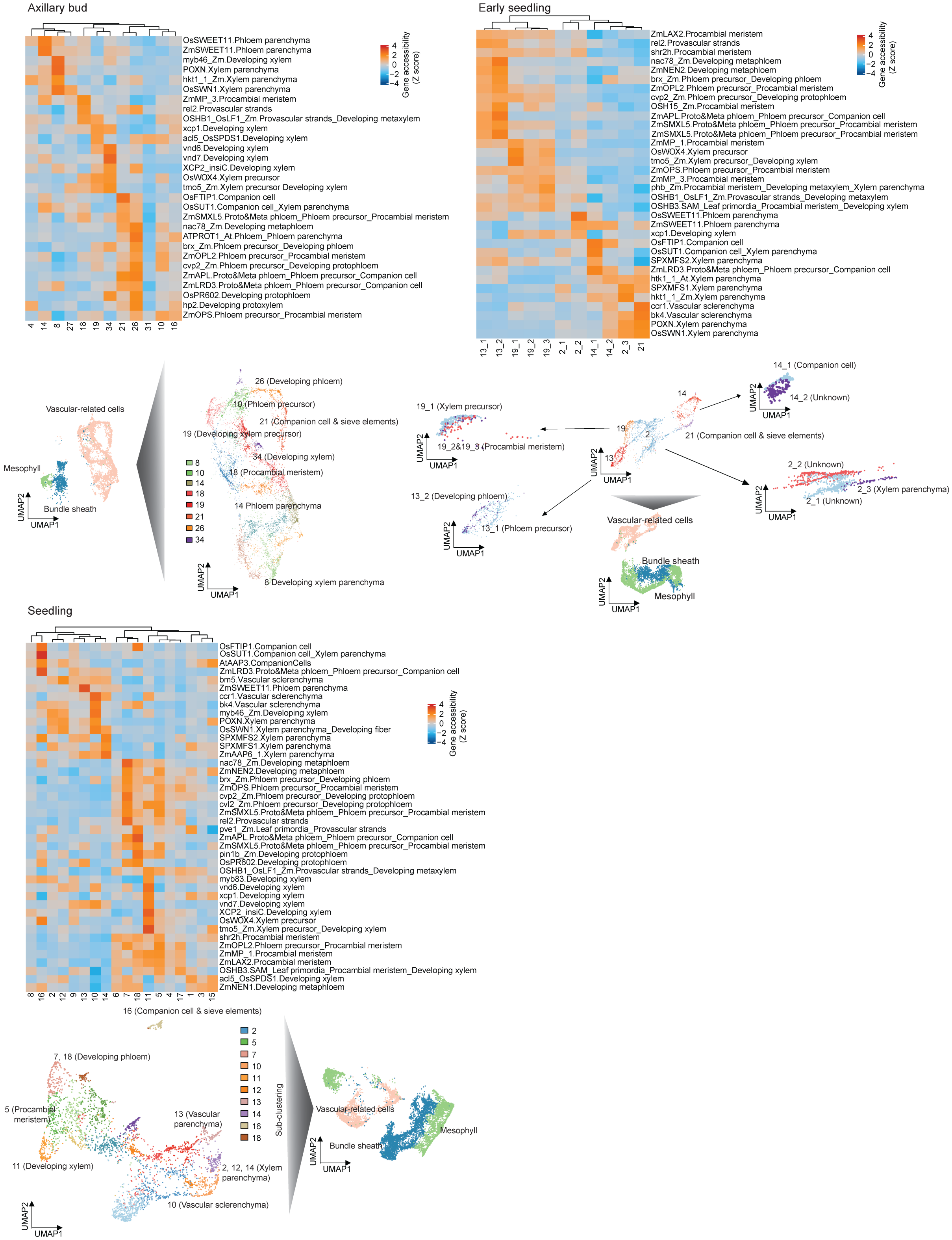

Supplementary Fig. 6. Sub-clustering and annotation of vascular-related cell identities based on the vascular-related cell-type-specific marker genes.

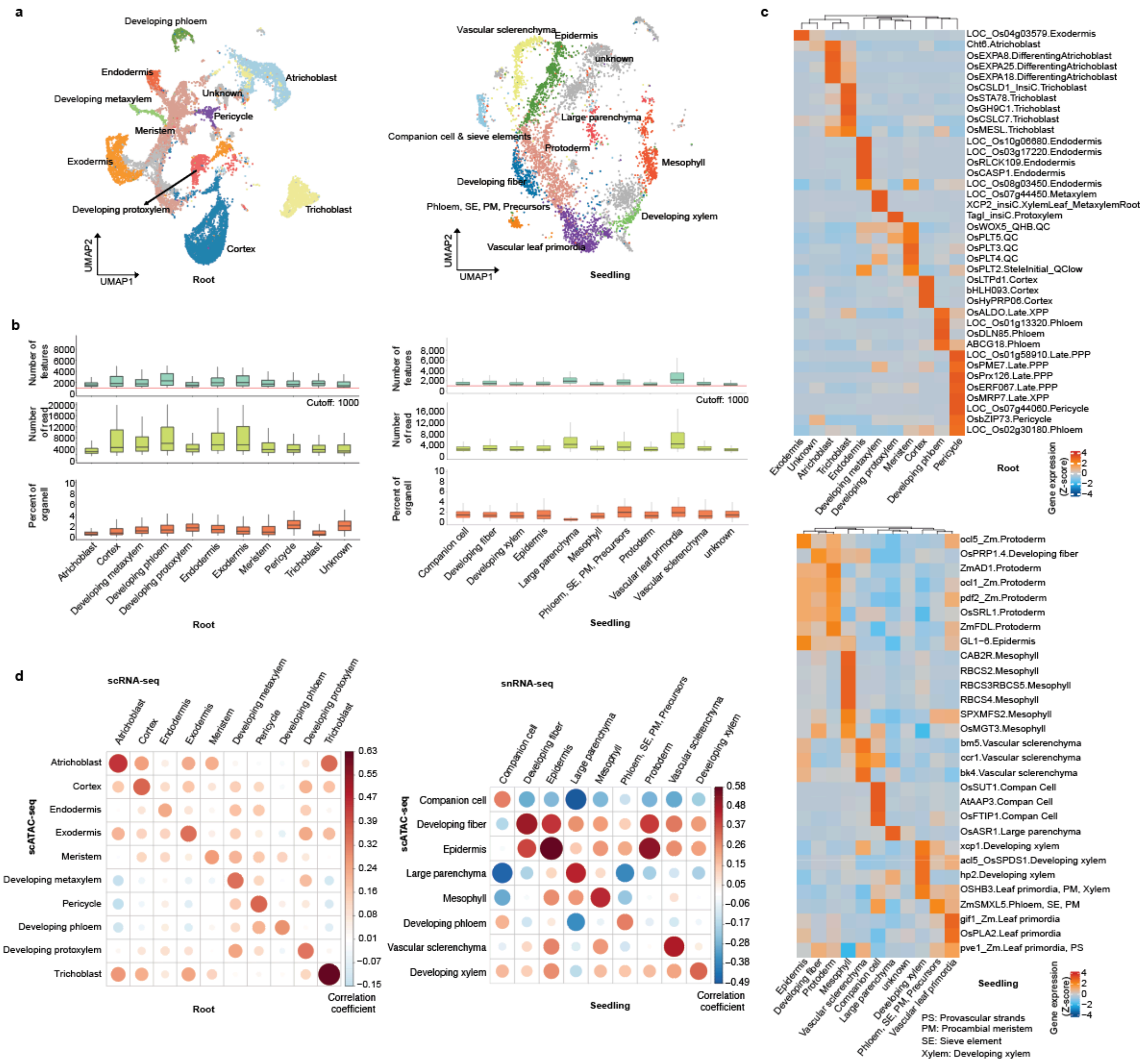

Supplementary Fig. 7. Cell identity annotation and quality controls of root scRNA-seq and seedling snRNA-seq datasets. a, Annotation of cell identities in root and seedling organs. **b**, Quality controls of datasets. The red line indicates a cutoff of filtering out cells with detected features less than 1,000. **c**, Heat map displays performance of marker genes across annotated cell clusters. **d,** Cross-cell-type correlation between gene expression (scRNA-seq/snRNA-seq) and chromatin accessibility (scATAC-seq).

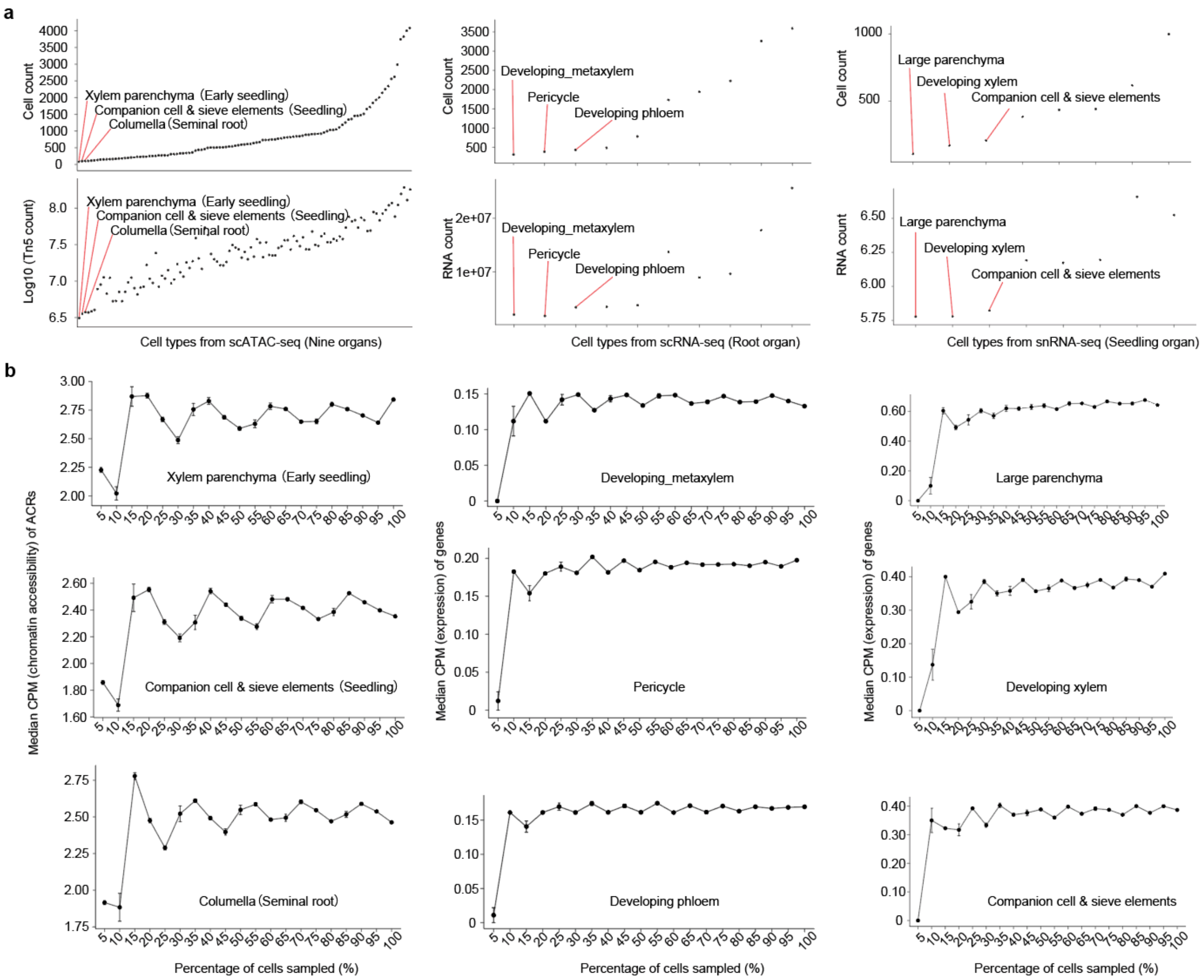

Supplementary Fig. 8. Stability analysis of CPM values for ACRs and genes in cell types with the lowest cell counts. **a,** Cell types were ranked by cell count from scATAC-seq (across nine organs) and scRNA-seq/snRNA-seq (from root and seedling organs). The lower panels show the $\log_{10}$ (Tn5 counts) for each cell type, corresponding to the ranking above. For the scRNA-seq/snRNA-seq data, RNA counts are shown instead of Tn5 counts. **b,** Median CPM values of ACRs (for scATAC-seq) or genes (for scRNA-seq/snRNA-seq) during downsampling analysis. Sampling percentages (e.g., 5 represents 5% of cells) were applied to the three lowest-abundance cell types identified in **panel a**.

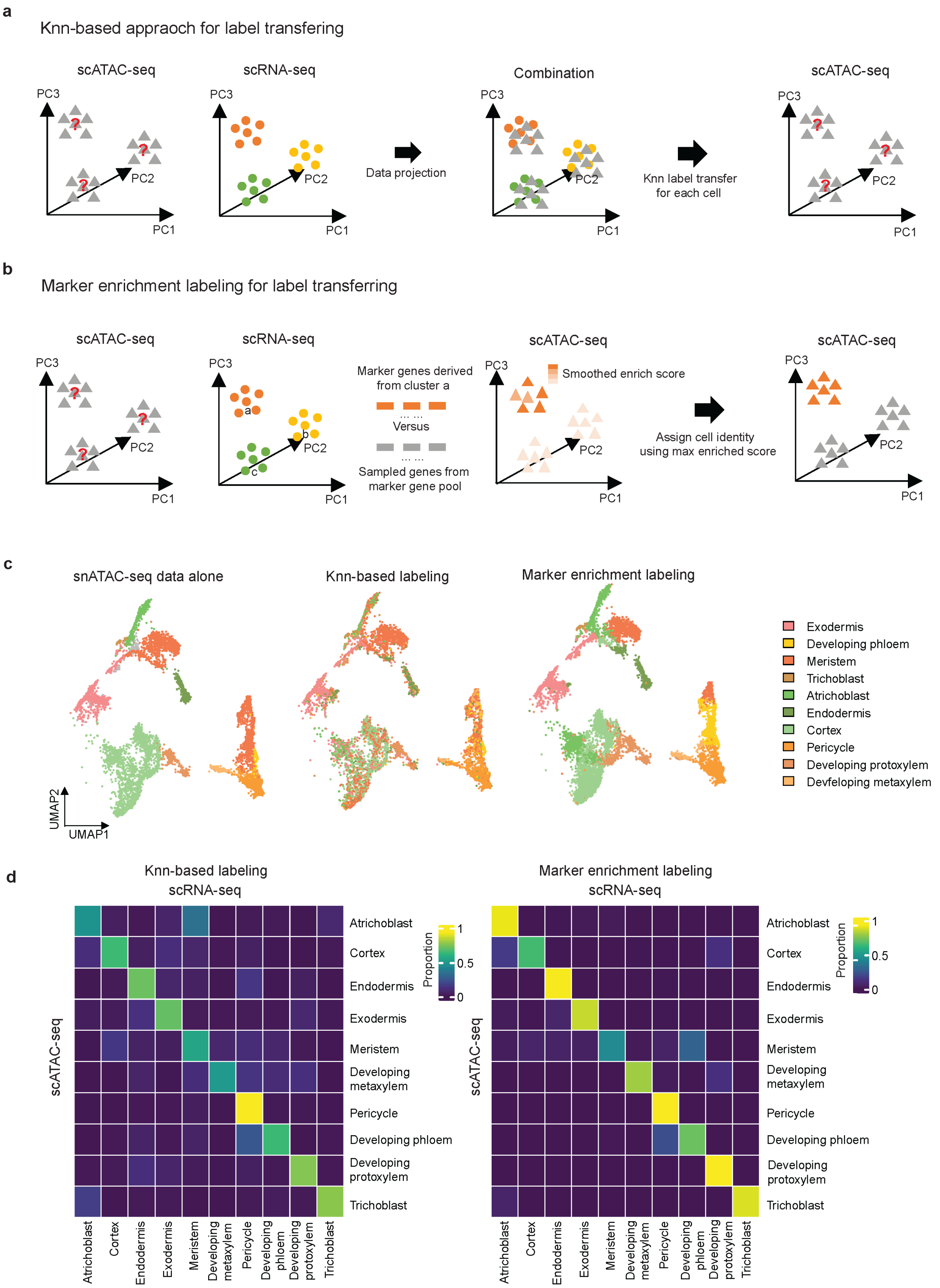

Supplementary Fig. 9. Two approaches used to compare scRNA-seq and scATAC-seq atlases in the root organ. a-b, Schematic of KNN-based (**a**) and marker enrichment (**b**) approaches for transferring labels from scRNA-seq data to scATAC-seq data. **c**, UMAP embedding colored by labels made by scATAC-seq data alone and labels derived from the KNN-based and marker enrichment approaches. **d**, Proportions of annotated cells by scRNA-seq data to cells annotated by scATAC-seq.

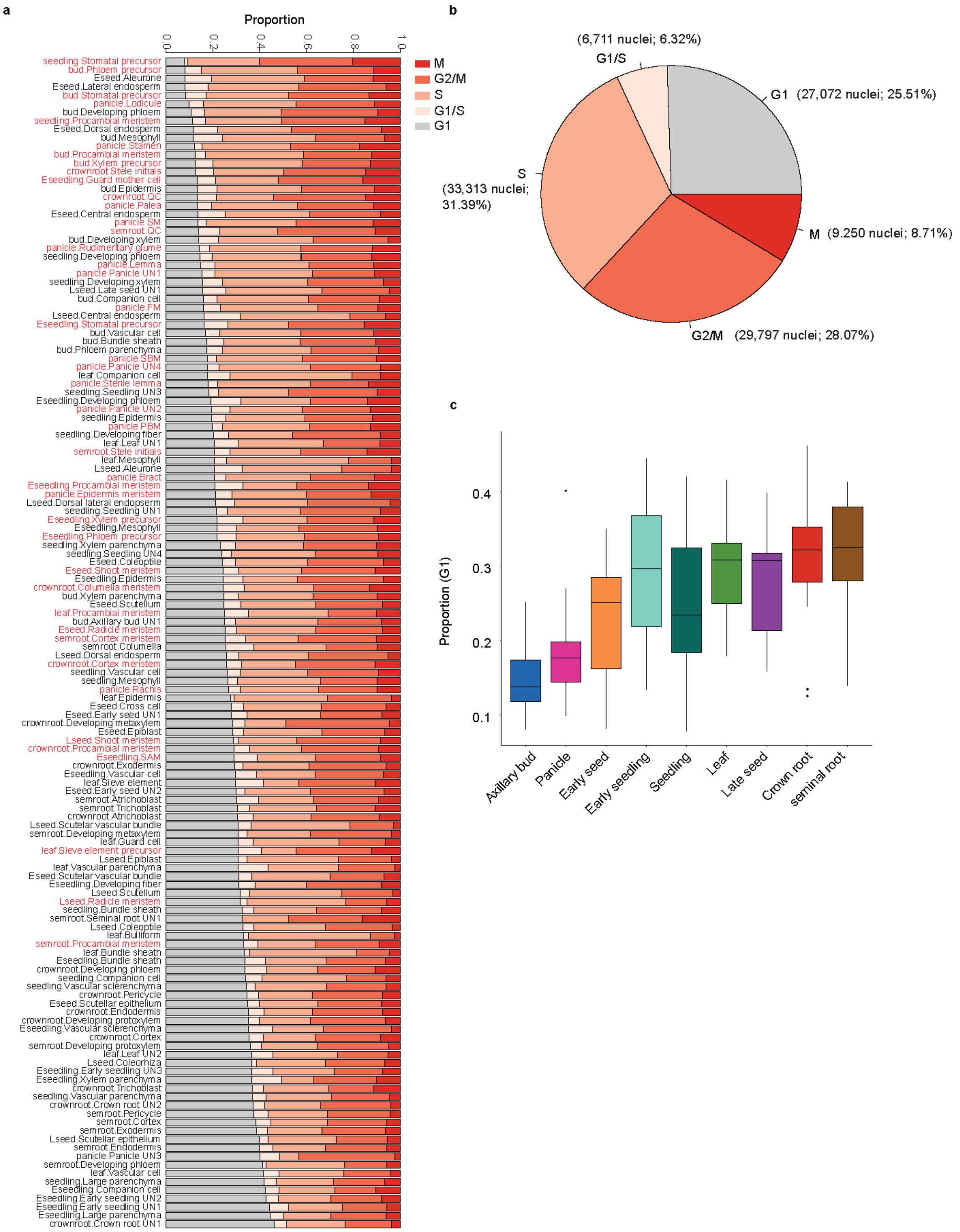

Supplementary Fig. 10. Prediction of cell cycle stage of cells in 138 cell states. a, Proportion of cells in cell cycle stages per cell state. G1: Gap1. S1: synthesis. G2: Gap2. M: Mitosis. G1/S: G1-to-S phase transition. G2/M: G2-to-S phase transition. **b,** Proportion of cells within five cell cycle stages. **c,** Proportion of cells predicted in G1 phase in each organ.

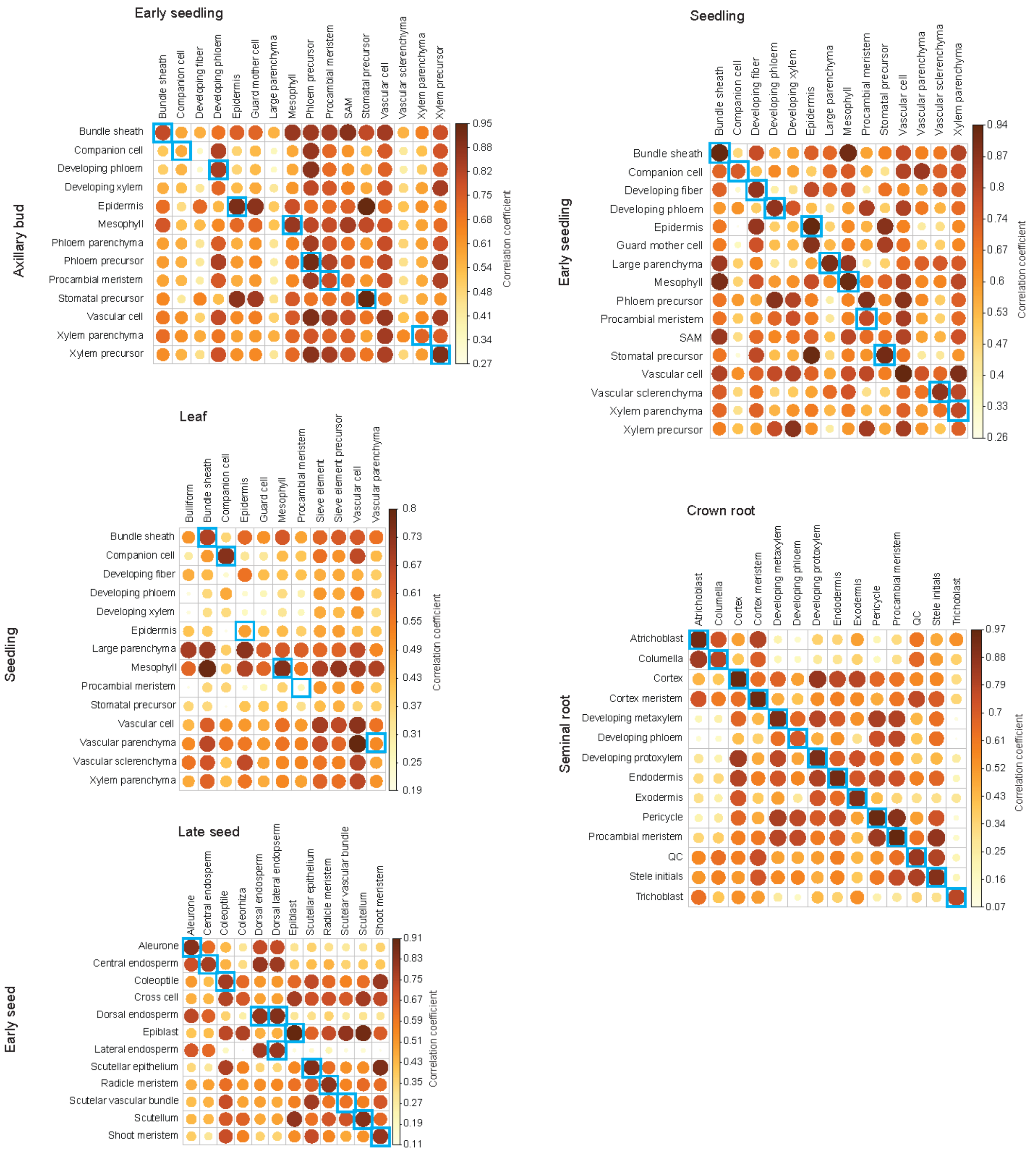

Supplementary Fig. 11. Spearman correlations of gene chromatin accessibilities between different pairs of organs. The correlations are computed for a set of the top 500 most variable genes using chromatin accessibilities across all cell types. The blue frames are used to highlight corresponding cell types that are annotated between two organs.

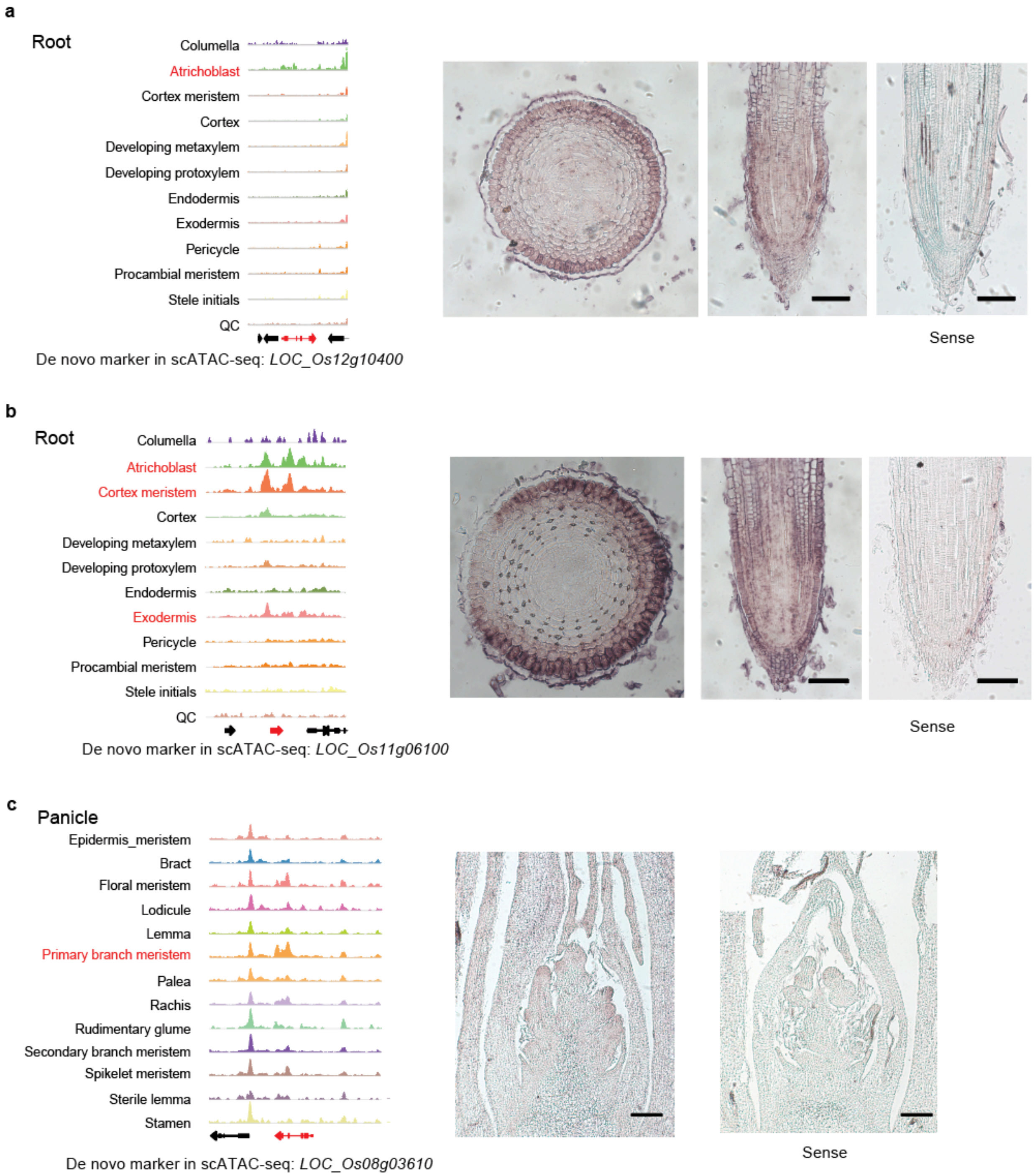

Supplementary Fig. 12. Validating de novo markers of root and panicle tissues identified from scATAC-seq in rice through RNA *in situ* hybridization. The left panels illustrate aggregate chromatin accessibility patterns around de novo marker genes predicted from scATAC-seq data. The cell types highlighted in red indicate comparatively higher chromatin accessibility surrounding the target markers in comparison to other cell types. The gene models highlighted in red correspond to the target marker genes. The right panel, positioned below the aggregate chromatin accessibility panels, displays results obtained from RNA *in situ* hybridization. The bars indicate 200 μm in *LOC_Os12g10400* (**a**) and *LOC_Os11g06100* (**b**), 100 μm in *LOC_Os08g03610* (**c**).

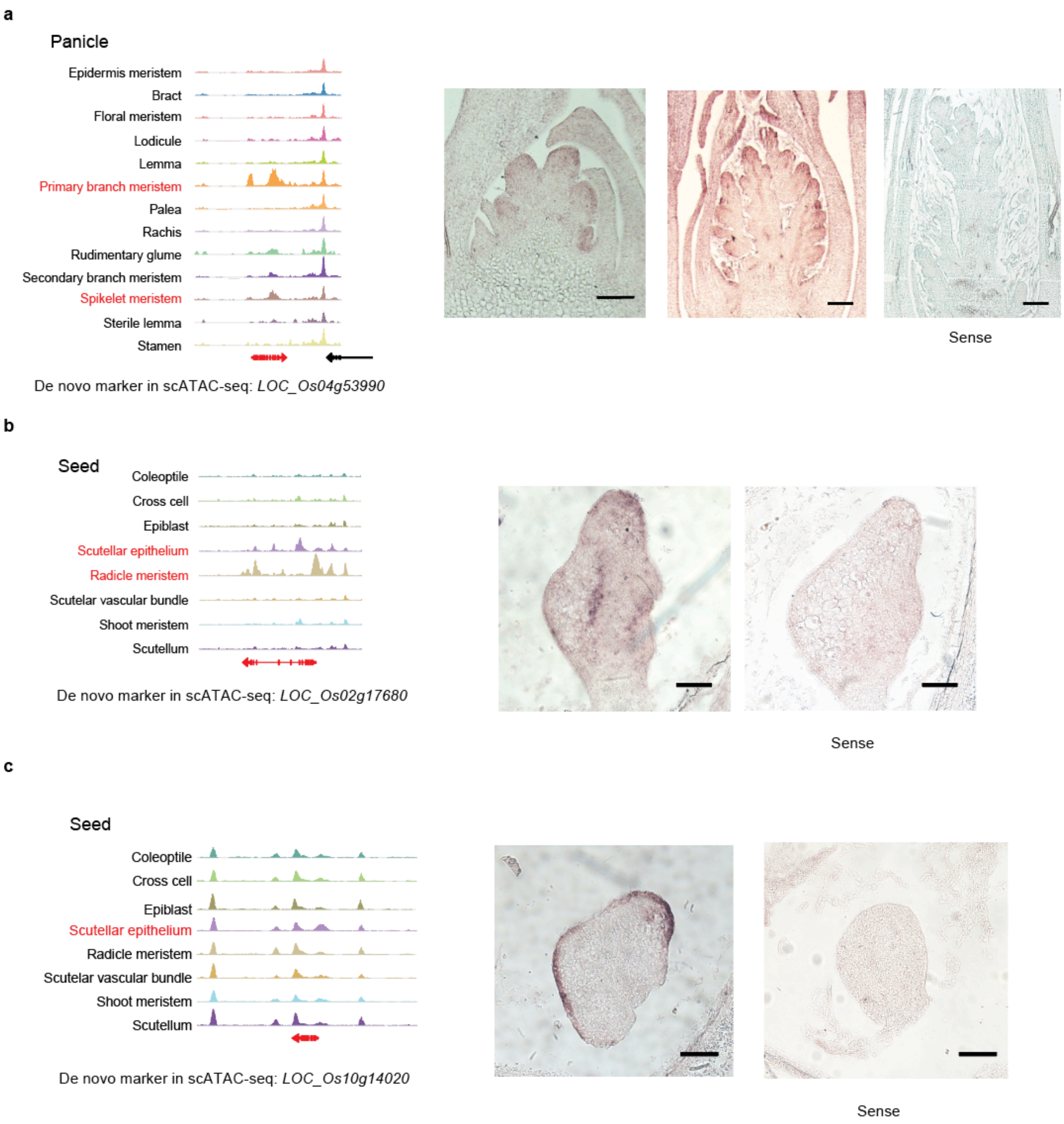

Supplementary Fig. 13. Validating de novo markers of panicle and seed tissues identified from scATAC-seq in rice through RNA *in situ* hybridization. The left panels illustrate aggregate chromatin accessibility patterns around de novo marker genes predicted from scATAC-seq data. The cell types highlighted in red indicate comparatively higher chromatin accessibility surrounding the target markers in comparison to other cell types. The gene models highlighted in red correspond to the target marker genes. The right panel, positioned below the aggregate chromatin accessibility panels, displays results obtained from RNA *in situ* hybridization. The bars indicate 50 µm, 20µm, 100µm in *LOC_Os04g53990* (**a**), 50 µm in *LOC_Os02g17680* (**b)** and *LOC_Os10g14020* (**c**).

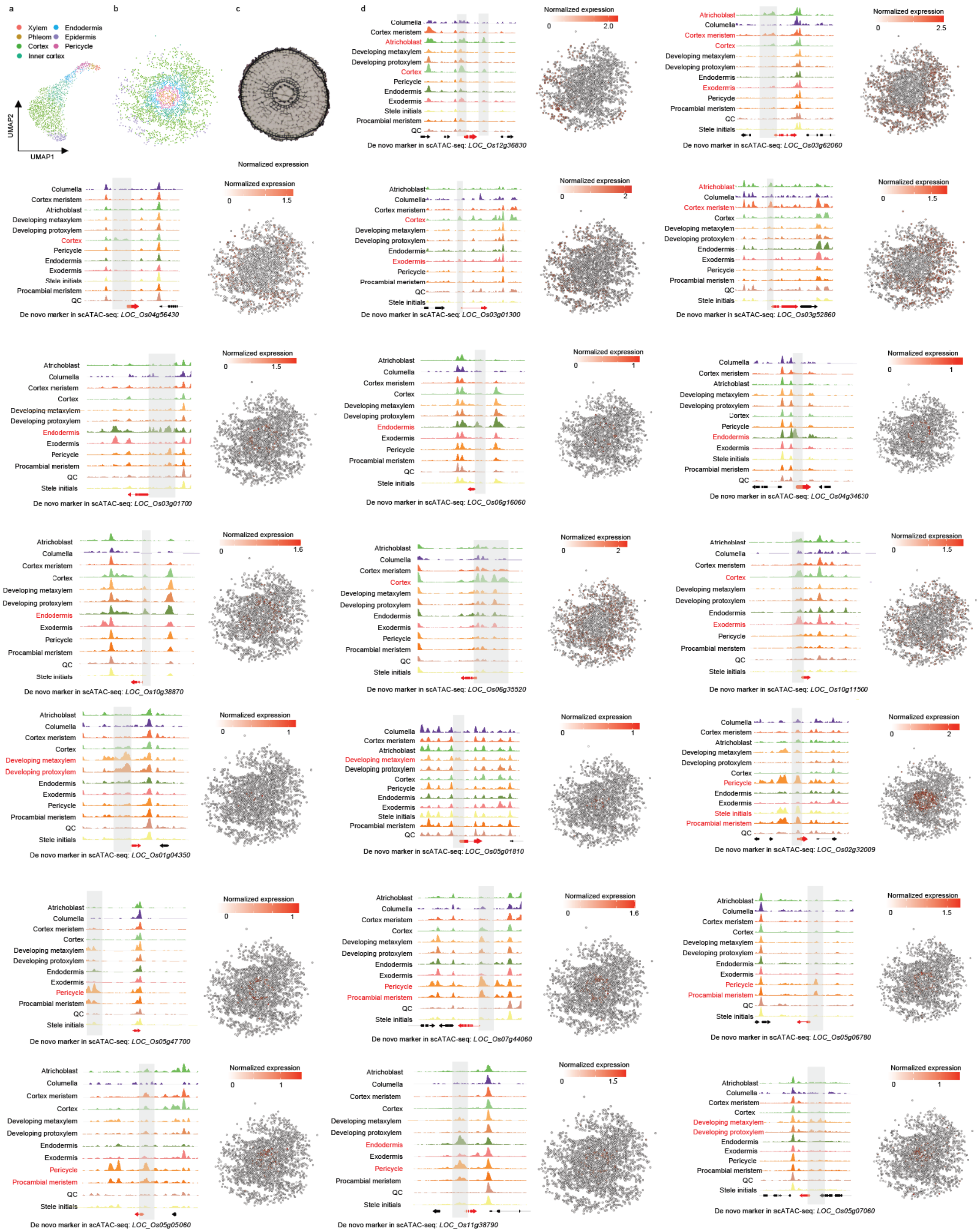

Supplementary Fig. 14. Validating *de novo* markers identified from scATAC-seq in rice through slide-seq. a, UMAP embedding of the root spatial spots colored by cell types. **b**, Spatial mapping of root cross-section cell types. **c**, The image of the root cross-section. **d**, The left panels illustrate aggregate chromatin accessibility patterns around 20 de novo marker genes predicted from scATAC-seq data. The cell types highlighted in red indicate comparatively higher chromatin accessibility surrounding the target markers in comparison to other cell types. The right panels show the normalized expression of the de novo markers in the spatial transcriptome.

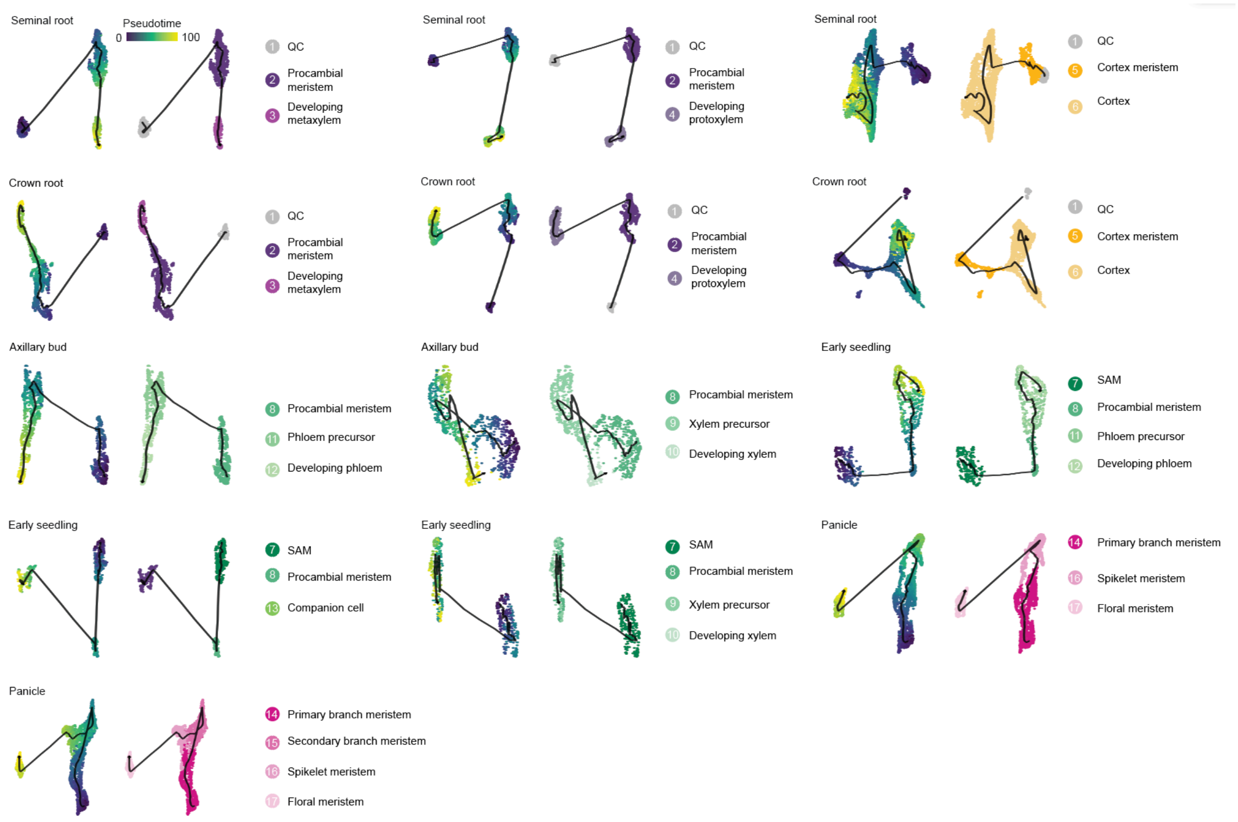

Supplementary Fig. 15. Cell-type development in 13 trajectories.

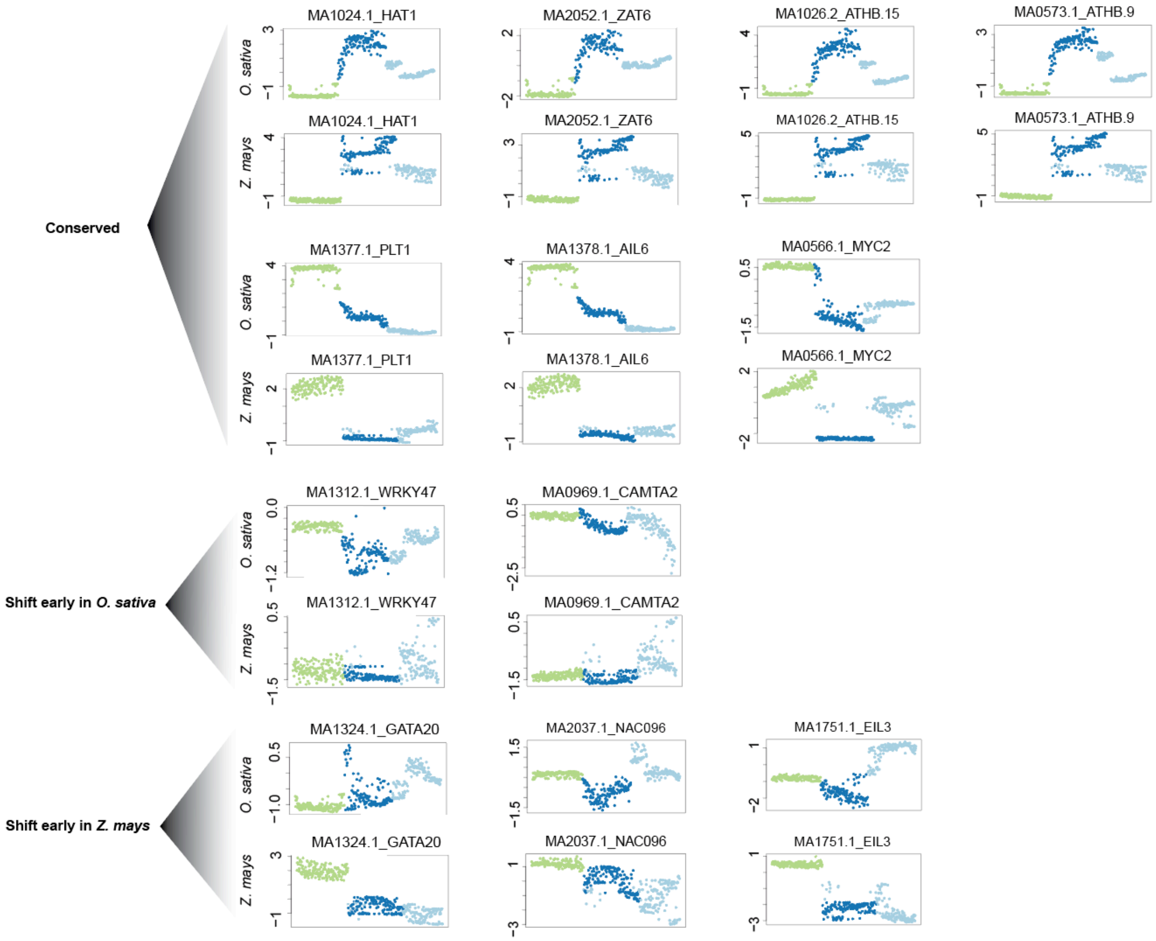

Supplementary Fig. 16. Comparison of relative motif accessibility of TF motifs shown in Extended Data Fig. 2g along the pseudotime between O. sativa and Z. mays.

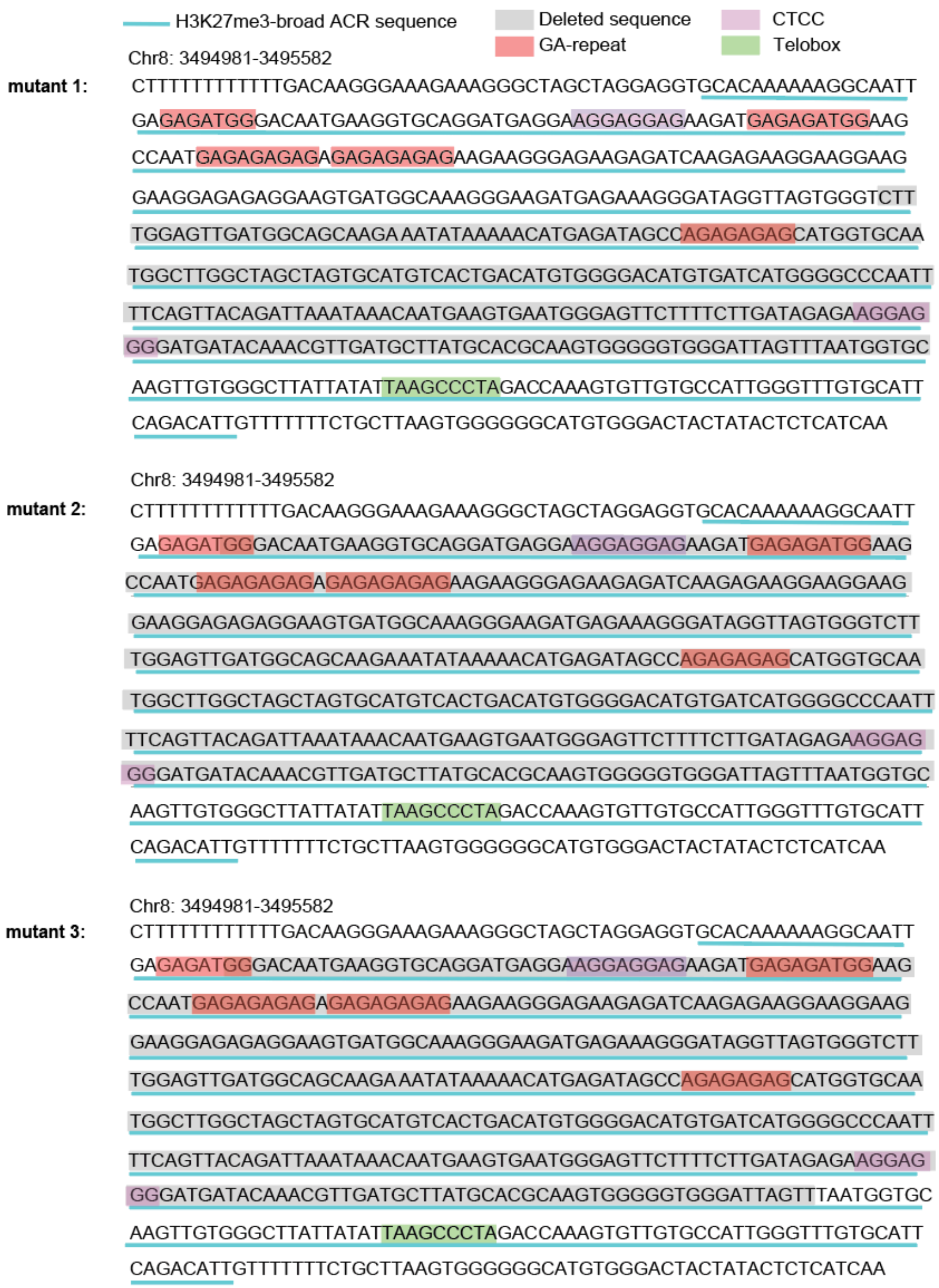

Supplementary Fig. 17. Sequences of three mutants, each labeled with deleted regions and PRE motifs within an H3K27me3-broad ACR.

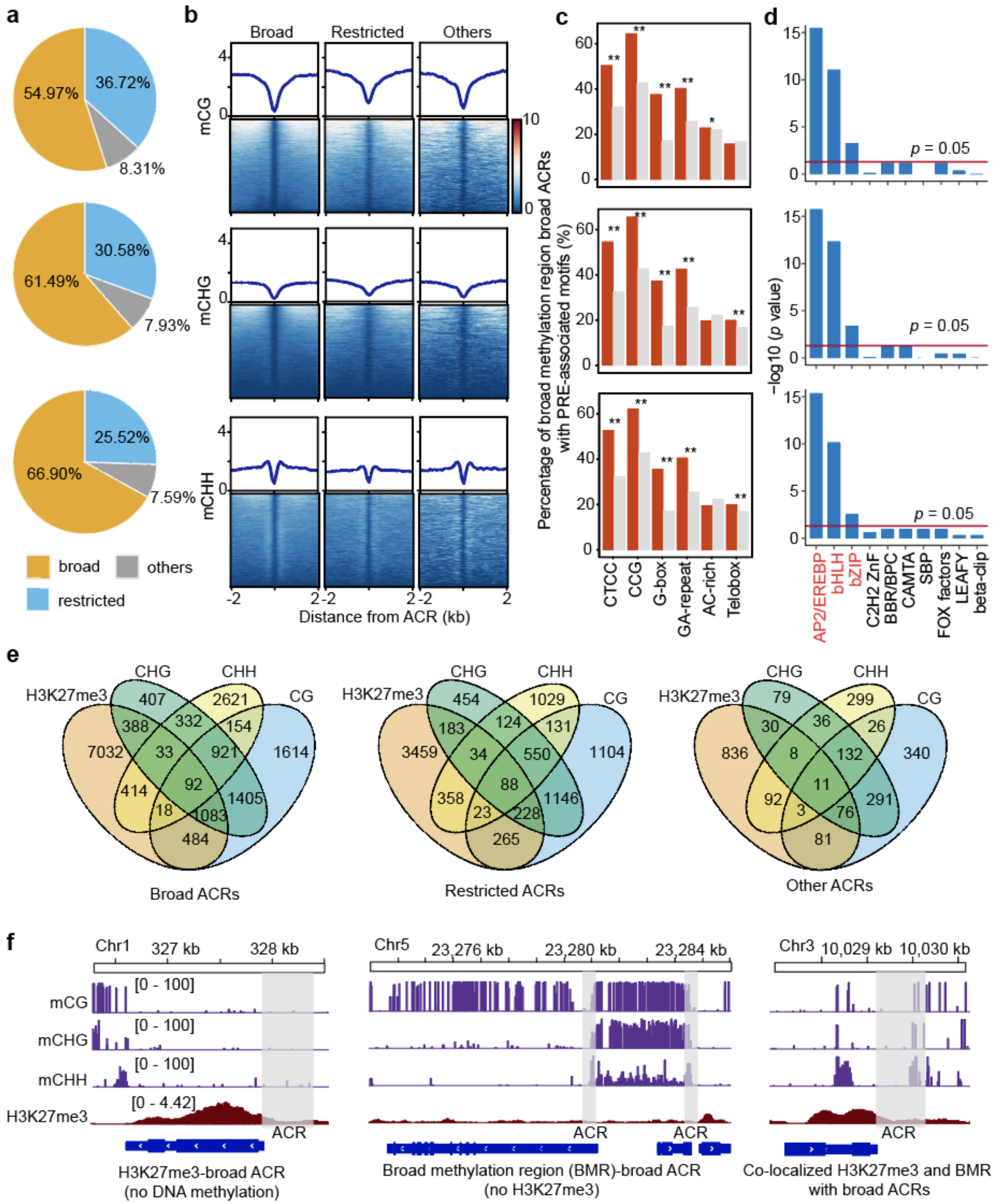

Supplementary Fig. 18. Context-dependent crosstalk between DNA methylation and ACRs in rice. **a,** Proportional overlap of mCG/mCHG/mCHH BMRs with different categories of ACRs. **b,** Leaf CG/CHG/CHH methylation reads near summits of distinct ACR groups. **c,** Percentage of mCG/mCHG/mCHH BMRs-broad ACRs in *O. sativa* capturing six known motifs enriched in PREs in *A. thaliana*. ** indicate *p* value < 0.01, which was performed by the Binomial test (alternative = 'greater'). **d,** Three TF families, highlighted in red, were significantly enriched in mCG/mCHG/mCHH BMRs-broad

ACRs. The motif data were collected from 568 TFs from *A. thaliana* belonging to 24 families within the JASPAR database (Castro-Mondragon et al. 2022). The p value was computed using a hypergeometric test (alternative = ‘greater’). **e,** Multi-set Venn diagram quantifying co-occupancy of H3K27me3-, CG BMRs-, CHG BMRs-, and CHH BMRs-associated ACRs. **f,** Genome browser tracks exemplifying three regulatory states of H3K27me3 and DNA methylation: H3K27me3-broad ACRs (no DNA methylation); BMRs-broad ACRs (no H3K27me3); Co-localized H3K27me3 and BMRs with broad ACRs.

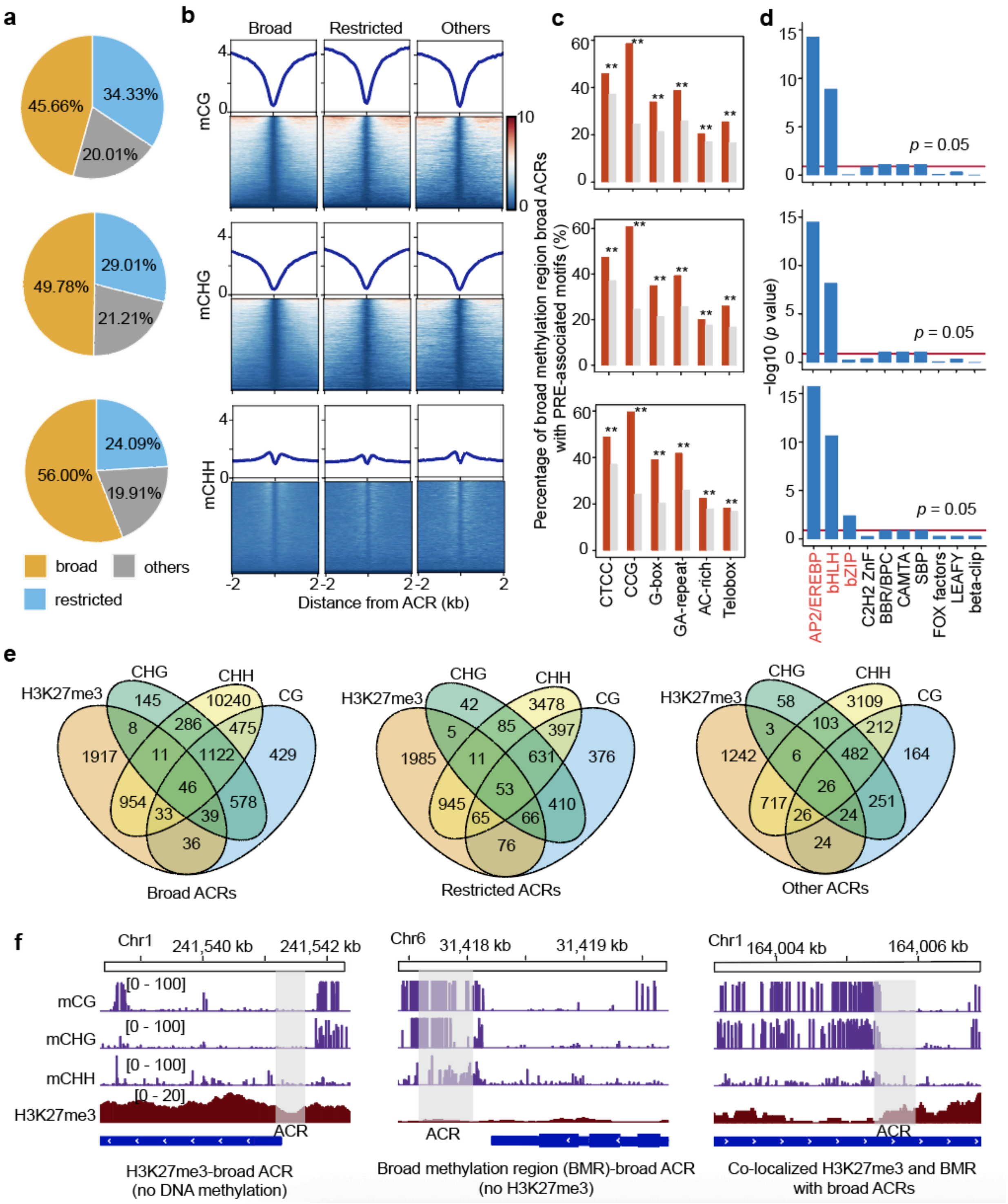

Supplementary Fig. 19. Context-dependent crosstalk between DNA methylation and ACRs in maize. **a,** Proportional overlap of mCG/mCHG/mCHH BMRs with different categories of ACRs. **b,** Leaf CG/CHG/CHH methylation reads near summits of distinct ACR groups. **c,** Percentage of mCG/mCHG/mCHH BMRs-broad ACRs in *Z. mays* capturing six known motifs enriched in PREs in *A. thaliana*. ** indicate *p* value < 0.01, which was performed by the Binomial test (alternative = ‘greater’). **d,** Three TF families, highlighted in red, were significantly enriched in mCG/mCHG/mCHH BMRs-broad

ACRs. The motif data were collected from 568 TFs from *A. thaliana* belonging to 24 families within the JASPAR database (Castro-Mondragon et al. 2022). The *p* value was computed using a hypergeometric test (alternative = 'greater'). **e,** Multi-set Venn diagram quantifying co-occupancy of H3K27me3-, CG BMRs-, CHG BMRs-, and CHH BMRs-associated ACRs. **f,** Genome browser tracks exemplifying three regulatory states of H3K27me3 and DNA methylation: H3K27me3-broad ACRs (no DNA methylation); BMRs-broad ACRs (no H3K27me3); Co-localized H3K27me3 and BMRs with broad ACRs.

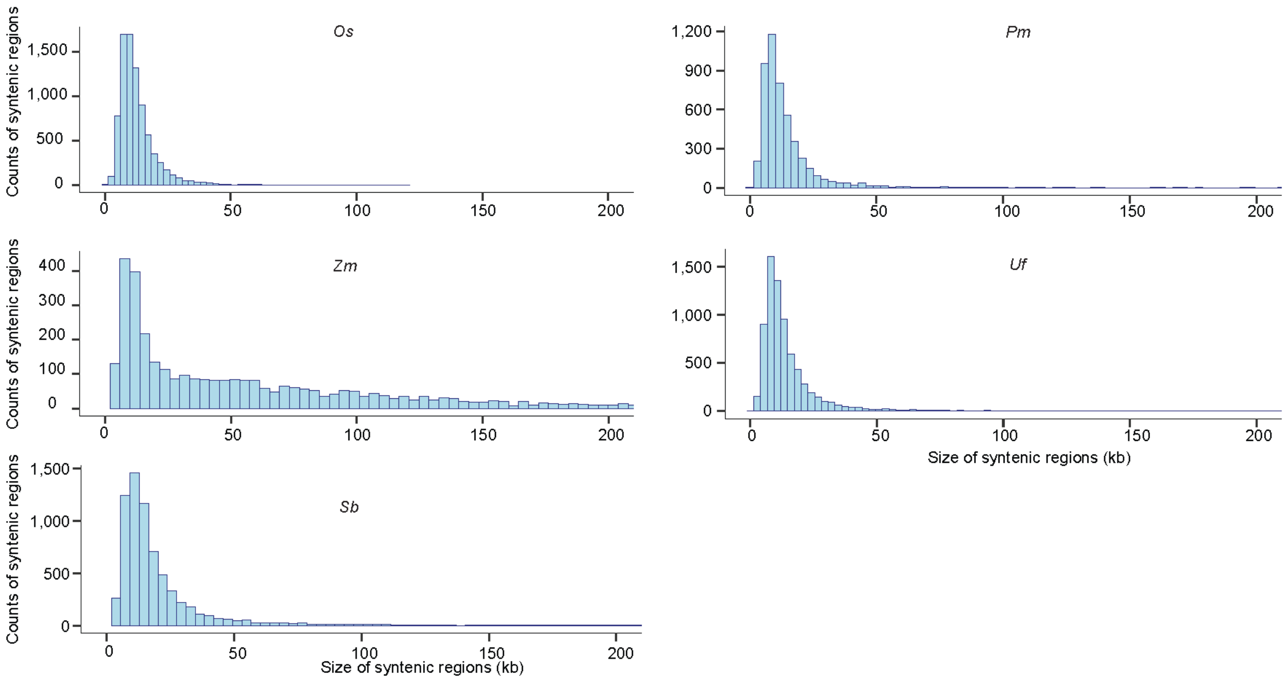

Supplementary Fig. 20. Distribution of syntenic region size of all five species of grasses.

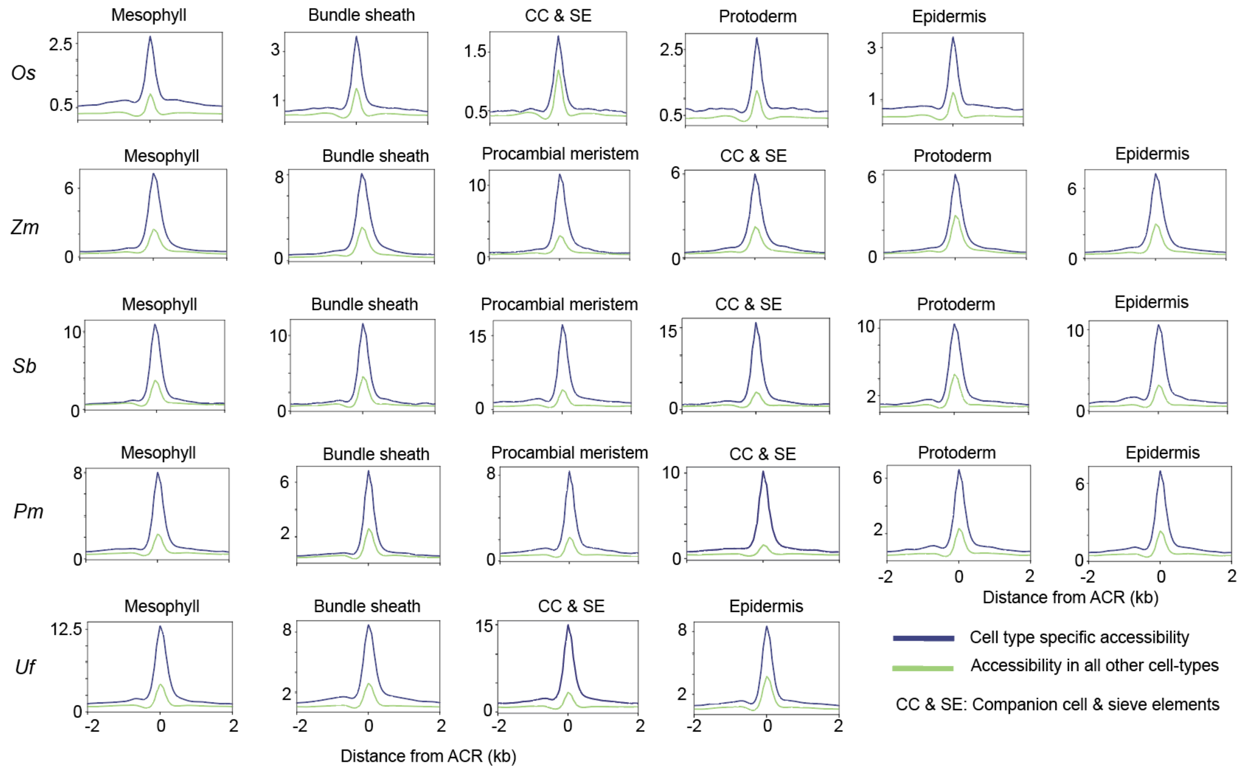

Supplementary Fig. 21. Metaplot of ATAC signal in cell-type-specific ACRs identified as being specific to a particular cell type as compared to the ATAC-seq signal of all the other cells for that same ACR.

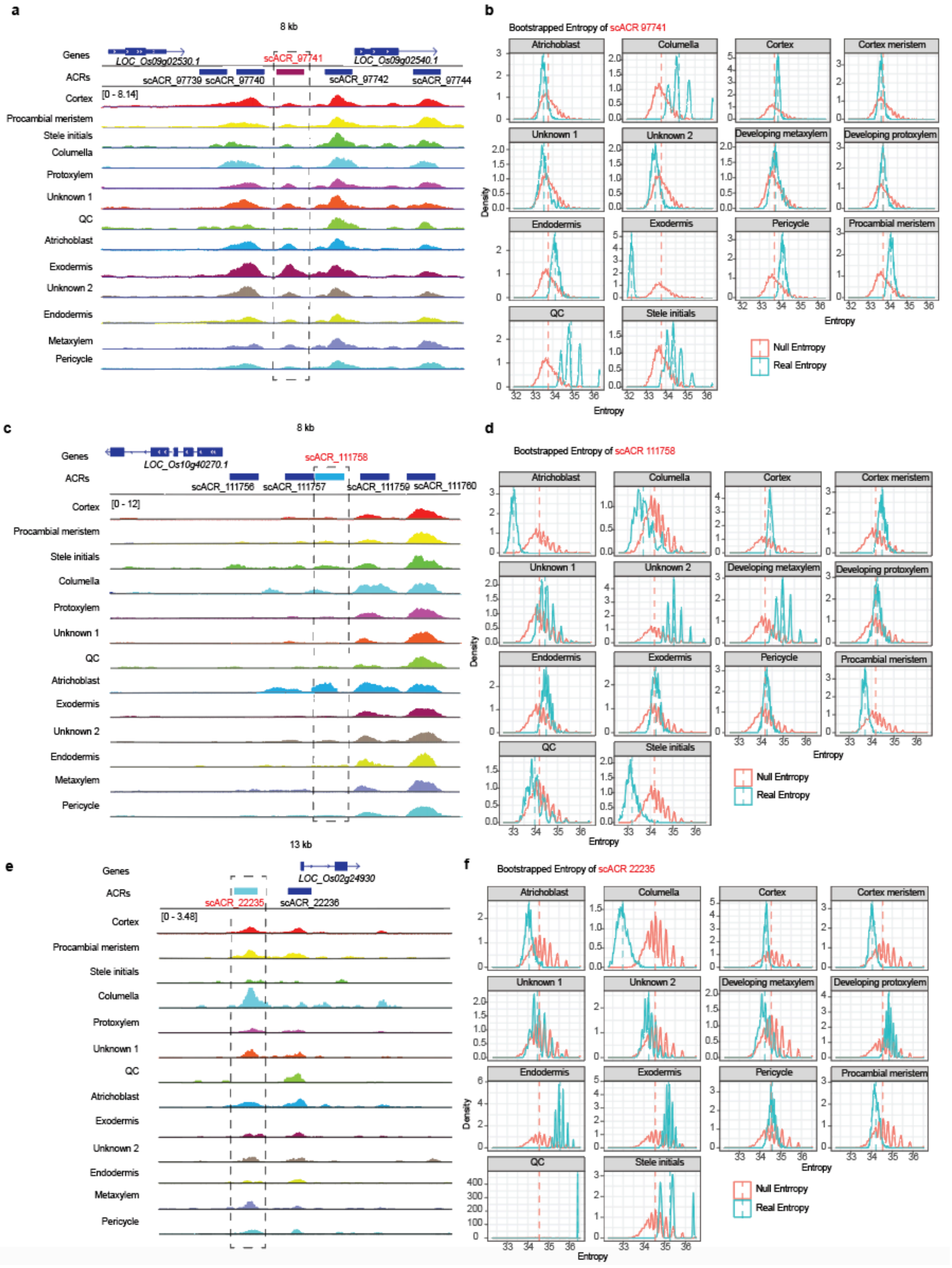
a
8 kb
Genes
LOC_Os09g02530.1
scACR_97741
LOC_Os09g02540.1
ACRs
scACR_97739 scACR_97740
scACR_97742
scACR_97744
Cortex
[0 - 8.14]
Procambial meristem
Stele initials
Columella
Protoxylem
Unknown 1
QC
Atrichoblast
Exodermis
Unknown 2
Endodermis
Metaxylem
Pericycle
b
Bootstrapped Entropy of scACR 97741
Atrichoblast
Columella
Cortex
Cortex meristem
Unknown 1
Unknown 2
Developing metaxylem
Developing protoxylem
Endodermis
Exodermis
Pericycle
Procambial meristem
QC
Stele initials
Density
Entropy
Null Entropy
Real Entropy
c
8 kb
Genes
LOC_Os10g40270.1
scACR_111758
ACRs
scACR_111756 scACR_111757
scACR_111759 scACR_111760
Cortex
[0 - 12]
Procambial meristem
Stele initials
Columella
Protoxylem
Unknown 1
QC
Atrichoblast
Exodermis
Unknown 2
Endodermis
Metaxylem
Pericycle
d
Bootstrapped Entropy of scACR 111758
Atrichoblast
Columella
Cortex
Cortex meristem
Unknown 1
Unknown 2
Developing metaxylem
Developing protoxylem
Endodermis
Exodermis
Pericycle
Procambial meristem
QC
Stele initials
Density
Entropy
Null Entropy
Real Entropy
e
13 kb
Genes
LOC_Os02g24930
ACRs
scACR_22235
scACR_22236
Cortex
[0 - 3.48]
Procambial meristem
Stele initials
Columella
Protoxylem
Unknown 1
QC
Atrichoblast
Exodermis
Unknown 2
Endodermis
Metaxylem
Pericycle
f
Bootstrapped Entropy of scACR 22235
Atrichoblast
Columella
Cortex
Cortex meristem
Unknown 1
Unknown 2
Developing metaxylem
Developing protoxylem
Endodermis
Exodermis
Pericycle
Procambial meristem
QC
Stele initials
Density
Entropy
Null Entropy
Real Entropy

Supplementary Fig. 22. Examples of genome browser shots of assigned cell-type-specific ACRs and their corresponding entropy calculations. **a/c/e,** Browser shots of three cell-type-specific ACRs identified in this study, all from the crown root dataset. The tracks display accessibility normalized equally across all cell types and scaled for each example to facilitate direct comparison. The cell-type-specific ACR is color-coded to match the assigned cell type and is highlighted with a dashed box. **b/d/f,** Graphs showing entropy calculations for the cell-type-specific ACRs using our bootstrapped approach. Each graph represents the entropy calculation for the respective ACR across all cell types. The Y-axis shows density, and the X-axis shows the calculated entropy. Red lines represent the null population where cells were randomly sampled and cell types scrambled. Blue lines represent the bootstrapped real populations of cells. Dashed lines indicate the medians. In this example exodermis has a significant p value of .001.

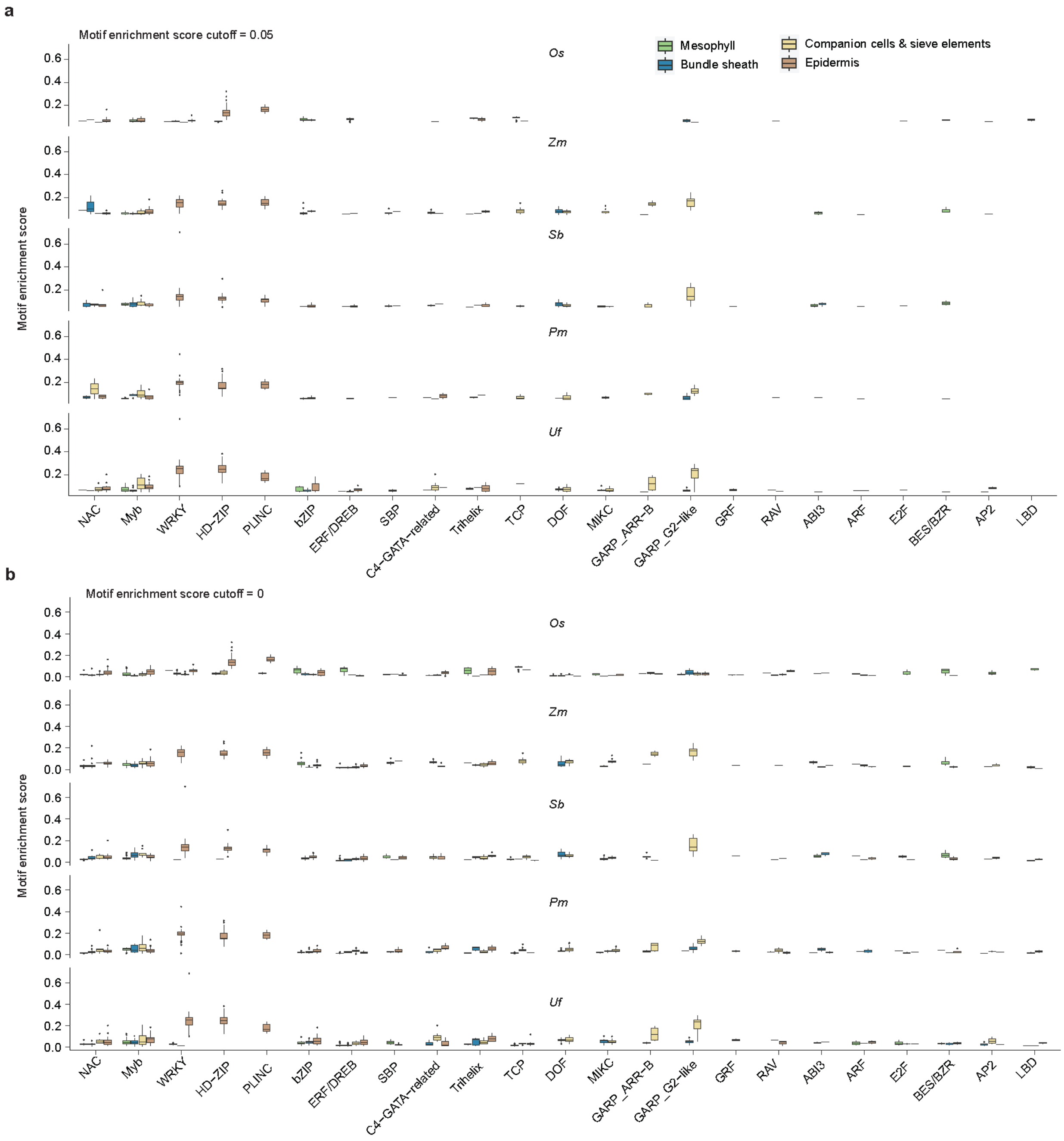

Supplementary Fig. 23. Boxplots illustrate TF motif enrichment scores for all motif family members across various species for each cell type. The motif enrichment score cutoff was set to 0 (**a**) and 0.05 (**b**).

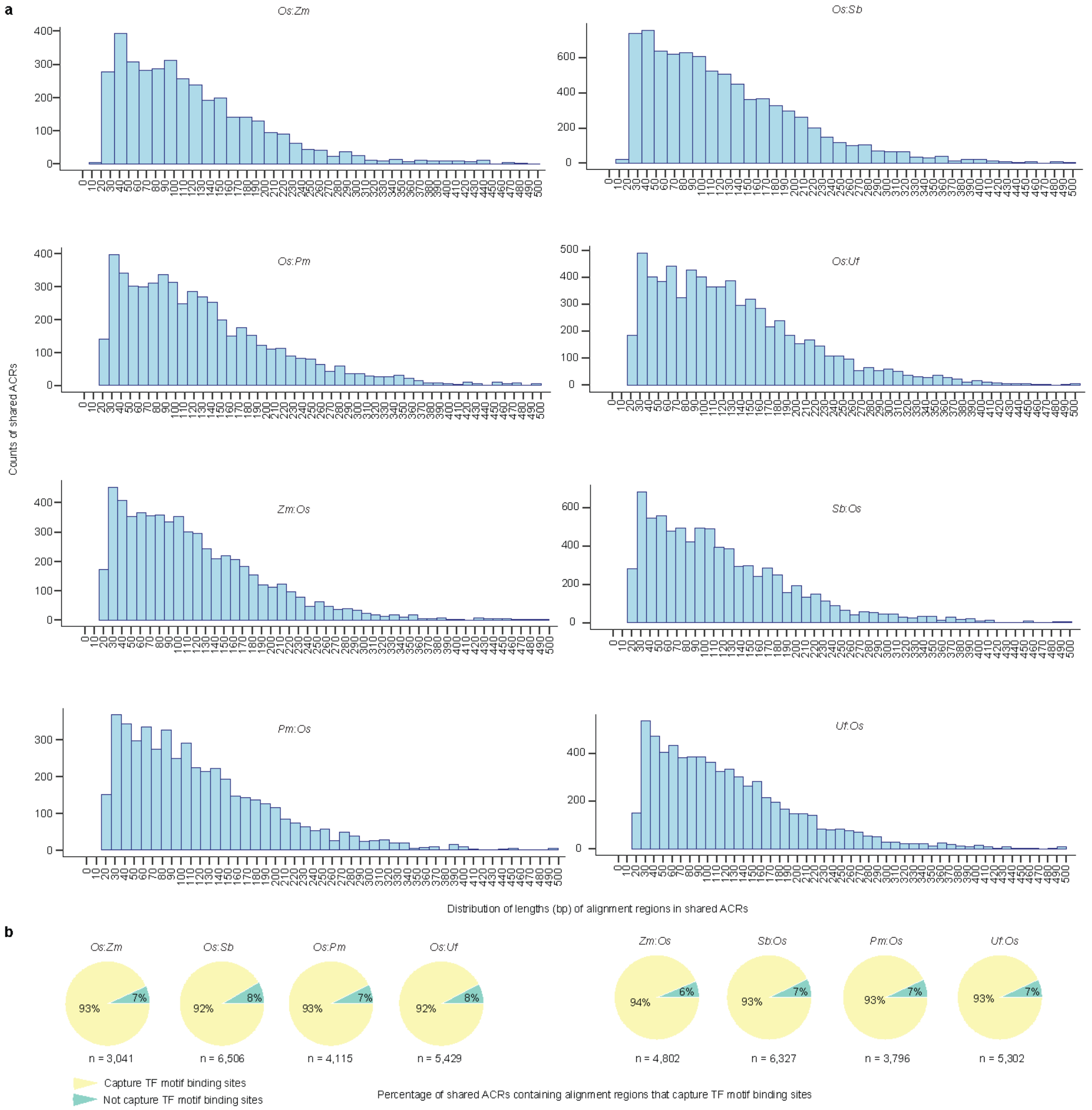

Supplementary Fig. 24. Alignment region length and TF motif bindings within shared ACRs between two species. a, Distribution of alignment region lengths (bp) within shared ACRs. **b,** Percentages of shared ACRs containing alignment regions that include TF motif binding sites.

References

1 Zong, J. *et al.* A rice single cell transcriptomic atlas defines the developmental trajectories of rice floret and inflorescence meristems. *New Phytologist* **234**, 494-512 (2022).

2 Hua, L. *et al.* The bundle sheath of rice is conditioned to play an active role in water transport as well as sulfur assimilation and jasmonic acid synthesis. *The Plant Journal* **107**, 268-286 (2021).

3 Itoh, J.-I. *et al.* Genome-wide analysis of spatiotemporal gene expression patterns during early embryogenesis in rice. *Development* **143**, 1217-1227 (2016).

4 Wu, T.-Y., Müller, M., Gruissem, W. & Bhullar, N. K. Genome wide analysis of the transcriptional profiles in different regions of the developing rice grains. *Rice* **13**, 1-19 (2020).

5 Van Dijk, D. *et al.* Recovering gene interactions from single-cell data using data diffusion. *Cell* **174**, 716-729. e727 (2018).

6 Satija, R., Farrell, J. A., Gennert, D., Schier, A. F. & Regev, A. Spatial reconstruction of single-cell gene expression data. *Nature biotechnology* **33**, 495-502 (2015).

7 Korsunsky, I. *et al.* Fast, sensitive and accurate integration of single-cell data with Harmony. *Nature methods* **16**, 1289-1296 (2019).

8 Mendieta, J. P. *et al.* Investigating the cis-regulatory basis of C3 and C4 photosynthesis in grasses at single-cell resolution. *Proceedings of the National Academy of Sciences* **121**, e2402781121 (2024).

9 Pavelescu, I. *et al.* A Sizer model for cell differentiation in Arabidopsis thaliana root growth. *Molecular systems biology* **14**, e7687 (2018).

10 Chen, Y., Sun, S. & Wang, X. The epidermis‐specific cyclin CYCP3; 1 is involved in the excess brassinosteroid signaling‐inhibited root meristem cell division. *Journal of Integrative Plant Biology* **62**, 1674-1687 (2020).
