# Supplementary material for: A single-cell rice atlas integrates multi-species data to reveal cis-regulatory evolution
Source: Nat Plants. 2025 Sep 17;11(10):2050–71. doi: 10.1038/s41477-025-02106-6 (PMC12537502; doi:10.1038/s41477-025-02106-6)
Supplement: Supplementary file 2 — Reporting Summary [file 41477_2025_2106_MOESM2_ESM.pdf]

nature portfolio

Corresponding author(s): Robert J Schmitz, Haidong Yan

Last updated by author(s): Aug 5, 2025

Reporting Summary

Nature Portfolio wishes to improve the reproducibility of the work that we publish. This form provides structure for consistency and transparency in reporting. For further information on Nature Portfolio policies, see our Editorial Policies and the Editorial Policy Checklist.

Statistics

For all statistical analyses, confirm that the following items are present in the figure legend, table legend, main text, or Methods section.

n/a	Confirmed	
☐	☒	The exact sample size (*n*) for each experimental group/condition, given as a discrete number and unit of measurement
☐	☒	A statement on whether measurements were taken from distinct samples or whether the same sample was measured repeatedly
☐	☒	The statistical test(s) used AND whether they are one- or two-sided<br>*Only common tests should be described solely by name; describe more complex techniques in the Methods section.*
☐	☒	A description of all covariates tested
☐	☒	A description of any assumptions or corrections, such as tests of normality and adjustment for multiple comparisons
☐	☒	A full description of the statistical parameters including central tendency (e.g. means) or other basic estimates (e.g. regression coefficient) AND variation (e.g. standard deviation) or associated estimates of uncertainty (e.g. confidence intervals)
☐	☒	For null hypothesis testing, the test statistic (e.g. *F*, *t*, *r*) with confidence intervals, effect sizes, degrees of freedom and *P* value noted<br>*Give P values as exact values whenever suitable.*
☒	☐	For Bayesian analysis, information on the choice of priors and Markov chain Monte Carlo settings
☐	☒	For hierarchical and complex designs, identification of the appropriate level for tests and full reporting of outcomes
☐	☒	Estimates of effect sizes (e.g. Cohen's *d*, Pearson's *r*), indicating how they were calculated

Our web collection on statistics for biologists contains articles on many of the points above.

Software and code

Policy information about availability of computer code

Data collection	Code Availability<br>The code used for the analyses throughout the manuscript is available on GitHub: https://github.com/yanhaidong1/scATAC-seq_cross_species
Data analysis	snRNA-seq/scRNA-seq analysis: Cellranger (v7.0.0), Seurat (v4.0), Scrublet, edgeR (v3.38.1).<br>Slide-seq analysis: Slide-seq pipeline (https://github.com/MacoskoLab/slideseq-tools), Seurat (v4.0).<br>Raw reads processing (scATAC-seq): cellranger-atac (v1.2.0), SAMtools (v1.7), Picard (v2.16.0), RepeatMasker (v4.1.2), BLAST+ (v2.11.0).<br>Identify high-quality nuclei (scATAC-seq): MACS2 (v2.2.7.1), Socrates.<br>Nuclei clustering (scATAC-seq): Socrates.<br>Estimation of gene accessibility scores: Seurat (v4.0).<br>Cell type validation: MAGIC algorithm, Seurat (v4.0), Harmony algorithm (v0.1.0).<br>ACR identification: MACS2 (v2.2.7.1), NucleoATAC (v0.2.1).<br>Identification of cell-type-specific ACRs: MACS2 (v2.2.7.1), edgeR (v3.38.1).<br>Correlation between chromatin accessibility of TF genes and motif deviation: PlantTFDB (v4.0), BLAST+ (v2.11.0), chromVAR (v1.18.0).<br>Linear-model based motif enrichment analysis: MEME suite (v5.1.1), JASPAR (v9).<br>De novo motif analysis: MEME suite (v5.1.1).<br>Identification of syntenic regions: GENESPACE (v1.4), BLASTN (v2.13.0), Seqkit.<br>Estimation of conservation scores: PhyloP (v1.0), PhyloFit (v1.0).<br>ChIP-seq analysis: bowtie2 (v2.5.2), SAMtools (v1.7), RiceENCODE.<br>DNA methylation analysis: Trimmomatic v0.363, Bismark v0.22.3.

GO enrichment test: AgriGO (v2).
Home-made scripts: https://github.com/yanhaidong1/scATAC-seq_cross_species/tree/main.

For manuscripts utilizing custom algorithms or software that are central to the research but not yet described in published literature, software must be made available to editors and reviewers. We strongly encourage code deposition in a community repository (e.g. GitHub). See the Nature Portfolio guidelines for submitting code & software for further information.

Data

Policy information about availability of data

All manuscripts must include a data availability statement. This statement should provide the following information, where applicable:

- Accession codes, unique identifiers, or web links for publicly available datasets
- A description of any restrictions on data availability
- For clinical datasets or third party data, please ensure that the statement adheres to our policy

scATAC-seq raw data encompassing 18 libraries from nine organs were accessible in NCBI (PRJNA1007577/GSE252040; https://dataview.ncbi.nlm.nih.gov/object/PRJNA1007577?reviewer=kgarq48dii11vomg44kgr1jq66). snRNA-seq/slide-seq raw data were accessible in NCBI (PRJNA1052039; https://dataview.ncbi.nlm.nih.gov/object/PRJNA1052039?reviewer=flhu9sl84o5m999r1ph8tlmmbg).

Research involving human participants, their data, or biological material

Policy information about studies with human participants or human data. See also policy information about sex, gender (identity/presentation), and sexual orientation and race, ethnicity and racism.

Reporting on sex and gender	not applicable
Reporting on race, ethnicity, or other socially relevant groupings	not applicable
Population characteristics	not applicable
Recruitment	not applicable
Ethics oversight	not applicable

Note that full information on the approval of the study protocol must also be provided in the manuscript.

Field-specific reporting

Please select the one below that is the best fit for your research. If you are not sure, read the appropriate sections before making your selection.

☒ Life sciences ☐ Behavioural & social sciences ☐ Ecological, evolutionary & environmental sciences

For a reference copy of the document with all sections, see nature.com/documents/nr-reporting-summary-flat.pdf

Life sciences study design

All studies must disclose on these points even when the disclosure is negative.

Sample size	scATAC-seq data for rice atlas: 18 libraries that include 9 organs with 2 biological replicates. snRNA-seq/slide-seq data for rice atlas: 4 libraries that include 1 organ with 4 biological replicates. Slide-seq: 1 library that includes 1 organ.<br>scATAC-seq data for C4 species downloaded from publications: 8 libraries from 1 organ with 2 biological replicates that include four C4 species.<br>scRNA-seq data for rice downloaded from publications: 2 libraries that include 1 organ with 2 biological replicates.<br>snRNA-seq data for maize from publications: 2 libraries that include 1 organ with 2 biological replicates.<br>snRNA-seq data for Arabidopsis from publications: 2 libraries that include 1 organ with 2 biological replicates.<br>DNA methylation for rice and maize from publications: 1 library for rice and 1 library for maize.<br>ChIP-seq data from publications: 1 library from EMF2b, and 4 libraries from H3K27me3.
Data exclusions	No data was excluded from the analyses.
Replication	Two biological replicates for all scATAC-seq, scRNA-seq/snRNA-seq datasets. One replicate for slide-seq and ChIP-seq and DNA methylation data.
Randomization	not applicable
Blinding	not applicable

Reporting for specific materials, systems and methods

We require information from authors about some types of materials, experimental systems and methods used in many studies. Here, indicate whether each material, system or method listed is relevant to your study. If you are not sure if a list item applies to your research, read the appropriate section before selecting a response.

Materials & experimental systems

n/a	Involved in the study	
☒	☐	Antibodies
☒	☐	Eukaryotic cell lines
☒	☐	Palaeontology and archaeology
☒	☐	Animals and other organisms
☒	☐	Clinical data
☒	☐	Dual use research of concern
☐	☒	Plants

Methods

n/a	Involved in the study	
☒	☐	ChIP-seq
☒	☐	Flow cytometry
☒	☐	MRI-based neuroimaging

Dual use research of concern

Policy information about dual use research of concern

Hazards

Could the accidental, deliberate or reckless misuse of agents or technologies generated in the work, or the application of information presented in the manuscript, pose a threat to:

No	Yes	
☒	☐	Public health
☒	☐	National security
☒	☐	Crops and/or livestock
☒	☐	Ecosystems
☒	☐	Any other significant area

Experiments of concern

Does the work involve any of these experiments of concern:

No	Yes	
☒	☐	Demonstrate how to render a vaccine ineffective
☒	☐	Confer resistance to therapeutically useful antibiotics or antiviral agents
☒	☐	Enhance the virulence of a pathogen or render a nonpathogen virulent
☒	☐	Increase transmissibility of a pathogen
☒	☐	Alter the host range of a pathogen
☒	☐	Enable evasion of diagnostic/detection modalities
☒	☐	Enable the weaponization of a biological agent or toxin
☒	☐	Any other potentially harmful combination of experiments and agents

Plants

Seed stocks	All the rice seeds were from USDA National Plant Germplasm System.
Novel plant genotypes	not applicable
Authentication	not applicable
